# High-throughput site-specific *N*-glycoproteomics reveals glyco-signatures for liver disease diagnosis

**DOI:** 10.1093/nsr/nwac059

**Published:** 2022-04-05

**Authors:** Zhenyu Sun, Bin Fu, Guoli Wang, Lei Zhang, Ruofan Xu, Ying Zhang, Haojie Lu

**Affiliations:** Shanghai Cancer Center and Institutes of Biomedical Sciences, Fudan University, Shanghai 200032, China; Department of Chemistry and NHC Key Laboratory of Glycoconjugates Research, Fudan University, Shanghai 200032, China; Shanghai Cancer Center and Institutes of Biomedical Sciences, Fudan University, Shanghai 200032, China; Shanghai Cancer Center and Institutes of Biomedical Sciences, Fudan University, Shanghai 200032, China; Eleanor Roosevelt College, University of California San Diego, La Jolla, CA92093, USA; Shanghai Cancer Center and Institutes of Biomedical Sciences, Fudan University, Shanghai 200032, China; Department of Chemistry and NHC Key Laboratory of Glycoconjugates Research, Fudan University, Shanghai 200032, China; Shanghai Cancer Center and Institutes of Biomedical Sciences, Fudan University, Shanghai 200032, China; Department of Chemistry and NHC Key Laboratory of Glycoconjugates Research, Fudan University, Shanghai 200032, China

**Keywords:** glycoproteomics, high-throughput, quantification, *N*-glycopeptides, liver disease

## Abstract

The glycoproteome has emerged as a prominent target for screening biomarkers, as altered glycosylation is a hallmark of cancer cells. In this work, we incorporated tandem mass tag labeling into quantitative glycoproteomics by developing a chemical labeling-assisted complementary dissociation method for the multiplexed analysis of intact *N*-glycopeptides. Benefiting from the complementary nature of two different mass spectrometry dissociation methods for identification and multiplex labeling for quantification of intact *N*-glycopeptides, we conducted the most comprehensive site-specific and subclass-specific *N*-glycosylation profiling of human serum immunoglobulin G (IgG) to date. By analysing the serum of 90 human patients with varying severities of liver diseases, as well as healthy controls, we identified that the combination of IgG1-H3N5F1 and IgG4-H4N3 can be used for distinguishing between different stages of liver diseases. Finally, we used targeted parallel reaction monitoring to successfully validate the expression changes of glycosylation in liver diseases in a different sample cohort that included 45 serum samples.

## Teaser

A high-throughput intact glycopeptide quantification strategy (HTiGQs) is developed. HTiGQs identified several glyco-signatures from IgG that can be used for diagosis and distinguishing different stages of liver diseases.

## INTRODUCTION

Glycosylation is the most universal post-translational modification (PTM) of proteins and plays critical roles in regulating physiological and pathological processes [1,2]. Aberrant protein glycosylation, which includes changes in the number of proteins glycosylated, the degree of glycosylation and the structure of the glycans, can occur in certain biological processes and diseases, such as immunological disorders, dementia and cancer [3–5]. In recent years, mass spectrometry (MS)-based quantitative glycoproteomics has illuminated a better understanding of the function of glycoproteins in physiological and pathological processes and is becoming a notable method for screening potential biomarkers for the clinical diagnosis of cancer [6,7]. Advancements in MS techniques have facilitated the analysis of site-specific glycopeptides (intact *N*-glycopeptide), which enables the understanding of the location of glycosylation, the occupancy of each glycosylation site and which glycan structures are associated with a certain glycosylation site [8]. The revelation of such glycosylation microheterogeneity achieved by intact glycopeptide analysis may provide important clues for precision medicine and targeted therapy [9].

However, MS-based disease glycoproteomics, specifically for investigating site-specific glycosylation, is still in its infancy due to insufficient technology. The characterization of intact *N*-glycopeptides is largely hindered because of many glycopeptides present at very low concentrations in complex samples. The difficulty of detecting glycopeptides, even by highly sensitive MS instruments, is further exacerbated by not only the suppression of their ionization in the presence of non-glycosylated peptides but also their structural and compositional complexity. Therefore, the analysis of glycopeptides typically requires them to be separated from non-glycosylated peptides prior to detection by MS. However, even after being separated, how to interrogate glycopeptides using tandem MS to produce efficient fragments for successful peptide and glycan composition determination still remains a formidable task. Unlike the majority of other PTMs, which are associated with a fixed mass increase, glycosylation is complex due to the varied composition of glycans. Optimal MS/MS acquisition is supposed to generate comprehensive fragments for each intact glycopeptide, including both the glycan and the peptide fragments [10].

Multiple different dissociation methods have been developed to characterize glycopeptides. Higher-energy collisional dissociation (HCD) can produce glycan and b/y-type peptide backbone fragments but may result in loss of all or a portion of the peptide's glycan modifications during the activation process [11]. Electron-transfer dissociation (ETD) generates mostly c/z-type peptide backbone fragments that retain intact glycan moieties with few glycan dissociation events although ETD methods require supplemental activation and significantly more abundant precursor ions than the HCD methods [12,13]. Thereby, the combination of these two techniques has proved to be suitable for the identification of glycopeptides. In terms of quantification, label-based methods, especially those based on isobaric chemical labeling, can improve the throughput in a single experiment, which is highly advantageous for proteome quantification [9,14]. However, these techniques currently are intended for the analysis of unmodified peptides that were not suitable for the analysis of intact *N*-glycopeptides. Glycans dissociate more easily than reporter groups because glycosidic bonds absorb most of the collisional energy, which limits the generation of reporter ions. To solve this problem, multi-notch MS3 acquisition (SPS-MS3) was developed as an acquisition method for enabling the accurate quantification of intact *N*-glycopeptides [15,16]. However, this method concurrently results in a decreased coverage of the proteome due to the requirement of a third scan for quantification in each scan cycle, which undoubtedly reduces the rate of data acquisition [17]. In addition, only a small percentage of MS1 precursor ions are converted into MS3 reporter ions, which reduces the sensitivity of detection [18]. Therefore, it calls for the development of high-throughput glycopeptide characterization methods that can expand the scope of glycoproteomics, provide accurate quantification and facilitate the investigation of physiological and pathological processes.

**Figure 1.**
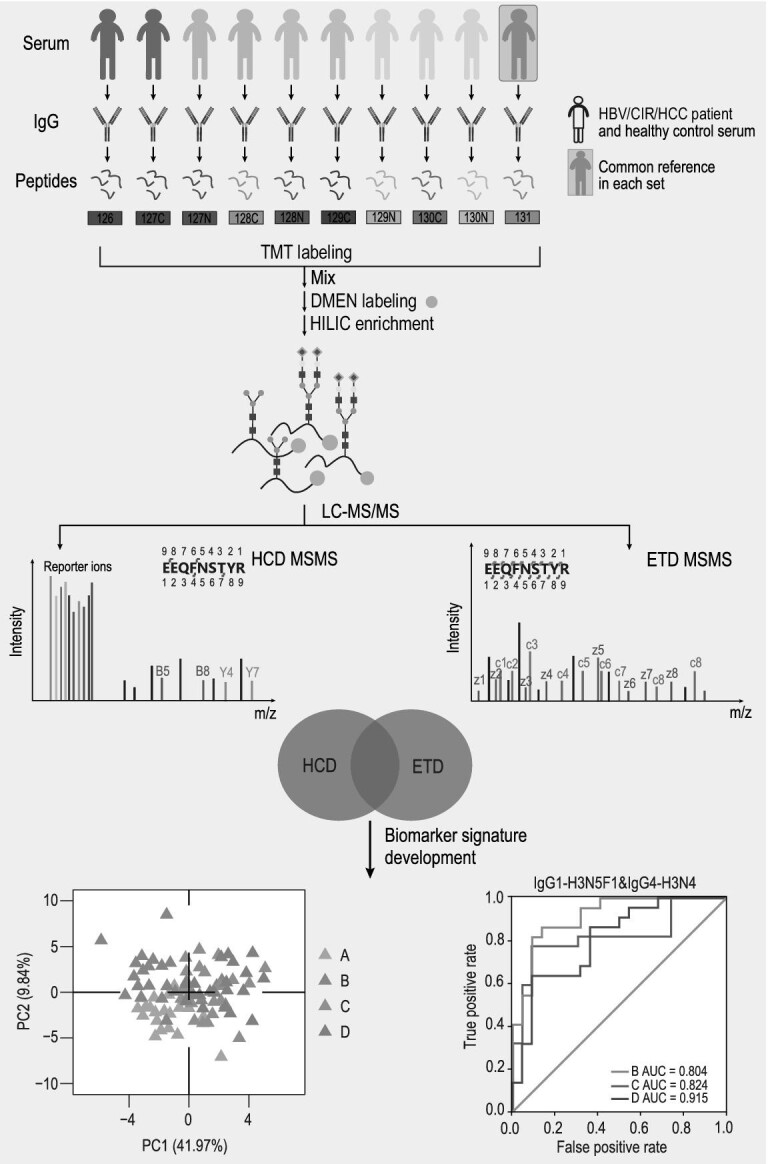
Scheme of HTiGQs for intact *N*-glycopeptide identification and quantification. The workflow of HTiGQs includes protein extract, preparation of TMT and DMEN-labeled glycopeptides, stepped collision energies HCD followed by product-dependent ETD (sceHCD-pd-ETD) acquisition for intact *N*-glycopeptide identification and quantification and the bioinformation analysis for biomarker discovery, (A) healthy controls, (B) HBV patients, (C) CIR patients and (D) HCC patients.

Herein, we describe the development of a high-throughput intact glycopeptides quantitation strategy (HTiGQs) for analysis of site-specific glycosylation in clinical samples (Fig. 1). This method comprised of two labeling steps. First, 10-plex TMT labeling was introduced to enable sample-multiplexing for quantifying 10 samples in a single experiment, thereby reducing the overall LC-MS measurement time. The use of tandem mass tag (TMT) labeling also avoided variations between replicates. Subsequently, *N, N*-dimethylethylenediamine (DMEN) labeling was performed to improve the charge states of the glycopeptides for enhancing their dissociation efficiency by ETD-MS/MS and HCD-MS/MS. Glycopeptides are inherently difficult to carry charges and their molecular weight is often >2000 Da, which makes them usually undergo non-dissociative electron transfer. Our previous work indicated that DMEN labeling increased the charge states of intact *N*-glycopeptides, providing abundant c/z ions for glycopeptide identification by ETD-MS/MS [19]. In addition, we utilized the HCD followed by product-dependent ETD (HCD-pd-ETD) acquisition modes for data acquisition to avoid wasting time on inefficient ETD acquisition. Based on these considerations, we systematically investigated the labeling efficiency, method of fragmentation, collisional energy and the balance between the intensity of the reporter ions and the identification capacity of the intact *N*-glycopeptides for determining the optimal parameters of the HTiGQs, thereby enhancing the accuracy of quantification and improving the depth of glycopeptide identification.

Hepatocellular carcinoma (HCC) is the second most common cause of cancer-related deaths [20,21]. Hepatitis B virus (HBV) infection is the leading cause of chronic liver diseases, including cirrhosis (CIR) and HCC [22]. Notably, HBV-related HCC accounts for ∼85% of all HCC cases in China due to the high prevalence of HBV infection. Considering that glycosylation is a hallmark of the immune system, we used our strategy to analyse the changes of IgG *N*-glycopeptides in the human serum of 90 individuals diagnosed with chronic HBV infection, CIR and HCC, and compared them to healthy individuals as a control (HC). In total, 313 intact *N*-glycopeptides from IgG were identified, which represents the largest human serum IgG subclass-specific and site-specific *N*-glycosylation data set to date. After further screening, the combination of IgG1-H3N5F1 and IgG4-H4N3 was identified as having the capability of distinguishing patients with HBV, CIR and HCC from healthy individuals, which indicated that these intact *N*-glycopeptides had the potential to be useful for non-invasive monitoring and pre-stratification of liver diseases. Finally, we used a parallel reaction monitoring (PRM) strategy to successfully validate the expression changes in the glycosylation between stages of liver diseases in a different sample cohort that included 34 serum samples from patients with different liver disease states and 11 healthy controls. In summary, the HTiGQs was successfully able to profile the site-specific heterogeneity of glycosylation in large-scale clinical samples, which provided not only a high-quality clinical glycoproteomic methodology but also important insight for developing precision medicine and tumor-targeted therapy.

## RESULTS AND DISCUSSION

### HTiGQs enhanced the identification of intact *N*-glycopeptides

As shown in Fig. 1, we utilized 10-plex TMT for glycopeptide labeling to allow multiplexed relative quantitation. After the glycopeptides were labeled by TMT, DMEN was added to react with the carboxyl groups from peptides and sialic acids to increase the number of charge states on the intact *N*-glycopeptides in the ETD-MS/MS mode [19]. Using commercially available IgG standards, we optimized the efficiency of these two labeling reactions. In theory, after TMT labeling, a 229.17-Da mass shift was observed due to the reaction of the reagent with the *N*-termini or the lysine ϵ-amino groups on the intact *N*-glycopeptides. Furthermore, labeling with DMEN introduced an additional 70.09-Da mass shift to the total mass after reacting with the carboxyl group of the *N*-glycopeptides. The details of the chemical reactions and the carboxyl groups involved in the derivatization are shown in Fig. S1. Using one *N*-glycopeptide as an example, the native intact *N*-glycopeptide IgG1-H4N4F1 (EEQYNSTYR with the glycan composition of Hex4HexNAc4Fuc1) yielded a [M + H]^+^ fragment with a mass of m/z = 2796.30 (Fig. S2). After TMT labeling, the glycopeptide was detected as a [M + H]^+^ fragment with a mass of m/z = 3025.67 and then as a [M + H]^+^ fragment with a mass of m/z = 3235.36, which indicated that the TMT-labeled glycopeptide was labeled with three DMEN molecules: one at the C-terminus and two onto Glu residues. The unlabeled glycopeptide was not detected after TMT and DMEN labeling, indicating that the *N*-glycopeptide was labeled efficiently under these conditions. To further confirm the success of labeling, we analysed the labeled sample using LC-MS/MS with HCD-MS/MS and ETD-MS/MS acquisition modes, respectively. The Byonic search engine was used for *N*-glycopeptide identification and the IgG glycan database is summarized in Table S1 [5]. As expected, the HCD-MS/MS mode led to the production of oxonium ions, glycan fragments and TMT reporter ions, which further corroborated the successful introduction of TMT labels. ETD-MS/MS enabled the generation of abundant c/z ions of the peptide backbone and a z1 ion with a mass of m/z = 229.18 was observed, both of which indicated that the *N*-glycopeptide was labeled with DMEN successfully (Fig. S3).

**Figure 2.**
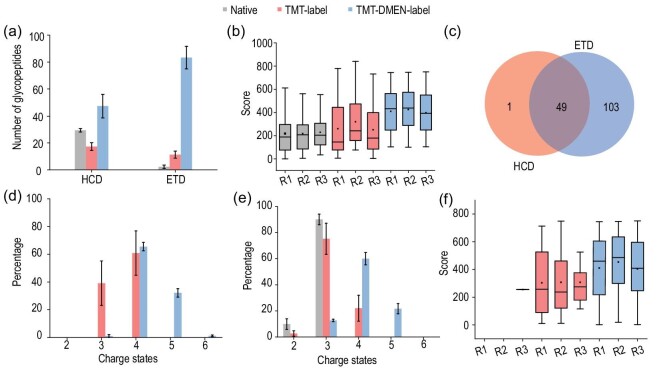
The identification capability for intact *N*-glycopeptide using HTiGQs. (a) The distribution of the number of identified *N*-glycopeptides from native, TMT labeling and TMT-DMEN labeling samples acquired in HCD-MS/MS and ETD-MS/MS acquisition mode. (b) The boxplot of Byonic scores of identified *N*-glycopeptides from HCD spectra. (c) The overlap of glycopeptides identified from HCD and ETD spectra across three technical replicates. (d) The distribution of charge states of identified *N*-glycopeptides from native, TMT labeling and TMT-DMEN labeling samples identified from ETD spectra. (e) The distribution of charge states of identified *N*-glycopeptides from native, TMT labeling and TMT-DMEN labeling from HCD spectra. (f) The boxplot of Byonic scores of identified *N*-glycopeptides from ETD spectra.

Next, we systematically compared the identification results of the *N*-glycopeptides with and without labeling. Without labeling, a total of 31 non-redundant *N*-glycopeptides were identified in the HCD and ETD spectra; among them, only 2 *N*-glycopeptides were identified in the ETD spectra. After labeling, 103 non-redundant *N*-glycopeptides were identified, which was three times more than the number of *N*-glycopeptides identified before labeling (Fig. 2a). In particular, a significant increase in the number of glycopeptides was observed in the ETD-MS/MS mode. In addition, the average Byonic score of the glycopeptides after the two labeling steps was higher than the score of unlabeled glycopeptides (Fig. 2b). After comparing the number of glycopeptides identified in both the HCD and ETD spectra, we noticed that more glycopeptides were identified by ETD-MS/MS compared to by HCD-MS/MS. Furthermore, >98% of the glycopeptides identified in the HCD spectra were also identified in the ETD spectra, indicating that the combination of the ETD and HCD acquisition modes was crucial for identifying as many glycopeptides as possible (Fig. 2c). We further analysed the reasons for the significant increase in the number of identifications by ETD-MS/MS compared to HCD-MS/MS. The efficiency of detection by ETD-MS/MS was directly influenced by the number of charges of the glycopeptides. Consequently, the number of glycopeptides identified increased after TMT labeling because the conversion of the primary amines of the lysine residues in the native peptides into a moiety containing a dimethyl piperidine group increased the proton affinity of these residues, thereby increasing the number of possible charge states during ionization [23]. As a result, most of the TMT-labeled glycopeptides were observed as having +3 and +4 charge states (Fig. 2d). Furthermore, we observed that DMEN labeling significantly increased the number of charge states of the glycopeptides to +4 and +5. Therefore, the number of identified glycopeptides increased dramatically after DMEN labeling. We also observed that the formation of charge states on the identified glycopeptides after TMT labeling increased during HCD-MS/MS analysis and the charge states increased even more after TMT and DMEN labeling (Fig. 2e). The Byonic score of the *N*-glycopeptides identified in the ETD spectra also increased significantly compared to unlabeled and TMT-labeled *N*-glycopeptides, with a medium score of 451.57, which corroborated that DMEN labeling increased the efficiency of detection by ETD-MS/MS (Fig. 2f). When the glycopeptides only underwent TMT labeling, the number of peptides identified and the corresponding Byonic score were slightly lower than unlabeled *N*-glycopeptides in HCD-MS/MS. This result might have been attributed to the high collision energies in HCD-MS/MS, which caused some glycans to produce excessive fragments, which increased the complexity of the HCD spectra. After DMEN labeling, the number of glycopeptides identified was higher than unlabeled and TMT-labeled *N*-glycopeptides, indicating that the increase in charge states also facilitated the identification of glycopeptides by HCD-MS/MS.

The high ETD efficiency enabled the elucidation of the peptide sequences and site-specific glycosylation analysis. For an example, the *N*-glycopeptides of IgG3 and IgG4 (IgG3, EEQYNSTFR and IgG4, EEQFNSTYR) only differed by the position of Y and F residues in the sequence. Therefore, these two *N*-glycopeptides were difficult to be distinguished by chromatographic separation or by MS according to their m/z [24]. To ensure proper detection, IgG3 typically needed to be pre-separated using antibodies to differentiate the IgG3 from the IgG4. Using this method, extensive c-type and z-type fragmentation along the peptide backbone occurred upon ETD fragmentation, which enabled the discrimination of the *N*-glycopeptides from different IgG subclasses directly in the mass spectra. For example, 100% sequence coverage of the glycopeptide EEQFNSTYR was obtained after TMT and DMEN labeling, and we confirmed its subclass based on the c8 and z2 ions (glycopeptide EEQFNSTYR, where N* was the glycosylation site modified with H3N5F1) (Fig. S4). In summary, these results revealed that the HTiGQs improved the identification of *N*-glycopeptides and provided the foundation for enabling the high-throughput characterization and quantitation of intact *N*-glycopeptides.

### HTiGQs improved the quantification of intact *N*-glycopeptides

Although TMT labeling can improve the throughput in a single experiment, the challenge for intact glycopeptides quantification by HCD-MS/MS is that the less intense reporter ions of the labeled glycopeptides are observed compared to labeled non-glycopeptides. Stepped collision energies HCD-MS/MS (sceHCD) method has been demonstrated to be advantageous in glycopeptides identification using 20–30–40% collision energies, in which the most informative and abundant fragment ions for both the glycan and peptide in a single spectrum are generated [25,26]. However, the high-intensity TMT reporter ions are observed at a normalized collision energy (NCE) of >45% because the glycosidic bonds absorb most of the collisional energy upon fragmentation by HCD-MS/MS rather than the labeled linear peptides. Low reporter ion intensities will compromise the sensitivity and reproducibility of quantification. To balance the relationship between the intensity of the reporter ions and the number of glycopeptides identified, we investigated the effects of the combination of different HCD collision energies and ETD trigger acquisition methods, including sceHCD-ETD, sceHCD-pd-ETD and HCD-ETD, with different HCD collision energies on the identification and quantification of intact *N*-glycopeptides.

**Figure 3.**
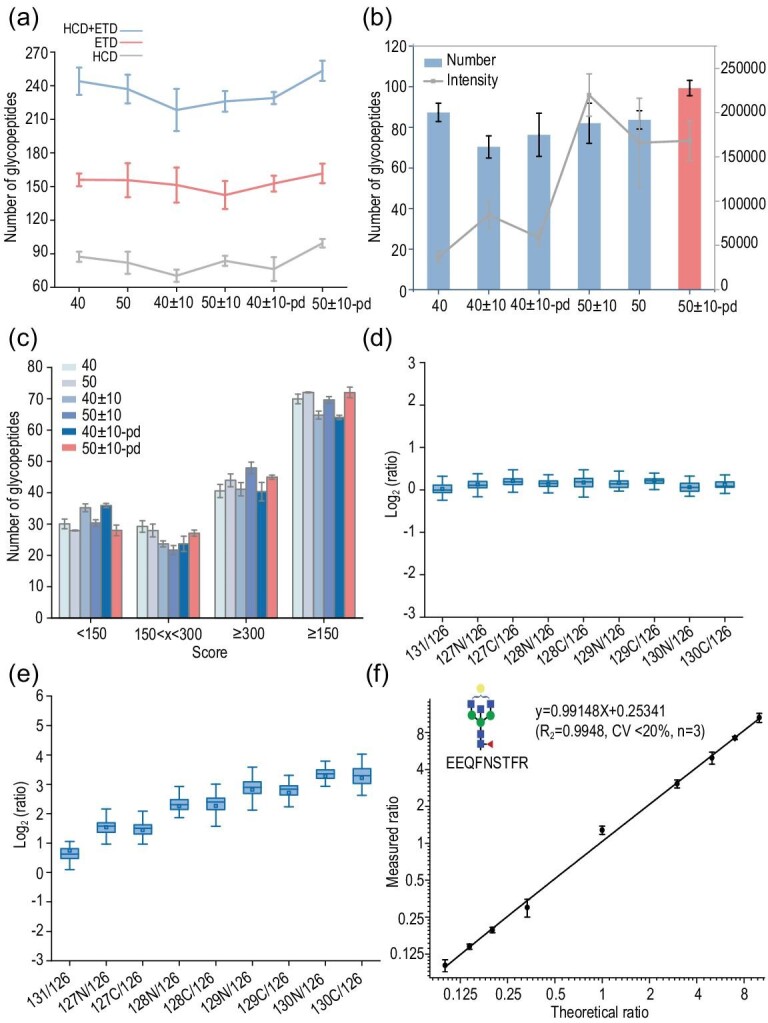
Characterization of the identification capacity and quantification accuracy of HTiGQs. (a) Average number of identified intact *N*-glycopeptides from different dissociation methods. (b) The relationship between average number of identified intact *N*-glycopeptides and the intensity of reporter ions from HCD spectra. (c) The distribution of Byonic score of identified intact *N*-glycopeptides from HCD spectra using different six fragmentation methods. (d) Boxplots display the ratio distribution of the quantified glycopeptide ratios using HTiGQs with proportion of 1:1:1:1:1:1:1:1:1:1. (e) Boxplots display the ratio distribution of the quantified glycopeptide ratios using HTiGQs with proportion of 1:3:3:5:5:7:7:10:10:1.5. (f) Plot of the theoretical molar ratios vs. the measured ratios of IgG2-H4N5F1.

Due to variations in the normalized collision energies among these three dissociation methods, we created six different MS/MS acquisition methods for comparison, the details of which are listed in Table S2. The average number of identified, intact *N*-glycopeptides from each method is summarized in Fig. 3a. Employing different collision energies in HCD-MS/MS undoubtedly had an impact on glycopeptide identification. Two of the six methods with NCE ± 40% were less capable of identification than the other four methods, likely because the energy utilization was <40%. ETD trigger methods also influenced glycopeptide identification. The HCD-pd-ETD methods outperformed the direct ETD acquisition methods. The highest number of glycopeptides identified was using the sceHCD-pd-ETD with NCE 50 ± 10 method, which had >120 non-redundant glycopeptides identified from the HCD and ETD spectra. Next, we compared both the intensities of the TMT reporter ions and the number of glycopeptides. An increase in the collision energy significantly improved the intensities of the TMT reporter ions. Based on these results, we chose the sceHCD-pd-ETD 50 ± 10 method as the optimal acquisition method (Fig. 3b). For the glycopeptides only identified from ETD spectra, the quantitation of the glycopeptides was obtained in the paired HCD spectra. We also calculated the intensities of the TMT reporter ions, the results of which were consistent with the results from the HCD spectra (Fig. S5). The Byonic score distribution of these methods further proved that the sceHCD-pd-ETD 50 ± 10 method generated the most identifications with a Byonic score of ≥150 (Fig. 3c). In summary, the sceHCD-pd-ETD 50 ± 10 method was chosen as the optimal acquisition method because it manifested the best identification efficiency and provided accurate quantification.

To evaluate the dynamic range and accuracy of the HTiGQs, we used commercial IgG standards to evaluate. The IgG standards were digested with trypsin, and the peptides of IgG standards were divided into 10 equal amounts and labeled separately with individual TMT reagents (126–131). Then, the mixture was incubated with DMEN and analysed by LC-MS/MS using the optimized fragmentation method. A total of 139 non-redundant intact *N*-glycopeptides of IgG were identified after three replicate measurements, including 30 from IgG1, 44 from IgG2, 31 from IgG3 and 34 from IgG4 (Table S3). As shown in Fig. 3d, the median ratios of the equally mixed intact *N*-glycopeptides were 0.996, 1.081, 1.139, 1.110, 1.138, 1.103, 1.161, 1.039 and 1.063, respectively (the 126 channel was set as the reference). Three replicate measurements are displayed in Fig. S6, indicating the high reproducibility of this quantification method. The standard deviation (SD) histograms of the quantified glycopeptides ratios from the three replicate experiments are shown in Fig. S7, exhibiting the high accuracy of this strategy. To further assess the accuracy of this quantitation strategy, a mixture of glycopeptides of IgG standards with a mass ratio of 1:3:3:5:5:7:7:10:10:1.5 were analysed by LC-MS/MS. The quantitative ratios for these intact *N*-glycopeptides are recorded in Table S4. The median ratios of the intact *N*-glycopeptides were 3.019, 2.833, 4.963, 5.276, 7.463, 7.155, 10.247, 10.116 and 1.548, respectively (channels 127C and 127N to 126 were 3:1, 128C and 128N to 126 were 5:1, 129C and 129N to 126 were 7:1, 130C and 130N to 126 were 10:1, and 131 to 126 were 1.5:1) (Fig. 3e). As shown in Fig. 3f, the detected ratio of the intact *N*-glycopeptide IgG2-H4N5F1 was fairly consistent with the expected ratio, indicating that HTiGQs has a high reproducibility (CV < 20%) and good linearity (*R*^2^ > 0.99) in quantitation within one order of magnitude. These results demonstrated that our HTiGQs enabled the accurate quantification of intact glycopeptides and has the potential for high-throughput and in-depth glycoproteomics analysis in more complex biological samples.

### Large-scale serum glycoproteome characterization by HTiGQs

**Figure 4.**
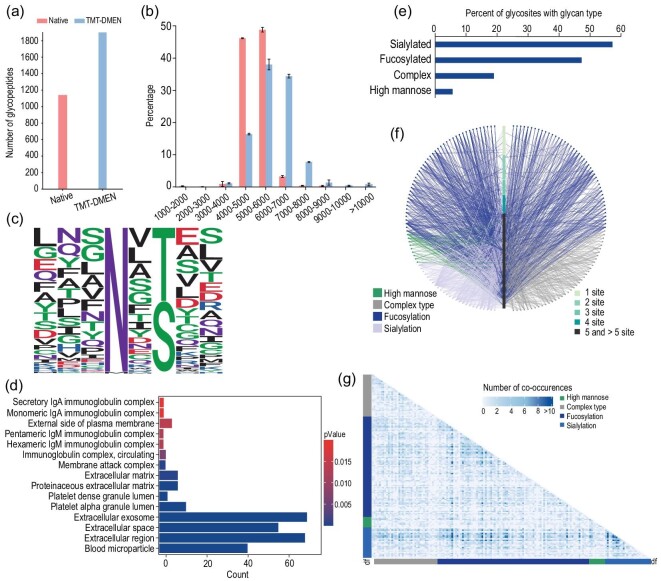
Characteristics of intact *N*-glycopeptides from human serum revealed by HTiGQs. (a) The number of identified intact *N*-glycopeptides. (b) Molecular weight distribution of intact *N*-glycopeptides identified from human serum. (c) Sequence motifs distribution for *N*-glycosylation sites having either the N–X–S or N–X–T sequon. (d) GO analysis of identified glycopeptides from human serum in terms of cellular component. (e) Percentage of total glycosylation sites modified with glycans of high mannose, sialic acids, fucose and complex. (f) A glycoprotein–glycan network map shows the correspondence between glycans and its modified proteins, and which glycans (outer circle) modify which proteins (inner bar). Glycoproteins are sorted by the number of glycosylation sites. Glycans are organized by glycan types and edges are colored by the glycan types. (g) A glycan co-occurrence heat map represents the frequency of glycan pairs appearing together at the same glycosylation sites, indicating which glycan contributes most to the microheterogeneity.

To test the suitability of the HTiGQs workflow for the analysis of glycosylation in complex biological samples, we applied this strategy to study the glycoproteome in human serum. After collecting 10 μL of serum from a participant, serum proteins were extracted, digested with trypsin and Glu-C, and analysed by HTiGQs. In total, we identified 1961 unique *N*-glycopeptides that were mapped to 168 glycoproteins with 182 different glycan compositions (Table S5). Compared to the native intact *N*-glycopeptides, the number of intact *N*-glycopeptides after labeling increased by >75% (Fig. 4a). Because DMEN labeling increased the charge states of the glycopeptides during ETD-MS/MS analysis, we elucidated the distribution of the charge states of the glycopeptides obtained from the human serum after DMEN labeling. Approximately 80% of the charge states of glycopeptides before labeling were observed as +3, but after DMEN labeling, the charge states existed predominantly as +4, +5, +6 and +7 (Fig. S8a). Based on the molecular weight distribution of the glycopeptides (Fig. 4b), DMEN labeling facilitated the identification of glycopeptides with higher molecular weights. Therefore, DMEN labeling directly improved the identification of glycopeptides. Since multi-mode fragmentation allowed the acquisition of complementary fragmentation spectra on the same timescale of eluting peptide species [12,13], we thereby summarized the peptide spectrum matches (PSM) number of intact *N*-glycopeptides from each mode; 55% of the PSMs (3250) was derived from ETD spectra, while the other 45% (2640) was derived from HCD spectra. These results indicated that the combination of ETD and HCD acquisition modes provided complementarity in the identification of serum glycopeptides for facilitating the generation of an in-depth serum glycoproteomic database (Fig. S8b). To investigate whether the different acquisition modes had a preference toward certain types of glycans, the total identified glycopeptides were grouped into high-mannose and complex categories, which were further divided based on the number of fucose and sialic acid units (Fig. S8c). More high-mannose glycans and complex type with one fucose were identified in the HCD acquisition mode, while a more complex type containing one fucose and sialic acids and a complex type containing sialic acids were identified in the ETD acquisition mode. Other types of glycans exhibited no significant differences. In summary, the combination of the HCD and ETD acquisition modes provided complementarity for facilitating the identification of intact *N*-glycopeptides.

The ability to profile glycosylation sites with intact glycans provided opportunities to investigate system-wide glycosylation patterns. Sequons of the identified *N*-glycopeptides showed expected N–X–S and N–X–T sequons (Fig. 4c). Gene ontology (GO) enrichment of the cellular components from the identified glycoproteins was performed using the clusterProfile (version 3.16.0) package in R, which revealed many of the expected enriched terms, including blood microparticle, extracellular region/space and extracellular exosome protein-related terms (Fig. 4d). As shown in Fig. 4e, which displays the percentages of the glycosylation sites featuring high-mannose glycans, complex-type glycans without fucose or sialic acid units, fucosylated glycans and sialylated glycans, we observed significantly high proportions of fucosylated and sialylated glycopeptides, as well as a low proportion of high-mannose glycans, in the serum, which was different from the previous results in which that tissue included predominantly high-mannose glycans [27]. Figure S8d displays a histogram of the average number of glycans per glycosylated site. The majority of the glycosylation sites identified contained one to three glycans, with the average number of glycans per site equal to 3.92. Approximately 10% of the identified glycosylation sites contained ≥10 *N*-glycans. These heterogeneous glycoproteins containing >10 *N*-glycans at one site in the serum included A1AT, HEMO and FERUA. Therefore, the site-specific glycopeptides analysis enabled us to understand the degree of heterogeneity across the glycoproteome and the biological contributions of these glycoproteins to the functions of complex systems.

Since intact *N*-glycopeptides analysis uniquely enables the characterization of site-specific microheterogeneity, a glycoprotein–glycan network diagram was developed and several discernible patterns appear (Fig. 4f). The glycoproteomic data set revealed that fucosylation and sialylation were the most observed glycosylation patterns in the serum. The network diagram also highlighted which glycan structures contributed more to heterogeneity. For example, the majority of fucosylated glycans occurred on proteins with multiple glycosylation sites. Nearly half of the proteins studied featured more than five glycosylation sites, indicating that the glycoproteins in the serum were heterogeneous. Using the large-scale serum glycoproteomics data set obtained by our method, we generated glycan co-occurrence networks to display the co-occurrence frequency of specific glycans with all other glycans across various glycosylation sites (Fig. 4g). In this diagram, the squares represent the co-occurrence of two glycans at the same glycosylation site and the color denotes the frequency of the co-occurrence. Fucosylated glycans co-occurred together frequently and they co-occurred with several other groups of complex and sialylated glycans. Overall, these data indicated that the HTiGQs was capable of large-scale characterization of intact *N*-glycopeptides in complex biological samples, such as serum. The glycoproteomics data also revealed that fucosylation and sialylation were the most prominent glycosylation modifications and the serum glycoproteins were of complex heterogeneity, typically containing five or more glycosylation sites.

### Discovery of biomarkers of liver diseases using HTiGQs

A significant increase in serum immunoglobulin A (IgA) and IgG levels has been reported during hepatic fibrosis. IgG are the most abundant immunoglobulins in the circulatory system (∼10 mg/mL) and they are integral to the immune system, as they function to recognize and clear foreign antigens and pathogens [28]. Each IgG subclass has one highly occupied and conserved *N*-glycosylation site with a specific amino acid sequence. The *N*-glycosylation of IgG is associated with pathogenesis and the progression of diseases, and plays a critical role in the regulation of functional responses mediated by Ig-receptors and other interacting partners [4,29]. For example, patients with HCC exhibit a significantly higher fraction of *Lens culinaris agglutinin* bound to IgG (core-fucosylated IgG, IgG-L3) among total serum IgG [30]. However, previous studies lack the information about the subclass-specific and site-specific *N*-glycosylation of IgG, which is fundamental for precision therapeutics. In the previous retrospective study, only the relationship between the type of liver disease and HC or incomplete disease types were considered [19,30].

In this study, we collected serum samples from patients with different liver diseases, including HBV, CIR and HCC, to investigate the value of *N*-glycosylated IgG in the serum to identify glyco-signatures for distinguishing different stages of liver diseases to facilitate precision medicine and disease-targeted therapy. Herein, we applied the HTiGQs to analyse the intact *N*-glycopeptides of IgG isolated from the human serum samples, with the aim to identify novel biomarkers to distinguish HC, HBV, CIR and HCC. A total of 90 serum samples (HC = 23, HBV = 24, CIR = 22, HCC = 21) were collected and the basic demographic and clinical information of the participants is summarized in Table S6. All participants were recruited under protocols approved by the Affiliated Hospital of Xuzhou Medical University. HBV, CIR and HCC were diagnosed by an attending physician based on liver imaging and/or liver biopsies. IgG was isolated from each participant and purified using Protein A/G agarose beads. The SDS-PAGE results showed that the IgG obtained from the human serum was pure and the purification was reproducible (Fig. S9). For quality control (QC) of the LC-MS/MS performance, tryptic digestions of Hela cell lysate were measured as a QC standard every five runs. The QC standard was analysed using the same method and conditions. The calculated pairwise Spearman's correlation coefficient values between each QC run are shown in Fig. S10. The average correlation coefficient among the standards was 0.98, with maximum and minimum coefficients of 0.99 and 0.97, respectively. In total, 313 intact *N*-glycopeptides from IgG were identified, which represented the largest data set corresponding to the subclass-specific and site-specific *N*-glycosylation of IgG in human serum reported to date (Table S7).

**Figure 5.**
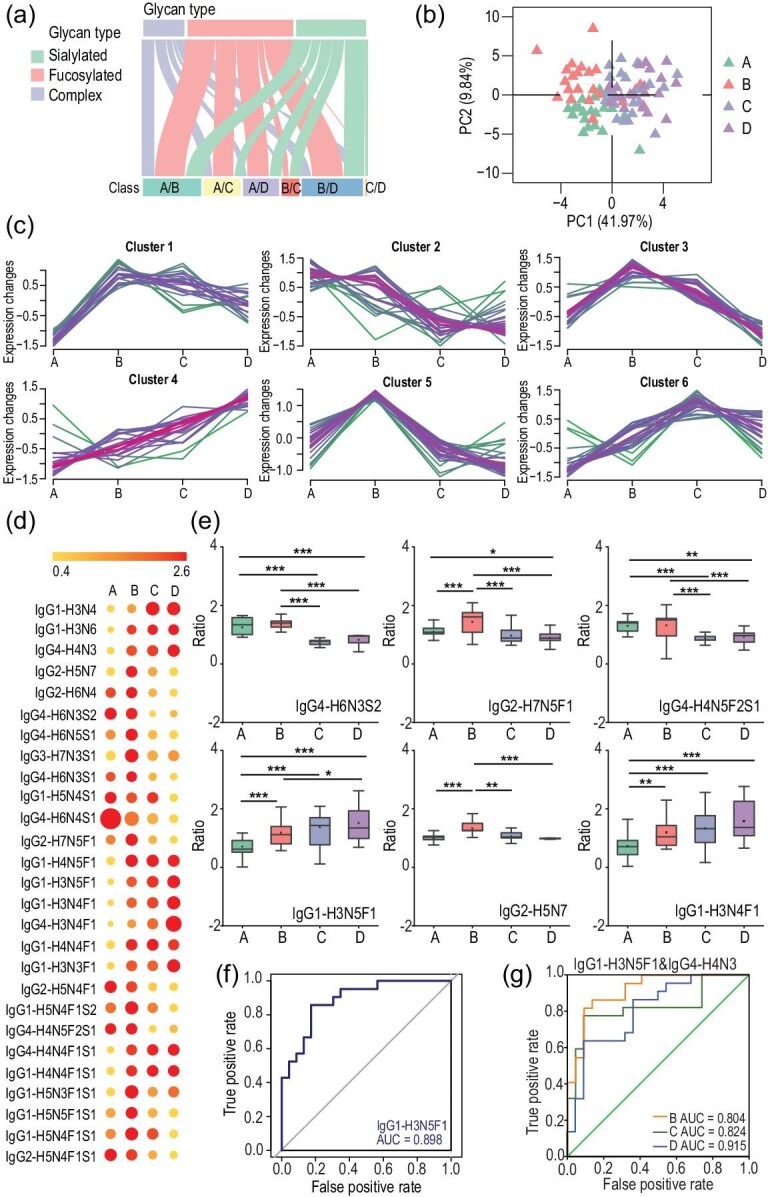
Identification of potential biomarker combinations for liver diseases by using HTiGQs. (a) A plot displays the differentially expressed glycans in different stages of liver disease. Glycans are organized by glycan types. (b) Partial least squares discrimination analysis (PLS-DA) based on the abundance of intact *N*-glycopeptides to reveal the difference among (A) 23 healthy controls, (B) 24 HBV patients, (C) 22 CIR patients and (D) 21 HCC patients. (c) 162 glycopeptides were clustered using mFuzz into significant discrete clusters, respectively. (A) healthy controls; (B) HBV; (C) CIR; (D) HCC; (d) A heat map displays the relative abundances of differentially expressed intact *N*-glycopeptides among (A) healthy controls, (B) HBV patients, (C) CIR patients and (D) HCC patients. Hex (H), HexNAc (N), Fuc (F), Sia (S) indicate hexose, *N*-acetylhexsamine, fucose and sialic acid, respectively. (e) The expression level change (relative abundance) of six selected intact *N*-glycopeptides with significant difference. Asterisks indicate statistical significance based on unpaired two-sided Welch's *t*-test. *P < 0.05; **P < 0.01; ***P < 0.001. (f) Receiver operating characteristic (ROC) curve of IgG1-H3N5F1 and AUC value was calculated for the classification of healthy controls and HCC patients. (g) ROC curves of the combination of IgG1-H3N5F1 and IgG4-H4N3. AUC values were calculated for the classification of (B) HBV, (C) CIR, (D) HCC and (A) healthy controls.

To identify specific glycopeptides associated with certain liver diseases, a two-tier screening approach for candidate selection was employed to narrow our initial targets. First, peptides having >50% missing data were excluded. After filtering, 162 glycopeptides were retained (Table S8). Based on the distribution of the glycan types associated with the different subclasses of IgG (Fig. S11), the high-mannose glycans were the least abundant, while the sialylated and fucosylated glycans were the most abundant. The second-tier screening was implemented to further filter the identified glycopeptides and retain only those that demonstrated significant changes between the HC and liver disease samples. The intact *N*-glycopeptides 50, 34 and 31 were significantly changed in the HC vs. HBV, HC vs. CIR and HC vs. HCC samples for further evaluation (with fold change of >1.25 or <0.8, *P* < 0.05) (Table S9) [31]. The types of glycan are shown in Fig. 5a. After comparing the differentially expressed glycopeptides derived from the different liver disease samples, the fucosylated glycopeptides were the most abundant glycopeptides in all samples and the high-mannose glycans were only observed in the CIR vs. HCC group. The top 15 ratios of the differentially expressed intact *N*-glycopeptides from each comparison group are shown in Fig. S12. The UpSet plot shows the overlap of differentially expressed intact *N*-glycopeptides between the different comparison groups (Fig. S13). Compared to the other groups, the HBV vs. HCC group has the greatest number of differentially expressed intact *N*-glycopeptides. We then used the partial least squares discrimination analysis (PLS-DA) to cluster these samples, which revealed the formation of distinct clusters in the HC, HBV, CIR and HCC samples (Fig. 5b). All HBV, CIR and HCC samples were assembled and basically differentiated from the HC samples. Following on, we evaluated the relationship between the expression change of intact *N*-glycopeptides and the disease severity by mFuzz (Table S10). Six clusters that highlighted that the expression of intact *N*-glycopeptides changed differently during the disease progression were formed (Fig. 5c). Different glycans at the same glycosylation sites have different trends in disease progression. For example, the abundance of IgG2-H5N5F1 decreased while IgG2-H3N5F1 increased. We also observed that fucosylation of IgG2 was significantly decreased from HC to CIR (H5N5F1, H5N5F1S1, H5N4F1, H5N4F1S1 and H4N5F1), but fucosylation of the other three subclasses of IgG was increased from HC to HBV while decreased from HBV to CIR (H5N5F1, H5N4F2, H5N4F1S1, H6N4F1S1, H5N4F1 and H4N6F1). These results suggested that the changes in glycosylation might have been attributed to the intact glycopeptide abundance rather than the glycan abundance, which suggested that assaying of site-specific glycoproteins in clinical samples could be more informative for the diagnosis and monitoring of liver disease severity than measuring glycan abundance alone.

To further determine the progress of IgG *N*-glycosylation, we selected differentially expressed intact *N*-glycopeptides in at least three groups for subsequent analysis as the candidate glycopeptides and a total of 27 intact *N*-glycopeptides met the minimum requirements, including 13 glycopeptides of IgG1, 5 glycopeptides of IgG2, 1 glycopeptide of IgG3 and 8 glycopeptides of IgG4. We calculated the average of the relative abundances of the selected intact *N*-glycopeptides from 90 individual samples and plotted the averages in Fig. 5d. The sialylated *N*-glycopeptides were found in low abundance in the HCC samples, but the fucosylated *N*-glycopeptides, except IgG2-H7N5F1 and IgG2-H5N4F1, were in high abundance in the HCC samples. An increase in the fucosylated *N*-glycopeptides comprising a galactose-deficient core (IgG1-H3N4F1 and IgG4-H3N4F1) was observed in HCC samples, which was consistent with a previous report [30]. Especially, we were able to confirm these results at the subclass-specific and site-specific levels. Several other studies revealed that core-fucosylation of IgG played an important role in improving the binding capacity of the Fc region to the FcϒRIIIa receptor [32]. Sialic acids of IgG were able to reduce the binding capacity of the antibody to the FcϒRIIIa receptor, thereby decreasing antibody-dependent cytotoxicity and enhancing its anti-inflammatory effects [33]. The 27 candidate glycopeptides were further analysed by using one-way analysis of variance (ANOVA) (*P* < 0.05). The top six glycopeptides were expressed as a boxplot (Fig. 5e), which displayed the differential expression at the individual levels (Table S11). All six glycopeptides were used to reliably classify the types of samples. Furthermore, to evaluate the discrimination power of the differentially expressed glycopeptides to distinguish between patients with HBV, CIR and HCC from the HC group, receiver operating characteristic (ROC) curves were constructed and the area under curve (AUC) values were calculated. The AUC from the ROC curve of the glycopeptide IgG1-H3N5F1 was 0.898, which indicated that the HCC group could be distinguished from the HC group (Fig. 5f). In addition, IgG1-H3N4F1 could also be utilized to distinguish HCC from HC (AUC = 0.892) and IgG2-H6N3F1 could be utilized to distinguish HBV from CIR (AUC = 0.855) (Fig. S14). After evaluating the glycopeptide signatures individually, we further investigated the potential of combining multiple glycopeptides for improving the clinical utility of these candidate glycopeptides. To better distinguish patients with different stages of liver disease from healthy individuals, we selected IgG1-H3N5F1 and IgG4-H4N3 as the candidate glycopeptides because they demonstrated the best performance among the candidate glycopeptides. We calculated the compact biomarker combination containing these two glycopeptides using logistic regression and the AUC values of the panel of the two glycopeptides combination used to distinguish the HBV, CIR and HCC groups from the HC group were 0.804, 0.824 and 0.915, respectively (Fig. 5g). When more candidate glycopeptides were added into the panel, the AUC of the panel following logistic regression was improved. When we selected five glycopeptides to the panel, the AUC increased to 0.903, 0.957 and 0.957, respectively (Fig. S15a–c). The new glyco-signature panel comprising the five glycopeptides was more sensitive and specific than when the panel comprised two glycopeptides. This new panel could distinguish different stages of liver diseases (e.g. HBV vs. CIR, HBV vs. HCC) in clinical samples and the AUC values were 0.888 and 0.970, respectively (Fig. S15d and e). Seven representative differential expressed glycopeptides that were found in most of the clinical samples were chosen and validated by the PRM strategy in a different patient cohort [34]. Detailed information of the selected glycopeptides is listed in Table S12. The additional cohort included 11 healthy controls, 12 HBV samples, 11 CIR samples and 11 HCC samples, and the clinical information of the participants is summarized in Table S6. For QC of the LC-MS/MS performance, pool samples were measured as a QC standard every 10 runs. The QC standard was analysed using the same method and conditions as the clinical samples. The pairwise Spearman's correlation coefficient values between each QC run are shown in Fig. S16. The average correlation coefficient among the QC samples was 0.98, with the maximum and minimum being 1.00 and 0.96, respectively. The absolute intensity of each of the selected glycopeptides was extracted using Skyline and is listed in Table S13. As shown in Fig. S17, we obtained similar quantitative results as before. The AUC of IgG1-H3N5F1 was 0.924, which indicated that the HC group could be easily distinguished from the HCC group (Fig. S18). In summary, our strategy was successfully applied to analyse intact *N*-glycopeptides from IgG for assessing the severity of liver diseases. We identified the largest data set of subclass-specific and site-specific *N*-glycosylation of human serum IgG reported to date. Based on these data, the different clinical stages of liver diseases could be distinguished using their glyco-signatures, providing the potential for non-invasive monitoring and pre-stratification of liver diseases.

## CONCLUSION

In this work, we developed a multiplexed, site-specific *N*-glycoproteomics quantification strategy (HTiGQs) and applied it to the discovery of biomarkers specific to liver diseases. TMT labeling was utilized for multiplexed quantification and DMEN labeling was introduced for improving the efficiency of ETD-MS/MS. We applied the sceHCD-pd-ETD mode during MS acquisition that HCD spectra can provide reporter ions in MS2 and ETD spectra play a complementary role in identification. Finally, we employed our strategy to analyse the intact *N*-glycopeptides of IgG from 90 individual human serum samples collected from patients with HBV, CIR and HCC, as well as the HC group, for biomarker discovery. A total of 313 intact *N*-glycopeptides from IgG were characterized from all samples, which was the largest data set of human serum IgG subclass-specific and site-specific *N*-glycosylation reported to date. Overall, IgG1-H3N5F1 displayed powerful prediction capability for distinguishing HCC patients from HC and two glycopeptides, IgG1-H3N5F1 and IgG4-H4N3, were identified as new potential biomarkers for classifying the severity of liver diseases in patients and healthy controls. We also revealed that the different clinical stages of liver diseases could be distinguished using the glyco-signatures. We believe this strategy would be useful for MS-based glycoproteomic biomarker discovery.

## METHODS

Detailed description is listed in Supplementary Information.

## DATA AVAILABILITY

The MS data have been deposited to the ProteomeXchange Consortium (http://proteomecentral.proteomexchange.org) [35] via the iProX partner repository with the data set identifier PXD027379.

## Supplementary Material

nwac059_Supplemental_Files
