# Supplementary material for: High-throughput site-specific N-glycoproteomics reveals glyco-signatures for liver disease diagnosis
Source: Natl Sci Rev. 2022 Apr 5;10(1):nwac059. doi: 10.1093/nsr/nwac059 (PMC9985154; doi:10.1093/nsr/nwac059)
Supplement: nwac059_Supplemental_Files [file nwac059_supplemental_files.zip › Supplementary_Information.pdf]

Supplementary Information

High-throughput site-specific N-glycoproteomics reveals glyco-signatures for liver disease diagnosis

Zhenyu Sun[a], Bin Fu[b], Guoli Wang[a], Lei Zhang[a], Ruofan, Xu[c],Ying Zhang[a,b,*], Haojie Lu[a,b,*]

[a] Shanghai Cancer Center and Institutes of Biomedical Sciences, Fudan University. Shanghai 200032 (China).

[b] Department of Chemistry and NHC Key Laboratory of Glycoconjugates Research, Fudan University. Shanghai 200032 (China).

[c] Eleanor Roosevelt College, University of California San Diego. La Jolla, CA 92093, (USA).

E-mail: ying@fudan.edu.cn; luhaojie@fudan.edu.cn .

CONTENTS

Methods 5

IgG purification 5

Protein digestion 5

Preparation of TMT and DMEN labeled peptide 6

Glycopeptides enrichment 6

Preparation of glycopeptides for PRM analysis 7

MALDI-TOF MS analysis 7

LC-MS/MS Analysis 7

LC -HCD-PRM MS Analysis 8

LC-MS data processing 9

Quality control assessment 11

Data analysis 11

Result and discussion 12

Fig. S1 The carboxylate amidation reaction between the carboxyl group of glycopeptides and the amine group of DMEN. 12

Fig. S2 MALDI-TOF mass spectra of N-glycopeptides from standard IgG. a Before derivatization b After TMT derivatization c After TMT and DMEN derivatization. Hex (H), HexNAc (N), Fuc (F), Sia (S) indicate hexose, N-acetylhexsamine, fucose and sialic acid, respectively. 13

Fig. S3 a HCD MS2 spectrum of precursor m/z = 769.1253 at 15.99 min. This spectrum was assigned to the EEQYNSTYR with N-glycan composition of H3N4F1. b ETD MS2 spectrum of precursor m/z = 769.1253 at 16.00 min was assigned to the EEQYNSTYR, which was modified by N-glycan with a composition of H3N4F1. 14

Fig. S4 a ETD MS2 spectrum of precursor m/z = 815.8963 at 19.91 min. This spectrum was assigned to the EEQFNSTYR with N-glycan composition of H3N5F1. b ETD MS2 spectrum of precursor m/z = 805.6415 at 18.71 min was assigned to the EEQFNSTYR,

which was modified by N-glycan with a composition of H3N5F1. 15

Fig. S5 The relationship between average number of identified intact N-glycopeptides and the intensity of reporter ions in ETD spectrum. 16

Fig. S6 The histogram displays quantified glycopeptide ratio distribution from an equally mixed ratio using HTiGQs. 17

Fig. S7 The distribution of standard deviation from an equally mixed ratio. 18

Fig. S8 a The distribution of charge states of N-glycopeptides identified from serum using HTiGQs. b Overlap of identified intact N-glycopeptides from human serum in HCD and ETD MS acquisition modes. c Glycan type distribution of intact N-glycopeptides identified from human serum. d Percentage of one glycosite with different numbers of N-glycans. 19

Fig. S9 SDS-PAGE-CBB (Coomassie Brilliant Blue) of IgG captured by protein G from human serum for three replicates (lane 2-3). Standard IgG was employed as reference 20

Fig. S10 Pearson correlation coefficient R^2 values for the binary comparison of the 5 quality control samples. 21

Fig. S11 The distribution of identified intact N-glycopeptides of IgG from all the samples, which were classified by subclass and glycan types. 22

Fig. S12 The distribution of the ratios of differential expressed intact N-glycopeptides. a The healthy controls vs HBV, b The healthy controls vs CIR, c The healthy controls vs HCC, d HBV vs CIR, e HBV vs HCC, f CIR vs HCC. healthy controls (A), HBV (B), CIR (C), and HCC (D). 23

Fig. S13 UpSet plot displays the overlap of differential expressed intact N-glycopeptides from healthy controls (A), HBV (B), CIR (C), and HCC (D). 24

Fig. S14 a ROC curves of IgG1-H3N4F1, which can distinguish HCC from healthy controls. b ROC curves of IgG2-H6N3F1, which can distinguish CIR and HBV. 25

Fig. S15 ROC curves of the five combination, AUC values were calculated for the classification of HBV, CIR, HCC and HC. ROC curves from a The HC and HBV, b HC and CIR, c HC and HCC, d HBV and CIR, e HBV and HCC. 26

Fig. S16 Pearson correlation coefficient R^2 values for the binary comparison of the 4 quality control samples. 27

Fig. S17 A heatmap displays the absolute intensity of differential expressed intact

N-glycopeptides among healthy controls (A), HBV (B), CIR (C), and HCC (D). 28

Fig. S18 IgG1-H3N5F1 on a C18 column with the PRM method. a IgG1-H3N5F1 was found in healthy controls. b IgG1-H3N5F1 was found in HCC samples. c The expression level change (absolute abundance) of IgG-H3N5F1 in HC, HBV, CIR and HCC samples. Asterisks indicate statistical significance based on unpaired two-sided Welch's t test. p value: *, < 0.05; **, < 0.01; ***, < 0.001. d Receiver operating characteristic (ROC) curve of IgG1-H3N5F1 and AUC value was calculated for the classification of healthy controls and HCC patients. 29

Additional spectra 30

Tables 138

Table S1 The N-glycan database of IgG. 138

Table S2 MS2 setting 142

References...143

Methods

IgG purification

IgG was isolated from human serum using Protein G (Roche). Three buffers were prepared: Buffer 1 (binding buffer) consisted of 10 mL RIPA lysis buffer (Merck) and 200 μL protease cocktail (Roche); Buffer 2 (washing buffer) was 10 mM PBS; Buffer 3 (elution buffer) consisted of 0.1% TFA (v/v, Sigma). First, the protein G-agarose (40 μL) were wash with 200 μL binding buffer twice. Second, 10 μL of serum were diluted with 200 μL binding buffer and then diluted samples were added into protein G-agarose and incubated for 4 hours with moderate agitation at 4 ℃. Third, the samples were centrifuged for 5 minutes at 5000 rcf and invalid portion was wiped off. Fourth, the samples were washed by 200 μL binding buffer twice and washing buffer twice to thoroughly wash away all unbound non-IgG protein components. Fifth, 100 μL elution buffer was added and incubated for 5 minutes with moderate agitation at 4 ℃. Finally, the sample was centrifuged and collected. This step was repeated three times. The purity of eluted IgG was further validated by SDS-PAGE and the protein concentration was quantified using Pierce BCA Protein Assay Kit (Thermo Scientific) and 100 μg of each sample were used for further analysis. At the same time, 2 μg proteins from each sample were pooled as the internal reference. The IgG was stored at -20 ℃ until digestion.

Protein digestion

For IgG proteins, first, IgG was dissolved in 100 mM triethylammonium bicarbonate (TEAB, Sigma) buffer at 1 μg/μL. Second, the proteins were reduced with 10 mM dithiothreitol (DTT, Sigma) for 30 min at 56 ℃ and subsequently alkylated with 20 mM iodoacetamide (IAA, Sigma) for 30 min at room temperature in the dark. Third, the proteins were digested overnight at 37 ℃ with trypsin (Beijing Shengxia Proteins Scientific Ltd) at ratio of 1:50 (w/w). For serum protein digestion, 10 μL of serum was diluted with 190 μL lysate contained of 8M Urea (Sigma) in 100 mM Tris-HCl

(pH = 8.0), and then proteins were reduced and alkylated as described above. After alkylation reaction, samples were treated using FASP with minor modification. First, samples were added to the membrane and centrifuged for 40 min at 14,000 g. The membrane was washed by 200 μL 10 mM PBS to remove urea and excess DTT and IAA for three times at 14,000 g. Then reverse centrifugation was performed to recover the proteins at 1000 rcf for 10 min. The protein concentration was determined using Pierce BCA Protein Assay Kit. Then, Glu-C was added at ratio of 1:50 (w/w), and the proteolysis was proceeded for 12 h at 37 ℃. Trypsin was added at ratio of 1:50 (w/w) subsequently, and proceeded for 16 h at 37 ℃. After digestion, peptides were desalted using a C18 SPE cartridge and subsequently lyophilized in vacuum. In addition, the internal reference was divided into 10 equal parts.

Preparation of TMT and DMEN labeled peptide

TMT labeling procedure (Thermo Scientific) was performed according to the manufacturer's instruction. And then, peptides were desalted using HILIC enrichment and subsequently lyophilized in vacuum. The glycopeptides were labeled with DMEN according to our previous work. [1] And then, the labeled glycopeptides were enriched using HILIC. The internal reference was labeled with TMT-131, which was used to normalize batch effects. To eliminate variation caused by different runs or instruments, two samples from each of four groups were selected randomly and were labeled with the other 9 channels (healthy controls were labeled with TMT-126, TMT-127C; HBV samples were labeled with TMT-127N, TMT-128C; CIR samples were labeled with TMT-128N, TMT-129C; HCC samples were labeled with TMT-129N, TMT-130C; random samples were labeled with TMT-130N).

Glycopeptides enrichment

Samples were acidified by adding 10% (v/v) trifluoroacetic acid (TFA, Sigma) to a final concentration of 1%, and then the loading buffer consisting of 80% (v/v) acetonitrile (ACN, Sigma) and 1% TFA was added to samples. The enrichment was

performed according to previous literature [1].

Preparation of glycopeptides for PRM analysis

IgG was isolated from 10 μL human serum using the methods described in the IgG purification. The protein concentration was quantified using Pierce BCA Protein Assay Kit (Thermo Scientific) and 100 μg of each sample were used for further analysis. Then proteins were reduced, alkylated and digested as described above. Peptides were desalted using a C18 SPE cartridge and subsequently lyophilized in vacuum. And then, the glycopeptides were enriched as described above. The glycopeptides of IgG were analyzed by LC-HCD-PRM MS.

MALDI-TOF MS analysis

One microliter of sample and one microliter of CHCA matrix solution (5 mg/mL in 50% ACN containing 0.1% TFA) were spotted on the MALDI plate for MS analysis. The MALDI-MS spectra were acquired using 5800 Proteomics Analyzer (Applied Biosystems, Framingham, MA, USA) equipped with a Nd: YAG laser (355 nm), an acceleration voltage of 20 kV and a repetition rate of 400 Hz. The spectrometer was operated in positive reflection mode accumulated by 1000 laser shots. The glycopeptide containing amidated sialic acid was detected as [M + H]+ at m/z 3637.71 after labeling, demonstrating that the instable sialic acids were protected after labeling (Fig. S2).

LC-MS/MS Analysis

Unlabeled, TMT-labeled and TMT-DMEN labeled N-glycopeptides were resuspended with 0.1% FA, solvent A (water with 0.1% formic acid), separated by nano LC, and analyzed by online electrospray tandem mass spectrometry. The experiments were performed on an EASYnLC 1000 system (Thermo Fisher Scientific) connected to an Orbitrap Fusion mass spectrometer (Thermo Fisher Scientific) equipped with an online nano electrospray ion source with 2.3 kV of spray potential. Glycopeptides

were loaded onto the analytical column (Acclaim PepMap C18, 75 μm×25 cm) and subsequently separated with a linear gradient, Solvent A was 0.1% formic acid in water. Solvent B was 80% acetonitrile with 0.1% formic acid. The gradient was 1 h in total for the glycopeptide from IgG samples: 0-20% from 0 to 50 min, 20-100% from 50 min to 55 min, and held for 100% for the last 5 min. The gradient was 4 h in total for the glycopeptide from serum samples: 0-15% from 0 to 165 min, 15-45% from 166 min to 172 min, 45-90% from 172 min to 173 min, held for 90% for the last 3 min, 90-1% from 176 to 176 min 10 s, and held for 1% for the last 3 min 50 s. The column flow rate was maintained at 200 nL/min. The parameters for glycopeptides analysis were set as following. MS1: (1) scan range (m/z) = 350-2000; resolution = 30,000; AGC target = 1,000,000; maximum injection time = 50 ms; included charge state = 2-8; dynamic exclusion after n times, n = 1; dynamic exclusion duration = 15 s; each selected precursor was subject to one HCD-MS/MS; (2) HCD-MS/MS: isolation window = 1.8; detector type = Orbitrap; resolution = 60,000; AGC target = 500,000; maximum injection time = 250 ms; collision energy = 50%; stepped collision mode on, energy difference of ± 10% (10% as absolute value in the Orbitrap Fusion). If peaks at m/z 204.0867, 138.0545, 186.0714, or 168.0661 (±m/z 0.05) were within the top 2 most abundant peaks, a supplemental activation ETD MS/MS scan of the precursor ion in the linear ion trap was triggered [2]. Other ETD parameters were default settings. For the evaluation of the capacity of the N-glycopeptide identification and the intensity of TMT reporter ions, we evaluated six HCD collision energy and ETD parameters. We summarized these parameters in Table S1. Other parameters were set the same as before.

LC -HCD-PRM MS Analysis

N-glycopeptides from IgG samples were resuspended with 0.1% FA, solvent A (water with 0.1% formic acid), separated by nano LC, and analyzed by online electrospray tandem mass spectrometry. The experiments were performed on an EASY-NanoLC 1200 system (Thermo Fisher Scientific) connected to an Orbitrap Exploris480 mass

spectrometer (Thermo Fisher Scientific) equipped with an online nano electrospray ion source with custom spray potential. Glycopeptides were loaded onto the analytical column (Acclaim PepMap C18, 75 μm×25 cm) and subsequently separated with a linear gradient, Solvent A was 0.1% formic acid in water. Solvent B was 80% acetonitrile with 0.1% formic acid. The gradient was 41 min in total for the glycopeptides from IgG samples: 2-5% from 0 to 1 min, 5-20% from 1 min to 31 min, 20-95% from 31 min to 37 min, and held for 95% for the last 5 min. The column flow rate was maintained at 250 nL/min. The parameters for glycopeptides analysis were set as following. MS1: (1) scan range (m/z) = 400-2000; resolution = 60,000; Normalized AGC Target (%)= 300; maximum injection time = 50 ms; included charge state = 2-6; dynamic exclusion after n times, n = 1; dynamic exclusion duration = 15 s; each selected precursor was subject to one HCD-MS/MS; (2) HCD-MS/MS: isolation window = 2.2; detector type = Orbitrap; resolution = 15,000; Normalized AGC Target (%)= 300; maximum injection time = 50 ms; collision energy (%) = 20,30,40.

LC-MS data processing

XCalibur 3.0 was used for data processing. Raw data was used directly without any further processing for N-glycopeptide identification using Byonic (Protein Metrics, San Carlos, CA). The following parameters were used for IgG search: Trypsin was selected as enzyme. The search was performed using the following parameters: (1) fixed modification, carbamidomethylamidation of C; (2) dynamic modifications, oxidation of M, and DMEN modifications of D, E and C-terminal of peptides; TMT modifications of K and N-terminal; (3) maximum missed cleavages, 0; (4) precursor ion mass tolerance 10 ppm; (5) fragment ion mass tolerance 20 ppm. The IgG glycan database was summarized from previous reports [3]. The detail information was listed in Table S1. The protein databases contain all four IgG subclasses: IgG1 (Uniprot ID, P01857, IGHG1), IgG2 (Uniprot ID, P01859, IGHG2), IgG3 (Uniprot ID, P01860, IGHG3), IgG4 (Uniprot ID, P01861, IGHG4). To be noted, the glycopeptide of IgG3

in some samples is actually the same as that of IgG2 due to a polymorphic variant frequent in different population [4]. EEQY*NSTFR (IgG3) may become EEQF*NSTFR (IgG2) because SNP (rs12890621) in human IgG3 heavy chain affected glycopeptide sequence. For serum sample, Trypsin and Glu-C were selected as enzyme and maximum missed cleavage was set to 2. The other parameters were the same as above. A human N-glycan database (from Byonic database) containing 182 human N-glycans was employed. The *Homo sapiens* proteome database was downloaded from UniProtKB on 15 Jul. 2019, containing 20431 reviewed protein sequences. In addition, The N-glycopeptides identification were filtered to 1% FDR and Byonic score ≥150 [5,6].

For quantification of intact N-glycopeptides, the intensity of TMT reporter ions was extracted as follow: (1) HCD and ETD spectrum were separated using via MSConvert. (2) For HCD spectrum, the TMT reporter ions was extracted directly. For ETD spectrum, paired HCD spectrum were located through the scan number, and then the TMT reporter ions were extracted from the paired HCD spectrum. The code is available on https://github.com/fubin1999/lab/blob/main/reporter_ions.py.

Skyline software (version 21.1) was used for quantification of selected glycopeptides. Skyline analysis requires two types of settings, peptide settings and transition settings, to generate a spectral library which is applied to patient samples for quantification. In peptide settings, a background library was generated by uploading raw data, the peptide sequence, and a list of identified glycopeptides which were identified from the raw data by Byonic. All possible glycan side chains were added as structural modifications. In transition settings, ion types were set as b, y, and special ions where oxonium ion 138.0550, 204.0866, 366.1395 and Y1 ions (peptide + HexNAc) were added. Skyline is then ready for importing the raw data of patients for quantitative analysis. Integration of all transitions was automatically calculated and could be exported as a report after manual checking.

Quality control assessment

For the quality control, the Hela cell lysate was measured from every 5 runs as the quality-control (QC) standard. All the pairwise Spearman's correlations were calculated using corrplot (version 0.88) package in R and the coefficient of determination were higher than 0.97 (Fig.S10). For PRM analysis, 1 μL sample from each sample were pooled as the internal reference. The internal reference was measured from every 10 runs as the QC standard. All the pairwise Spearman's correlations were also calculated using corrplot (version 0.88) package in R and the coefficient of determination were higher than 0.96 (Fig. S16).

Data analysis

Sequons analysis for identified glycopeptides was using the ggseqlogo (version 0.1) package in R. Gene ontology enrichment analysis was performed using the clusterProfile (version 3.16.0) package in R. The protein-glycan network was created in cytoscape [7]. The glycan co-occurrence networks were created using the ComplexHeatmap (version 2.0.0) package of R. Mfuzz cluster analysis was using the Mfuzz (version 2.44.0) package of R [8]. PLS-DA plot was created using the FactoMineR (version 2.4) package of R [9].

Boxplots and histograms were created using OriginPro 2020 (9.7.0.185). In the boxplots, centerlines and squares in plotted boxed indicate the median and mean, respectively. The upper and lower ends of box show the 75th and 25th percentiles. Statistical analyses were using Microsoft excel (2019) and IBM SPSS Statistics 20. ROC curves were created using IBM SPSS Statistics 20.

Result and discussion

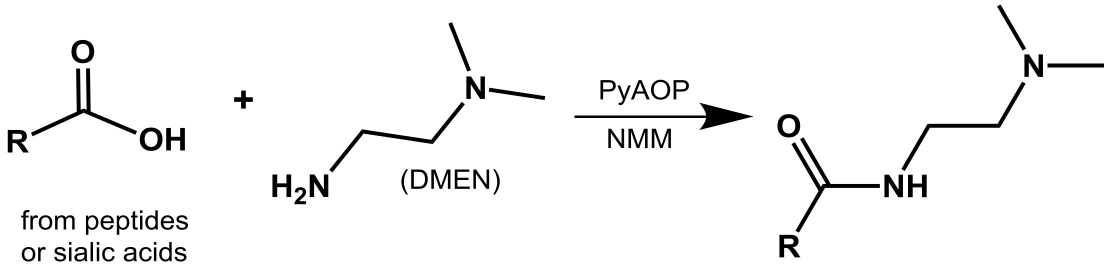

Fig. S1 The carboxylate amidation reaction between the carboxyl group of glycopeptides and the amine group of DMEN.

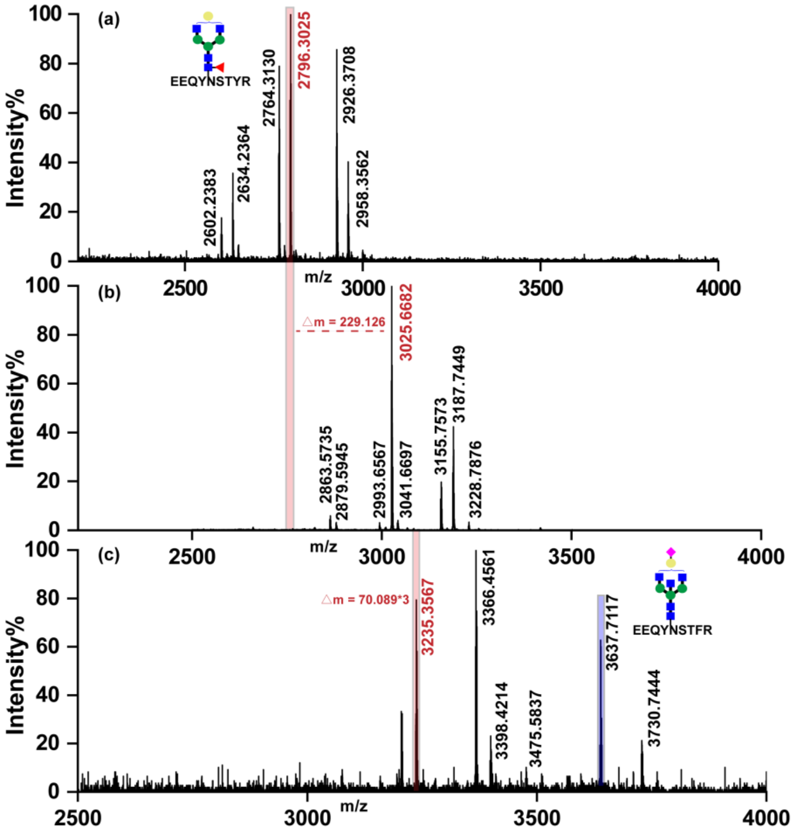

Fig. S2 MALDI-TOF mass spectra of N-glycopeptides from standard IgG. **a** Before derivatization **b** After TMT derivatization **c** After TMT and DMEN derivatization. Hex (H), HexNAc (N), Fuc (F), Sia (S) indicate hexose, N-acetylhexsamine, fucose and sialic acid, respectively.

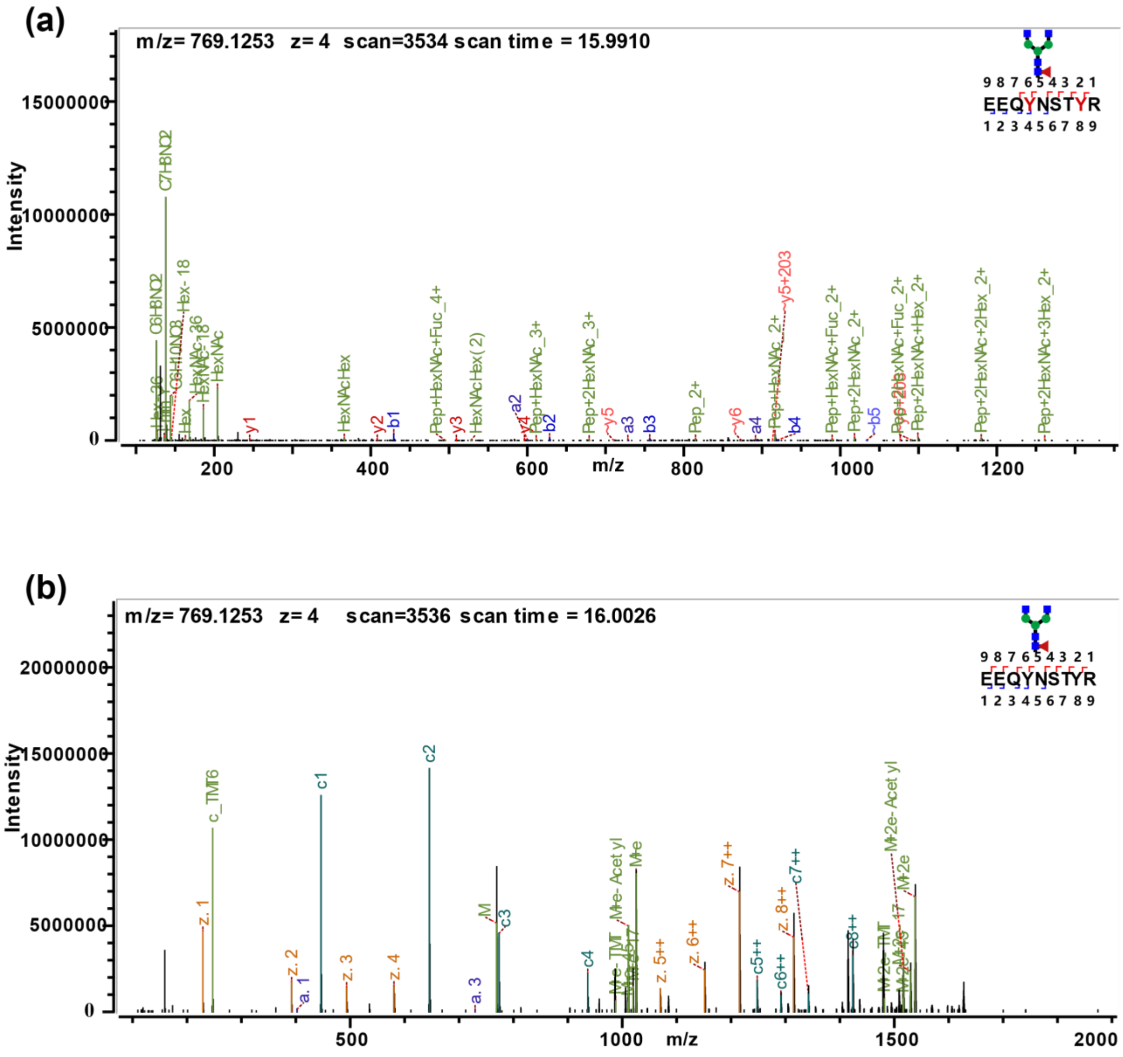

Fig. S3 **a** HCD MS2 spectrum of precursor m/z = 769.1253 at 15.99 min. This spectrum was assigned to the EEQYNSTYR with N-glycan composition of H3N4F1. **b** ETD MS2 spectrum of precursor m/z = 769.1253 at 16.00 min was assigned to the EEQYNSTYR, which was modified by N-glycan with a composition of H3N4F1.

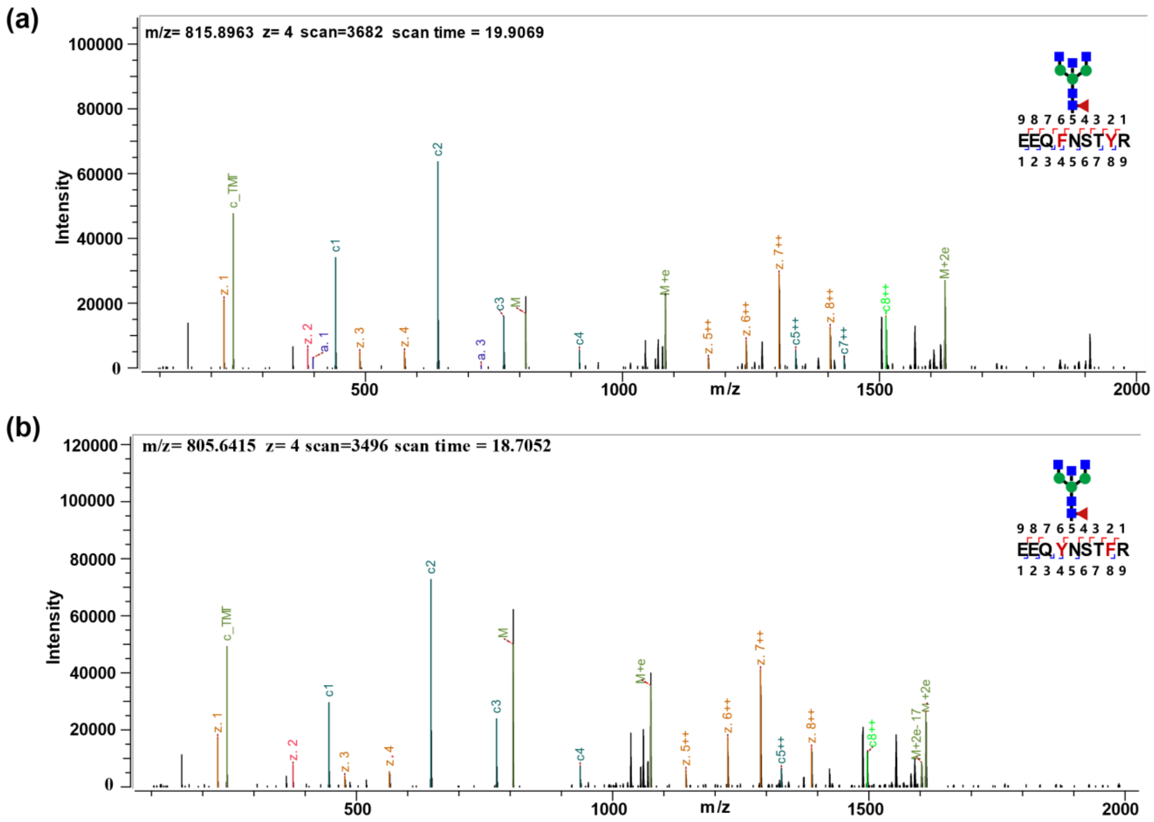

Fig. S4 a ETD MS2 spectrum of precursor m/z = 815.8963 at 19.91 min. This spectrum was assigned to the EEQFNSTYR with N-glycan composition of H3N5F1. **b** ETD MS2 spectrum of precursor m/z = 805.6415 at 18.71 min was assigned to the EEQYNSTFR, which was modified by N-glycan with a composition of H3N5F1.

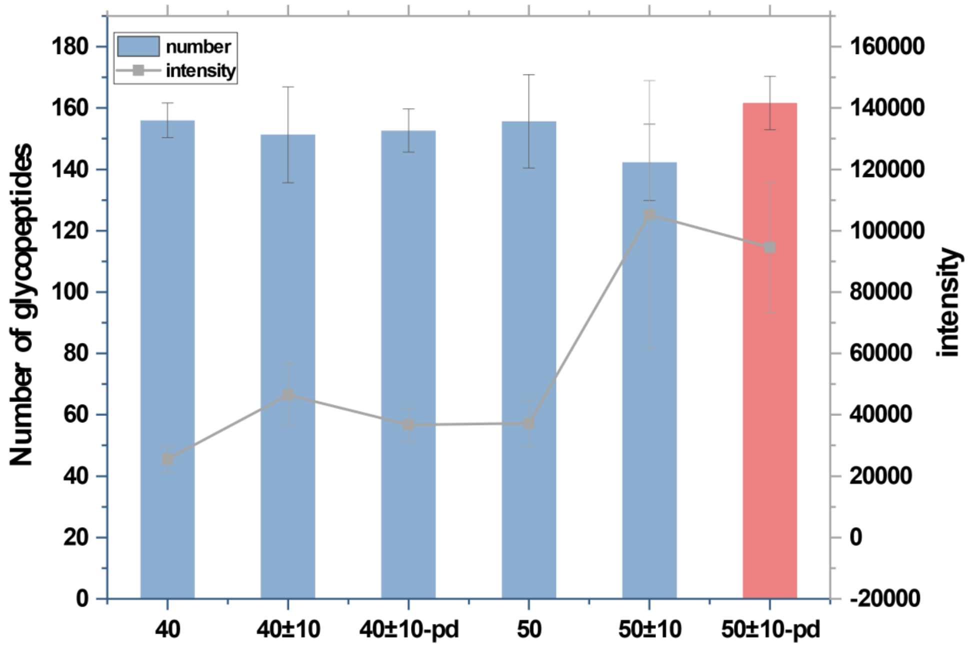

Fig. S5 The relationship between average number of identified intact N-glycopeptides and the intensity of reporter ions in ETD spectrum.

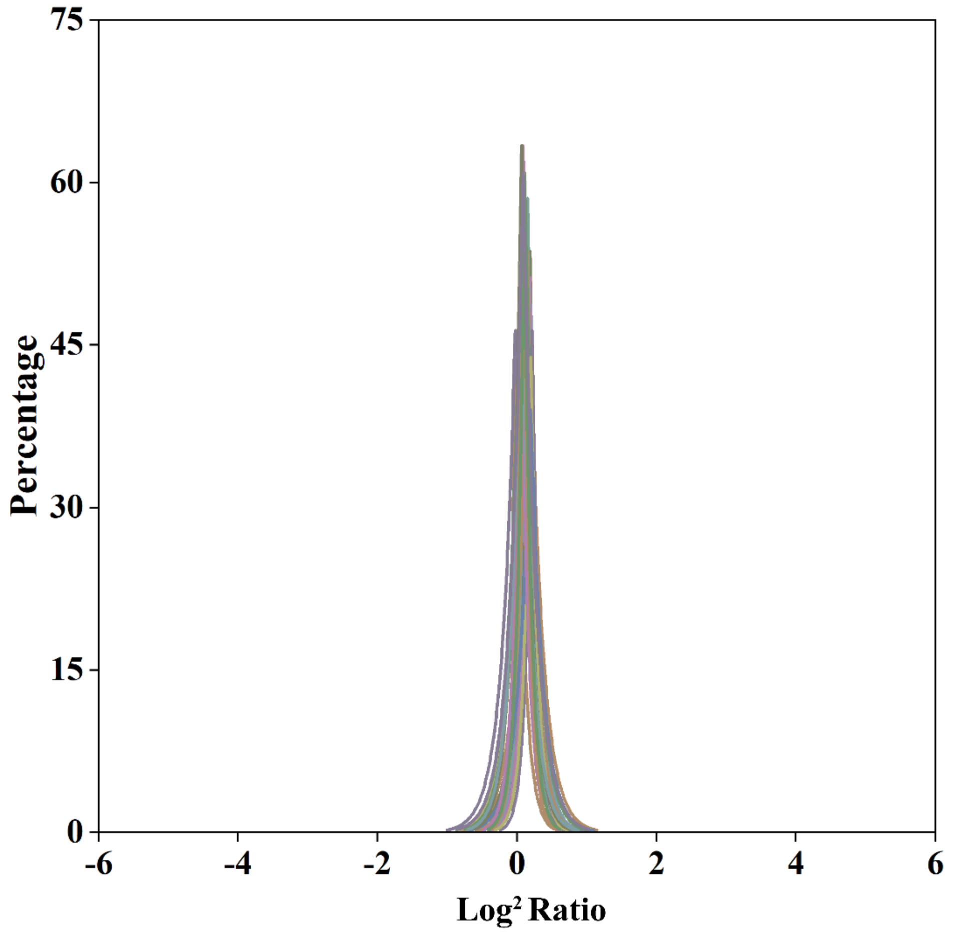

Fig. S6 The histogram displays quantified glycopeptide ratio distribution from an equally mixed ratio using HTiGQs. Twenty-seven different colored lines represent TMT channels from three replicates.

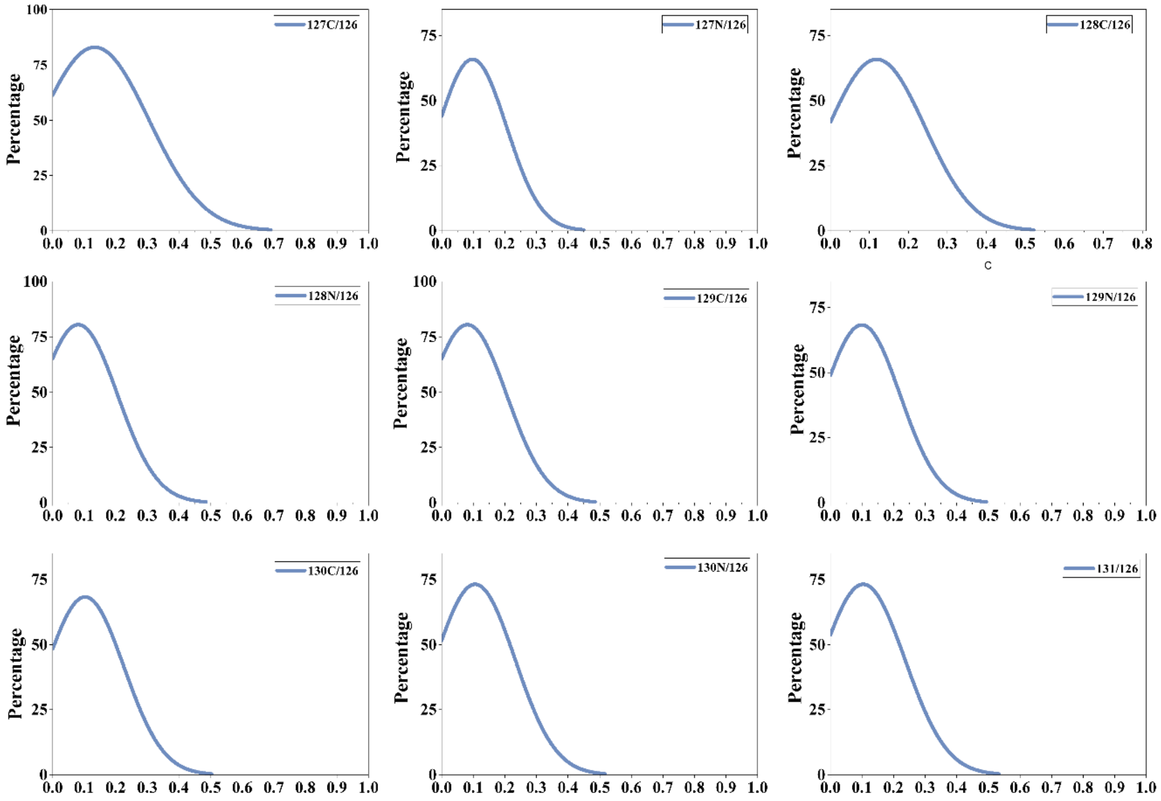

Fig. S7 The distribution of standard deviation from an equally mixed ratio.

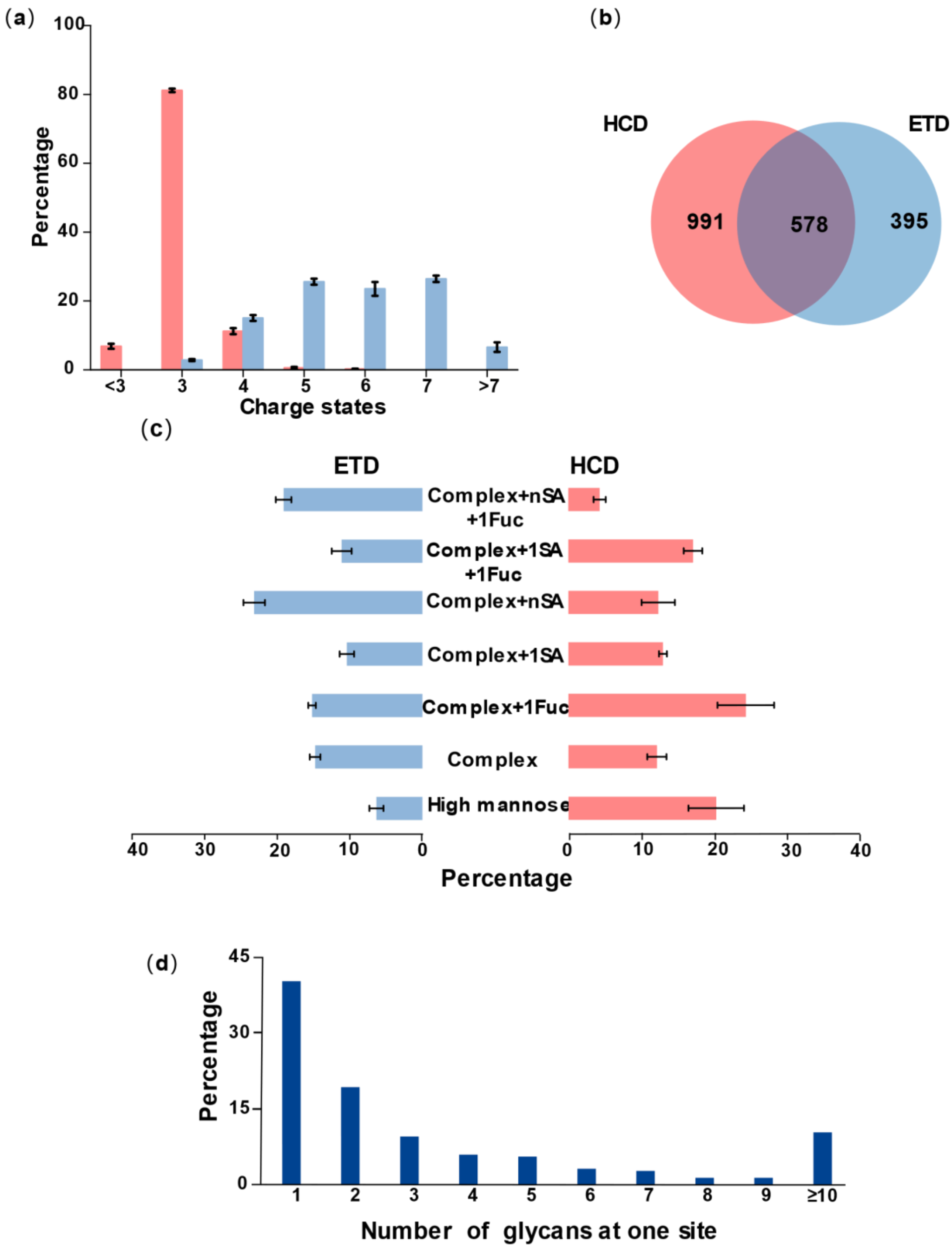

Fig. S8 **a** The distribution of charge states of N-glycopeptides identified from serum using HTiGQs. **b** Overlap of identified intact N-glycopeptides from human serum in HCD and ETD MS acquisition modes. **c** Glycan type distribution of intact N-glycopeptides identified from human serum. **d** Percentage of one glycosite with different numbers of N-glycans.

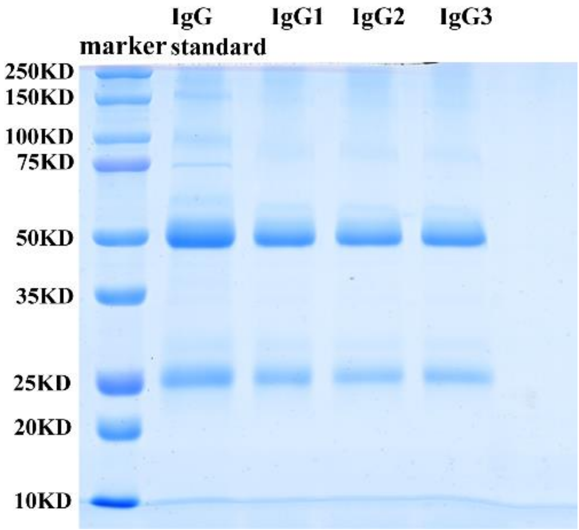

Fig. S9 SDS-PAGE-CBB (Coomassie Brilliant Blue) of IgG captured by protein G from human serum for three replicates (lane 3-5). Standard IgG was employed as reference.

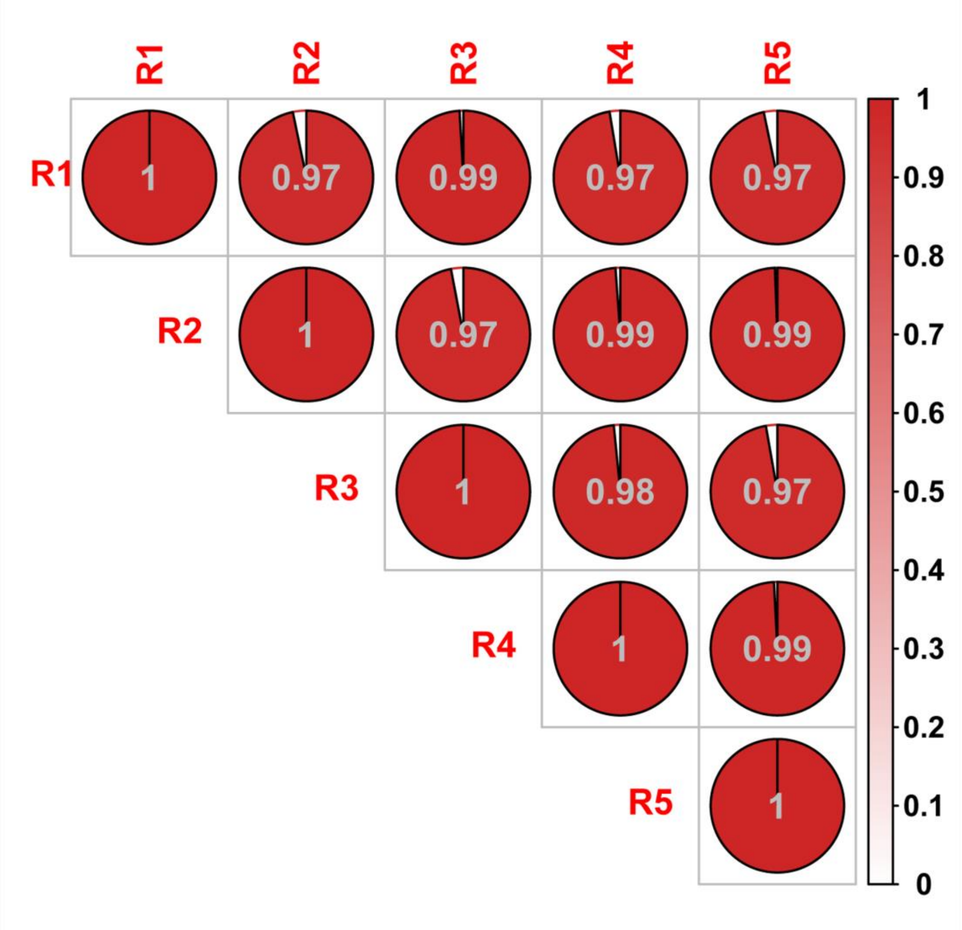

Fig. S10 Pearson correlation coefficient R^2 values for the binary comparison of the 5 quality control samples.

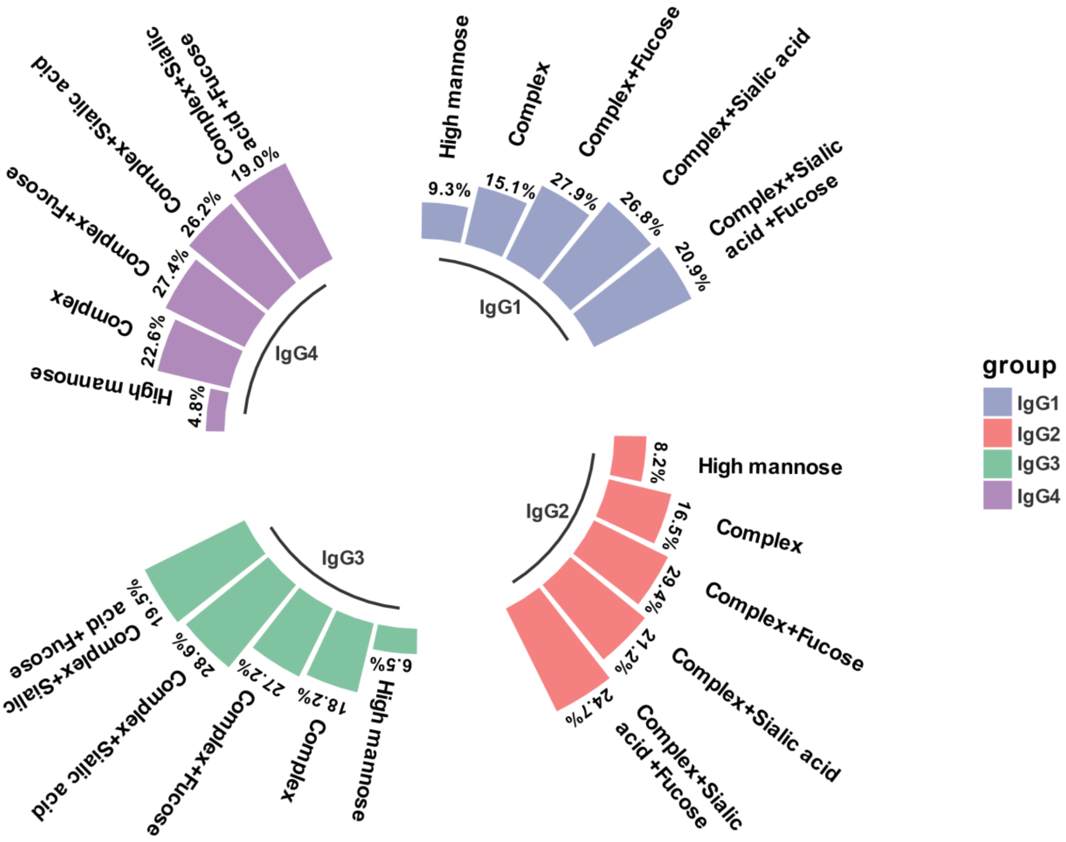

Fig. S11 The distribution of identified intact N-glycopeptides of IgG from all the samples, which were classified by subclass and glycan types.

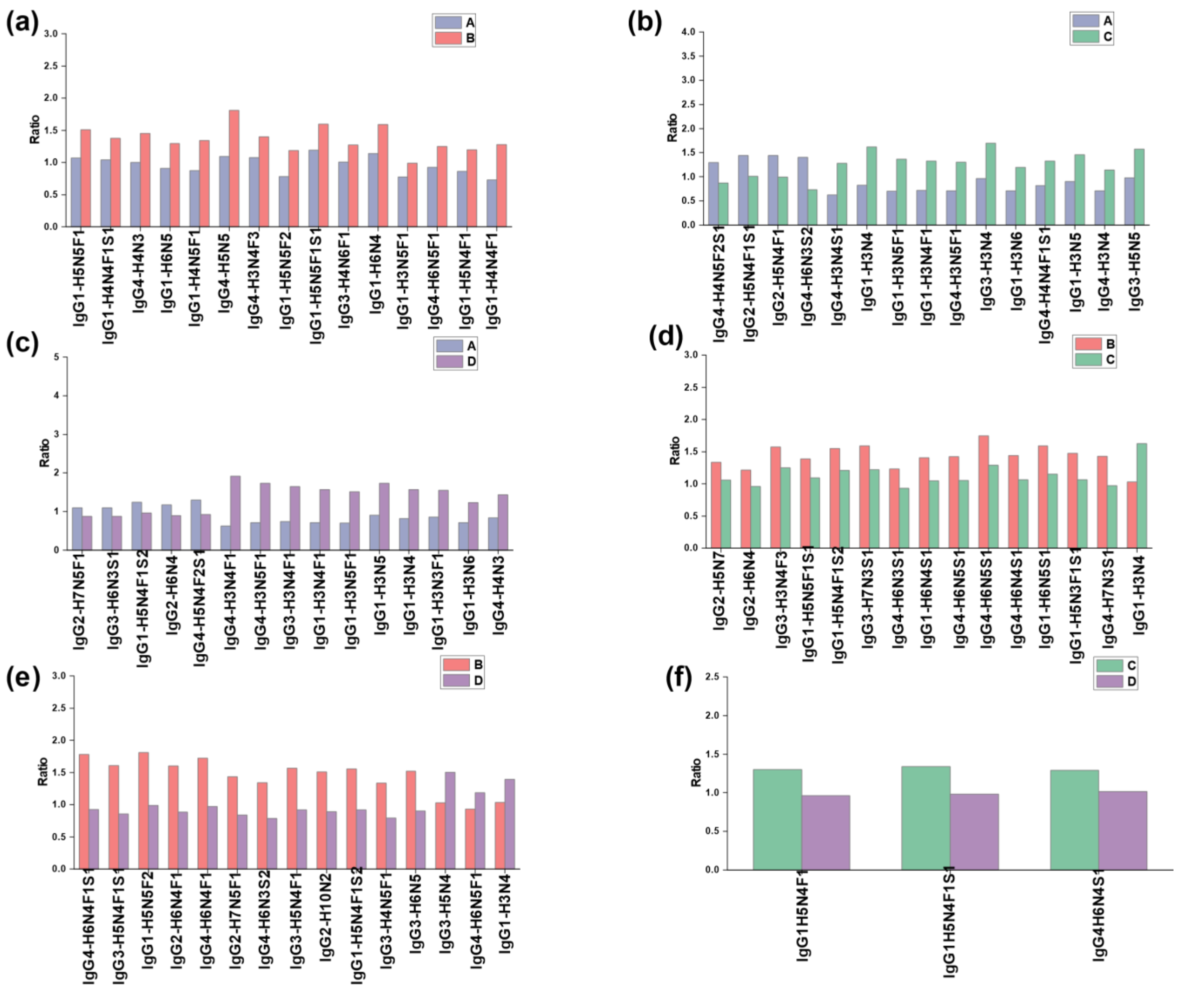

Fig. S12 The distribution of the ratios of differential expressed intact N-glycopeptides. **a** The healthy controls vs HBV, **b** The healthy controls vs CIR, **c** The healthy controls vs HCC, **d** HBV vs CIR, **e** HBV vs HCC, **f** CIR vs HCC. healthy controls (A), HBV (B), CIR (C), and HCC (D)**.**

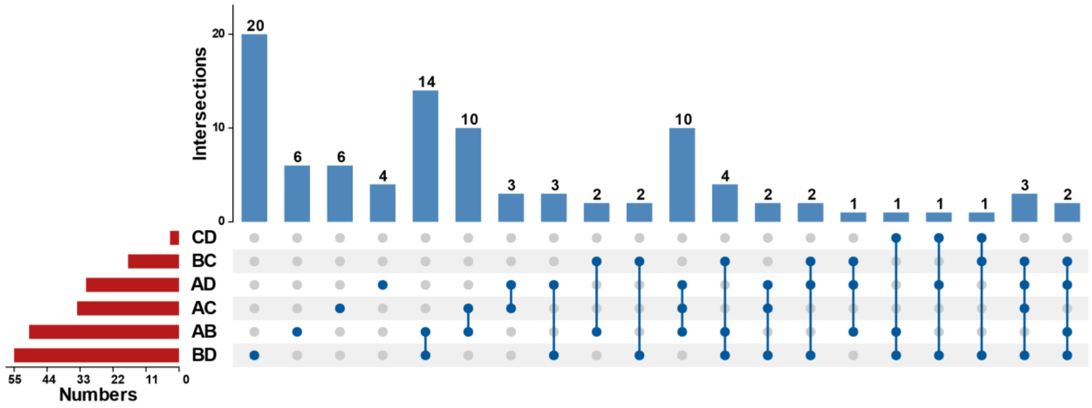

Fig. S13 UpSet plot displays the overlap of differential expressed intact N-glycopeptides from healthy controls (A), HBV (B), CIR (C), and HCC (D).

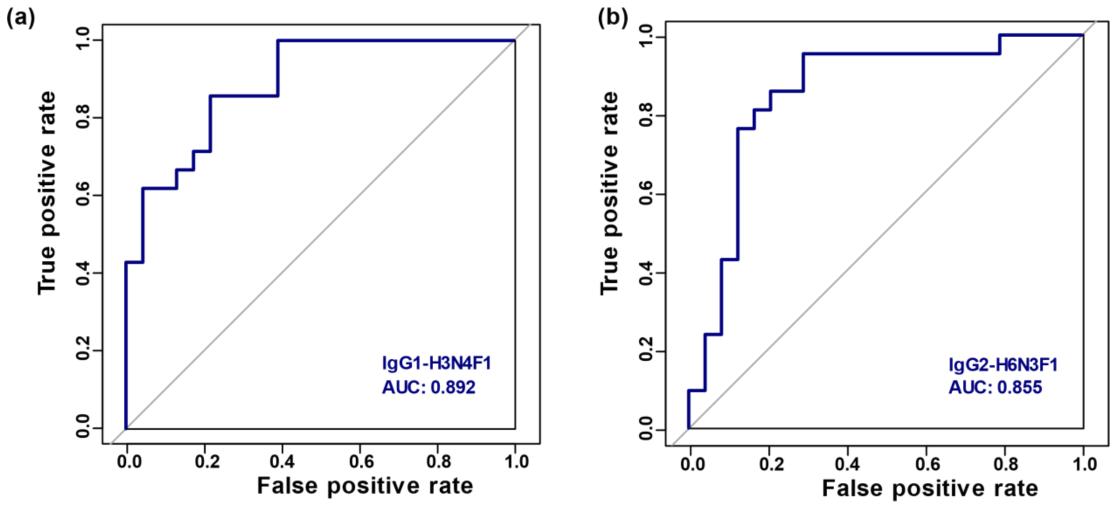

Fig. S14 a ROC curves of IgG1-H3N4F1, which can distinguish HCC from healthy controls. **b** ROC curves of IgG2-H6N3F1, which can distinguish CIR and HBV.

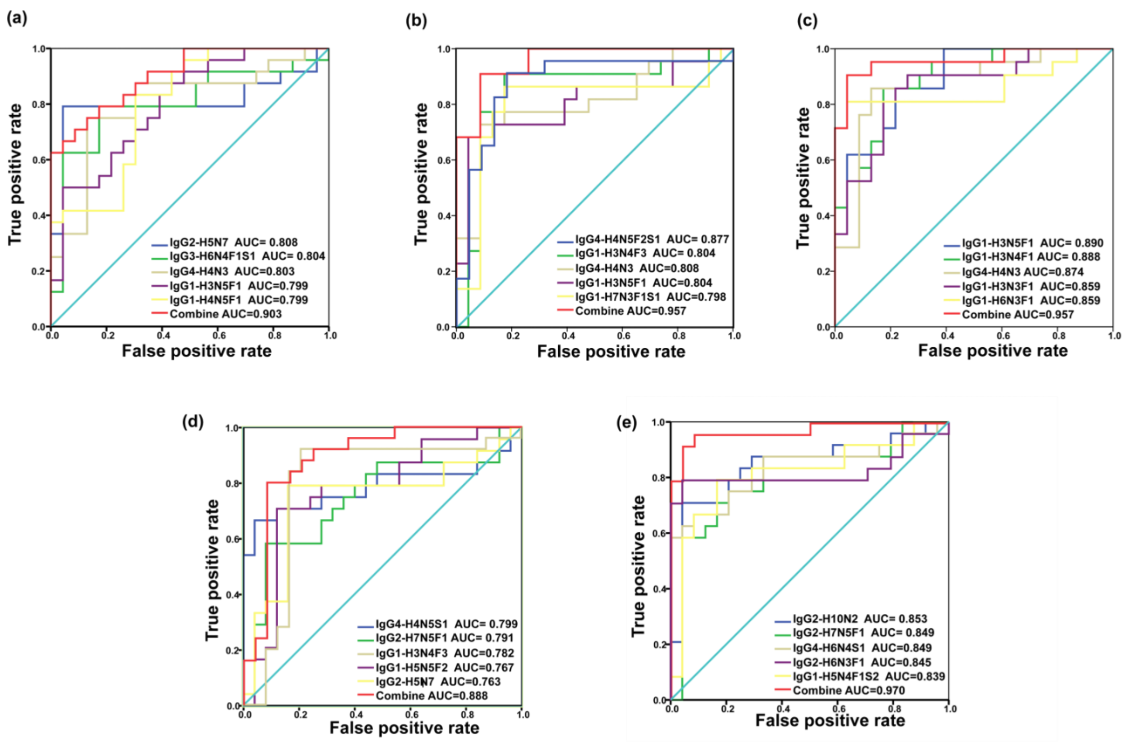

Fig. S15 ROC curves of the five combination, AUC values were calculated for the classification of HBV, CIR, HCC and HC. ROC curves from **a** The HC and HBV, **b** HC and CIR, **c** HC and HCC, **d** HBV and CIR, **e** HBV and HCC.

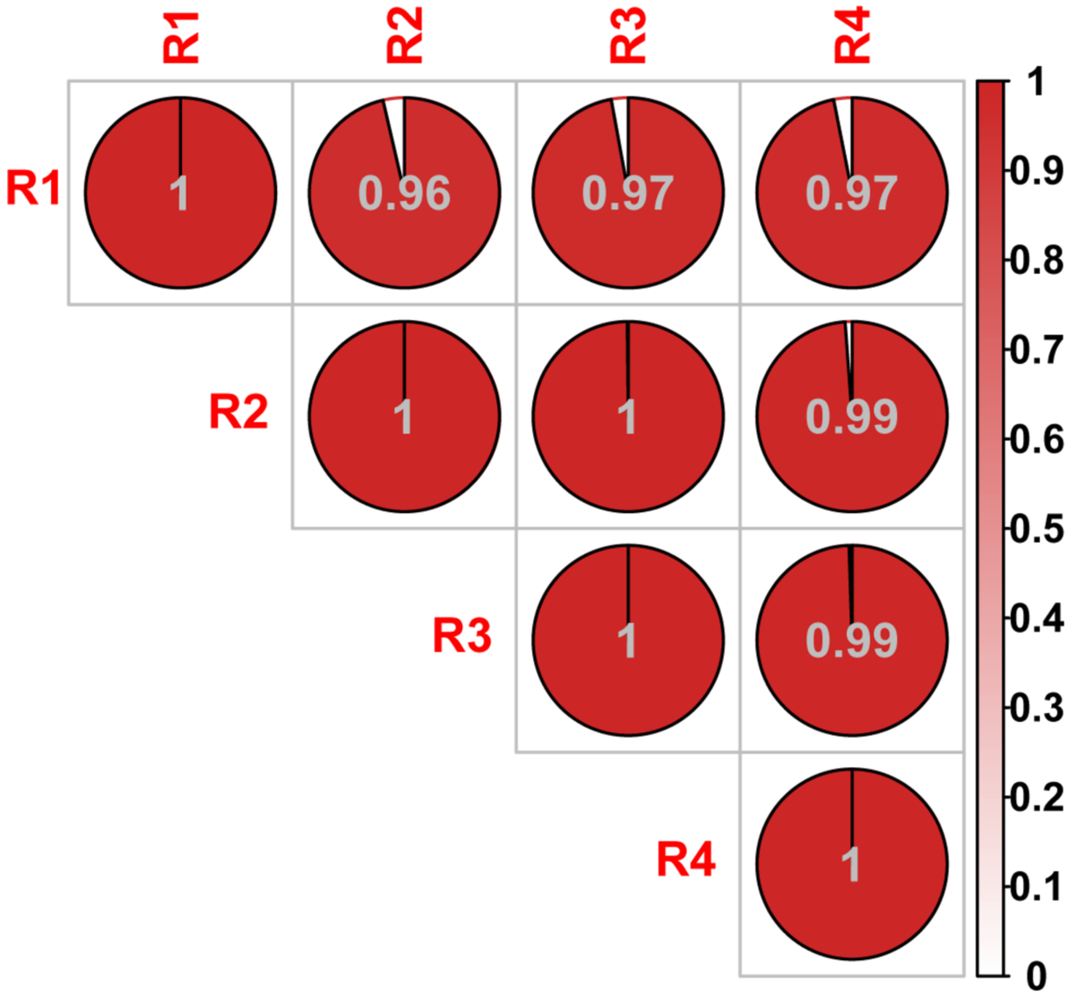

Fig. S16 Pearson correlation coefficient R^2 values for the binary comparison of the 4 quality control samples.

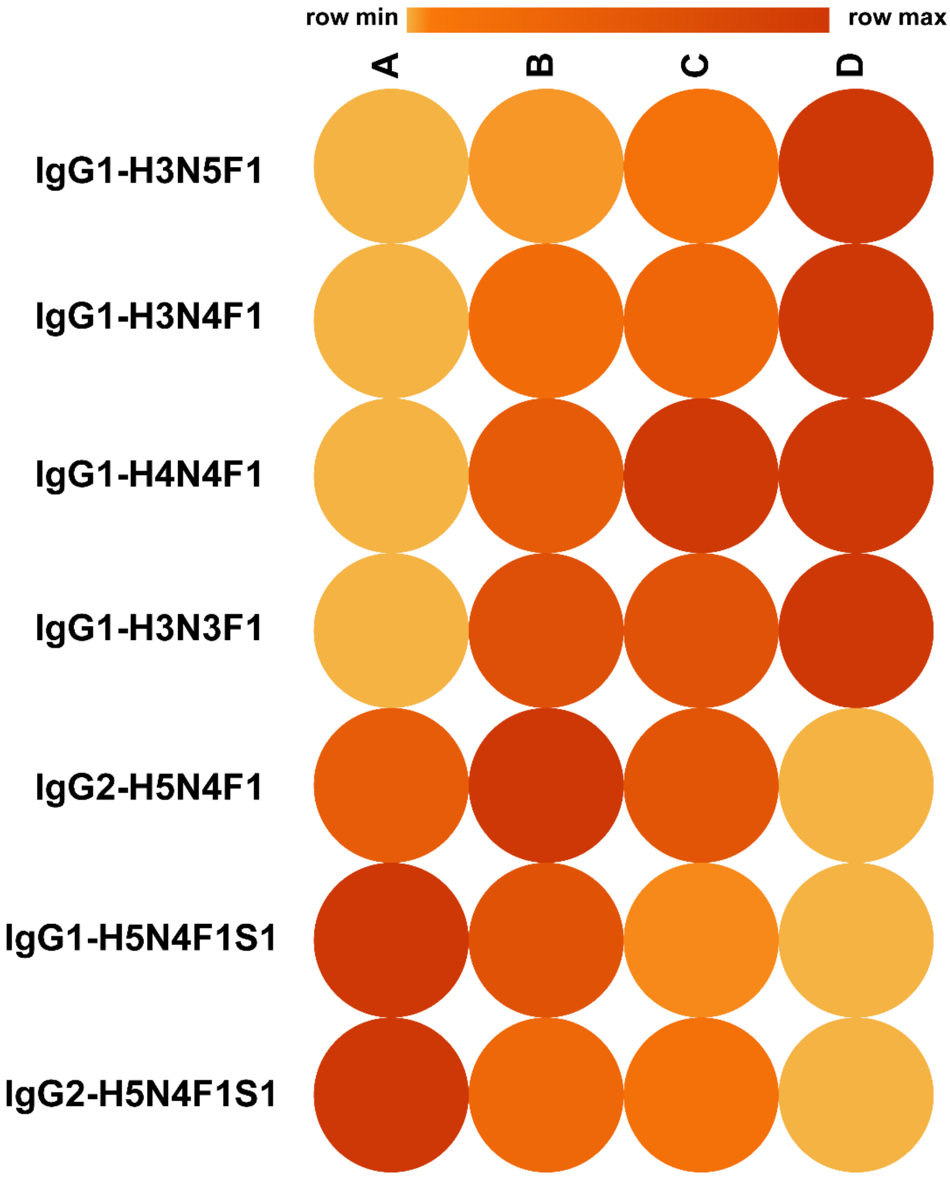

Fig. S17 A heatmap displays the absolute intensity of differential expressed intact N-glycopeptides among healthy controls (A), HBV (B), CIR (C), and HCC (D).

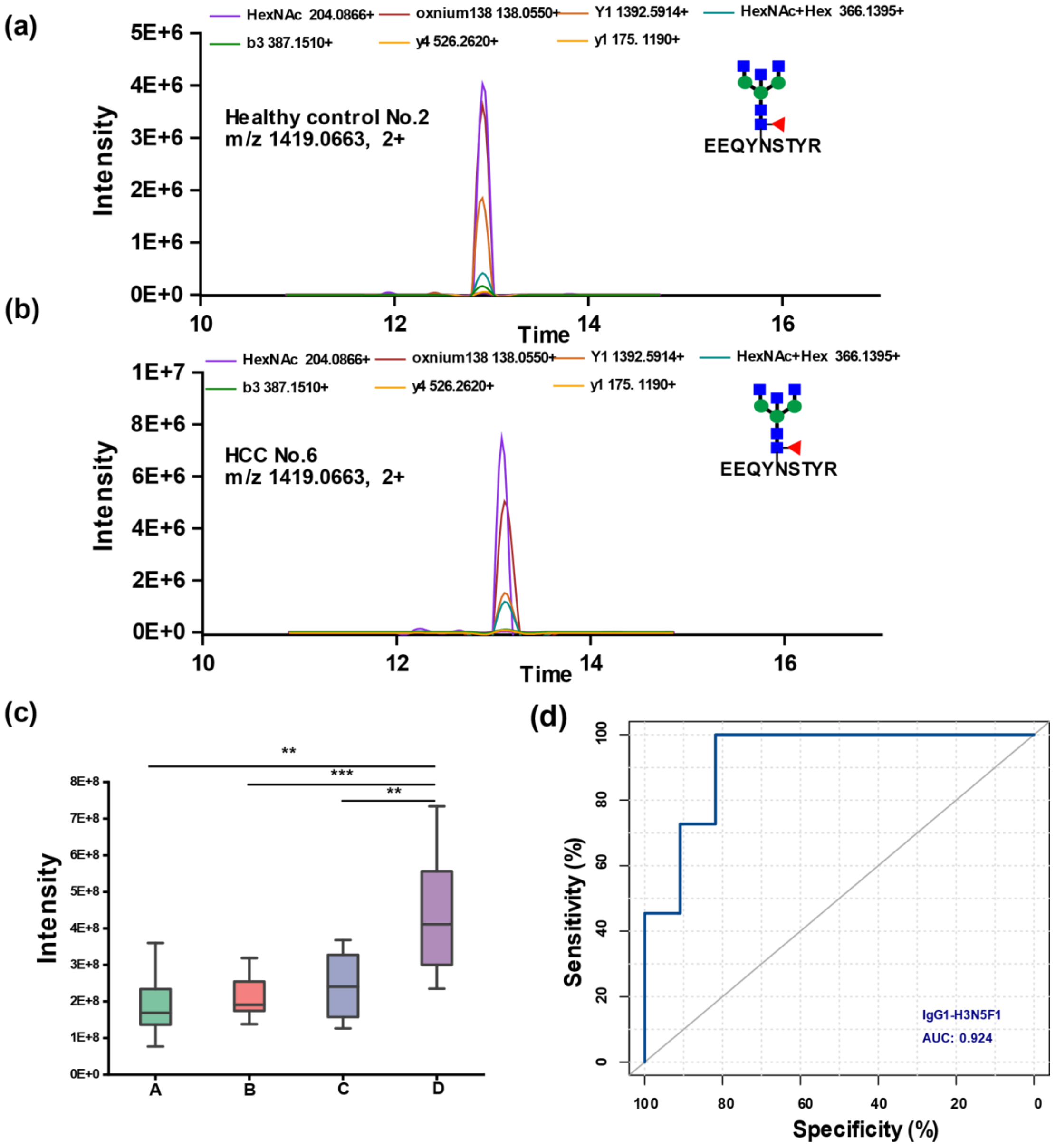

Fig. S18 IgG1-H3N5F1 on a C18 column with the PRM method. **a** IgG1-H3N5F1 was found in healthy controls. **b** IgG1-H3N5F1 was found in HCC samples. **c** The expression level change (absolute abundance) of IgG-H3N5F1 in HC, HBV, CIR and HCC samples. Asterisks indicate statistical significance based on unpaired two-sided Welch's t test. p value: *, < 0.05; **, < 0.01; ***, < 0.001. **d** Receiver operating characteristic (ROC) curve of IgG1-H3N5F1 and AUC value was calculated for the classification of healthy controls and HCC patients.

Additional spectra

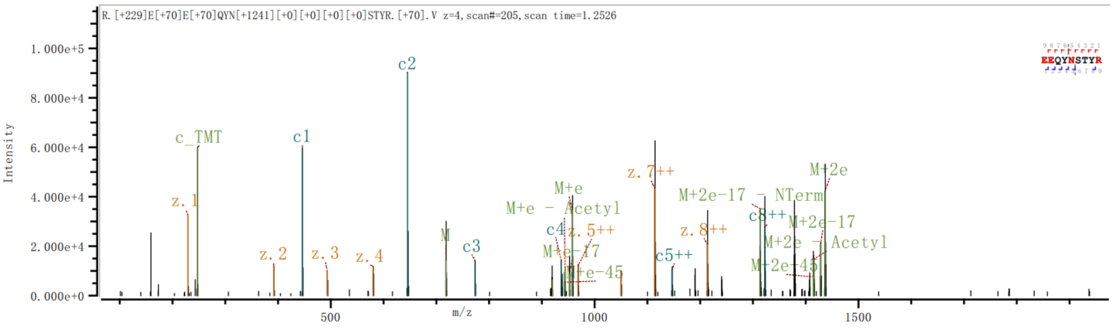

10: EEQYNSTYR

#	a. calc.	c calc.	c++ calc.	Seq.	y calc.	z. calc.	z.++ calc.	#
1	402.3210	**446.3347**	223.6710	E				9
2	601.4526	**645.4663**	323.2368	E	2442.1019	2426.0832	**1213.5452**	8
3	729.5112	**773.5248**	387.2661	Q	2242.9703	2226.9516	**1113.9794**	7
4	892.5745	**936.5882**	468.7977	Y	2114.9118	2098.8930	**1049.9502**	6
5	2248.0720	2292.0856	1146.5464	N	1951.8484	1935.8297	968.4185	5
6	2335.1040	2379.1176	1190.0625	S	596.3510	**580.3323**		4
7	2436.1517	2480.1653	**1240.5863**	T	509.3190	**493.3002**		3
8	2599.2150	2643.2286	**1322.1180**	Y	408.2713	**392.2526**		2
9				R	245.2080	**229.1892**		1

ETD MS2 spectrum of precursor m/z = 718.3570, z = 4^+ at 1.2526 min. This spectrum was assigned to the EEQYNSTYR with N-glycan composition of H3N3F1.

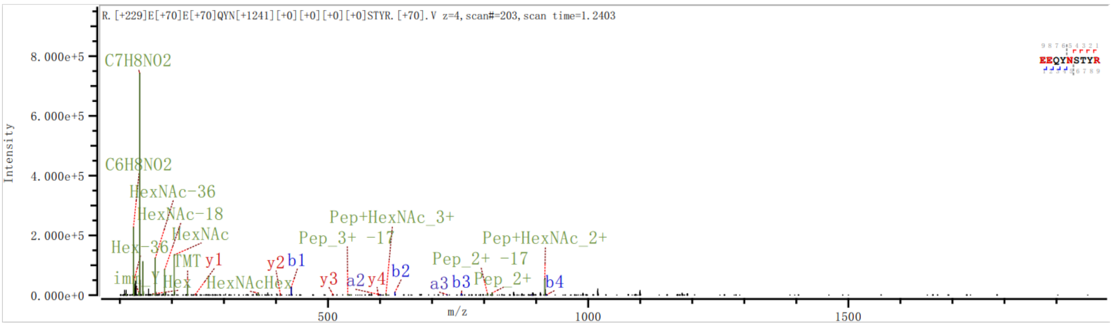

2259: EEQYNSTYR

#	a calc.	b calc.	b-18 calc.	b++ calc.	Seq.	y calc.	y++ calc.	y_3+ calc.	#
1	401.3132	**429.3081**	411.2976	215.1577	E				9
2	600.4448	**628.4397**	610.4291	314.7235	E	2442.1019	1221.5546	814.7055	8
3	**728.5034**	**756.4983**	738.4877	378.7528	Q	2242.9703	1121.9888	748.3283	7
4	891.5667	919.5616	901.5511	460.2844	Y	2114.9118	1057.9595	705.6421	6
5	2247.0641	2275.0590	2257.0485	1138.0332	N	1951.8484	976.4278	651.2877	5
6	2334.0962	2362.0911	2344.0805	1181.5492	S	**596.3510**	298.6791	199.4552	4
7	2435.1438	2463.1388	2445.1282	1232.0730	T	**509.3190**	255.1631	170.4445	3
8	2598.2072	2626.2021	2608.1915	1313.6047	Y	**408.2713**	204.6393	136.7619	2
9					R	**245.2080**	123.1076	82.4075	1

HCD MS2 spectrum of precursor m/z = 718.3570, z = 4^{+} at 1.2403 min. This spectrum was assigned to the EEQYNSTYR with N-glycan composition of H3N3F1.

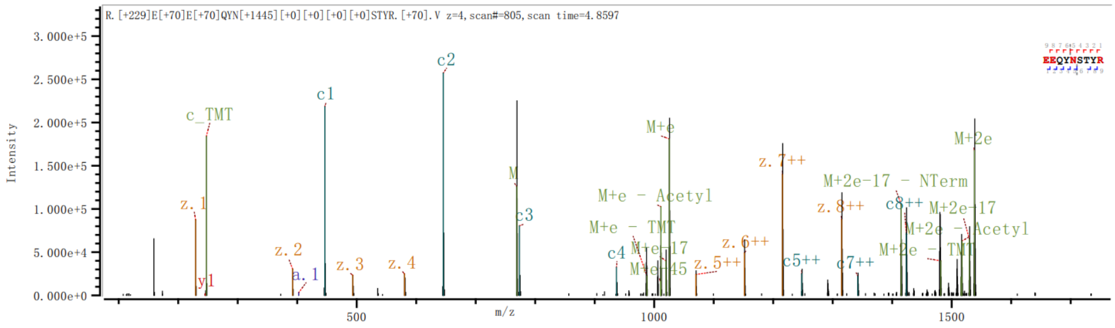

102: EEQYNSTYR

#	a. calc.	c calc.	c++ calc.	Seq.	y calc.	z. calc.	z.++ calc.	#
1	402.3210	**446.3347**	223.6710	E				9
2	601.4526	**645.4663**	323.2368	E	2645.1813	2629.1626	**1315.0849**	8
3	729.5112	**773.5248**	387.2661	Q	2446.0497	2430.0310	**1215.5191**	7
4	892.5745	**936.5882**	468.7977	Y	2317.9911	2301.9724	**1151.4898**	6
5	2451.1513	2495.1650	1248.0861	N	2154.9278	2138.9091	1069.9582	5
6	2538.1834	2582.1970	1291.6021	S	596.3510	**580.3323**		4
7	2639.2310	2683.2447	1342.1260	T	509.3190	**493.3002**		3
8	2802.2944	2846.3080	**1423.6576**	Y	408.2713	**392.2526**		2
9				R	245.2080	**229.1892**		1

ETD MS2 spectrum of precursor m/z = 769.1271, z = 4^{+} at 1.8597 min. This spectrum was assigned to the EEQYNSTYR with N-glycan composition of H3N4F1.

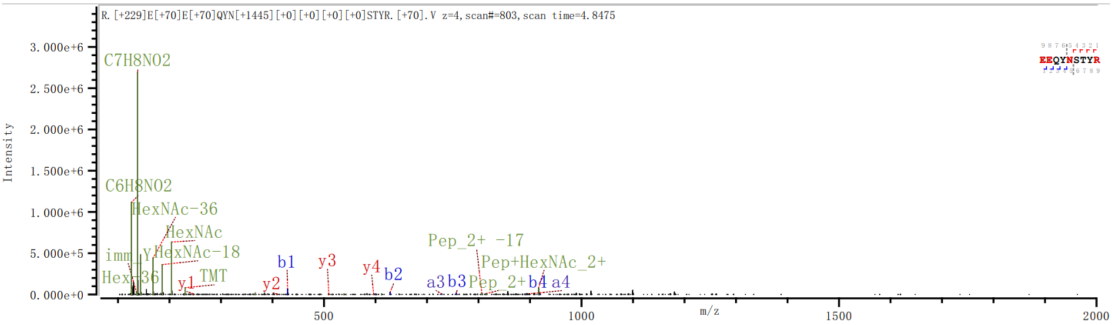

2351: EEQYNSTYR

#	a calc.	b calc.	b-18 calc.	b++ calc.	Seq.	y calc.	y++ calc.	y_3+ calc.	#
1	401.3132	**429.3081**	411.2976	215.1577	E				9
2	**600.4448**	**628.4397**	610.4291	314.7235	E	2645.1813	1323.0943	882.3986	8
3	**728.5034**	**756.4983**	738.4877	378.7528	Q	2446.0497	1223.5285	816.0214	7
4	891.5667	919.5616	901.5511	460.2844	Y	2317.9911	1159.4992	773.3352	6
5	2450.1435	2478.1384	2460.1279	1239.5728	N	2154.9278	1077.9675	718.9808	5
6	2537.1755	2565.1704	2547.1599	1283.0889	S	**596.3510**	298.6791	199.4552	4
7	2638.2232	2666.2181	2648.2076	1333.6127	T	**509.3190**	255.1631	170.4445	3
8	2801.2865	2829.2815	2811.2709	1415.1444	Y	**408.2713**	204.6393	136.7619	2
9					R	**245.2080**	123.1076	82.4075	1

HCD MS2 spectrum of precursor m/z = 769.1271, z = 4^{+} at 4.8475 min. This spectrum was assigned to the EEQYNSTYR with N-glycan composition of H3N4F1.

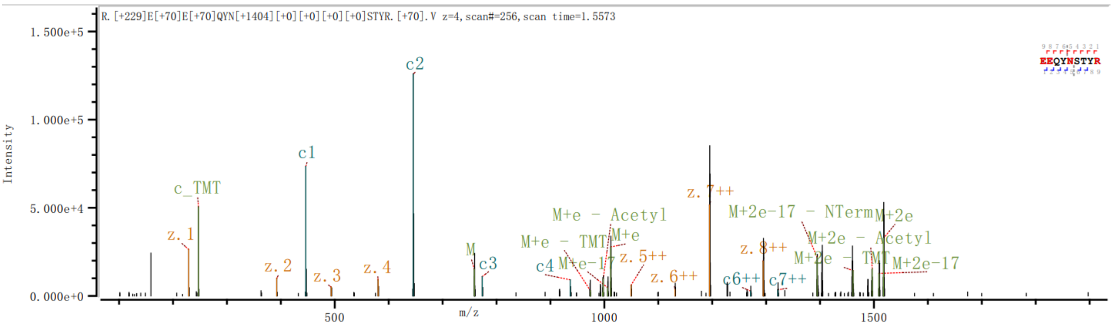

23: EEQYNSTYR

#	a. calc.	c calc.	c++ calc.	Seq.	y calc.	z. calc.	z.++ calc.	#
1	402.3210	**446.3347**	223.6710	E				9
2	601.4526	**645.4663**	323.2368	E	2604.1547	2588.1360	**1294.5716**	8
3	729.5112	**773.5248**	387.2661	Q	2405.0232	2389.0044	**1195.0059**	7
4	892.5745	**936.5882**	468.7977	Y	2276.9646	2260.9458	1130.9766	6
5	2410.1248	2454.1384	1227.5728	N	2113.9012	2097.8825	1049.4449	5
6	2497.1568	2541.1704	1271.0889	S	596.3510	**580.3323**		4
7	2598.2045	2642.2181	1321.6127	T	509.3190	**493.3002**		3
8	2761.2678	2805.2815	1403.1444	Y	408.2713	**392.2526**		2
9				R	245.2080	**229.1892**		1

ETD MS2 spectrum of precursor m/z = 758.8667, z = 4^+ at 1.5573 min. This spectrum was assigned to the EEQYNSTYR with N-glycan composition of H4N3F1.

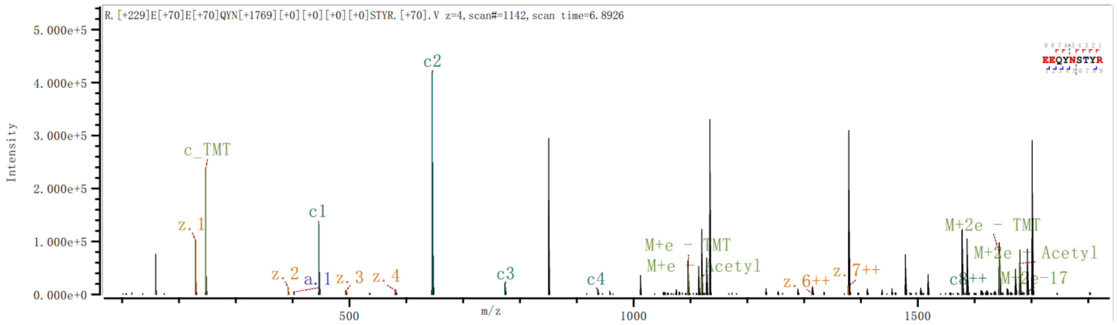

689: EEQYNSTYR

#	a. calc.	c calc.	c++ calc.	Seq.	y calc.	z. calc.	z.++ calc.	#
1	402.3210	**446.3347**	223.6710	E				9
2	601.4526	**645.4663**	323.2368	E	2969.2869	2953.2682	1477.1377	8
3	729.5112	**773.5248**	387.2661	Q	2770.1554	2754.1366	1377.5720	7
4	892.5745	**936.5882**	468.7977	Y	2642.0968	2626.0780	1313.5427	6
5	2775.2570	2819.2706	1410.1389	N	2479.0334	2463.0147	1232.0110	5
6	2862.2890	2906.3026	1453.6550	S	596.3510	**580.3323**		4
7	2963.3367	3007.3503	1504.1788	T	509.3190	**493.3002**		3
8	3126.4000	3170.4137	1585.7105	Y	408.2713	**392.2526**		2
9				R	245.2080	**229.1892**		1

HCD MS2 spectrum of precursor m/z = 850.1538, z = 4^+ at 6.8926 min. This spectrum was assigned to the EEQYNSTYR with N-glycan composition of H5N4F1.

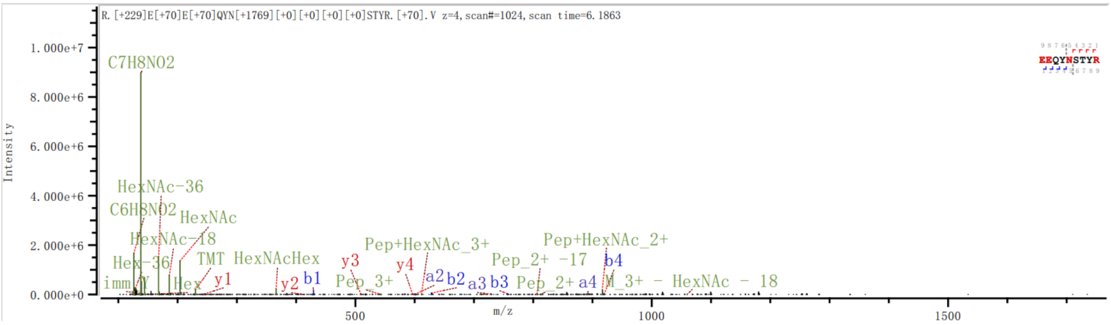

2925: EEQYNSTYR

#	a calc.	b calc.	b-18 calc.	b++ calc.	Seq.	y calc.	y++ calc.	y_3+ calc.	#
1	401.3132	**429.3081**	411.2976	215.1577	E				9
2	**600.4448**	**628.4397**	610.4291	314.7235	E	2969.2869	1485.1471	990.4338	8
3	**728.5034**	**756.4983**	738.4877	378.7528	Q	2770.1554	1385.5813	924.0566	7
4	891.5667	919.5616	901.5511	460.2844	Y	2642.0968	1321.5520	881.3704	6
5	2774.2492	2802.2441	2784.2335	1401.6257	N	2479.0334	1240.0204	827.0160	5
6	2861.2812	2889.2761	2871.2655	1445.1417	S	**596.3510**	298.6791	199.4552	4
7	2962.3289	2990.3238	2972.3132	1495.6655	T	**509.3190**	255.1631	170.4445	3
8	3125.3922	3153.3871	3135.3765	1577.1972	Y	**408.2713**	204.6393	136.7619	2
9					R	**245.2080**	123.1076	82.4075	1

HCD MS2 spectrum of precursor m/z = 850.1538, z = 4^+ at 6.1863 min. This spectrum was assigned to the EEQYNSTYR with N-glycan composition of H5N4F1.

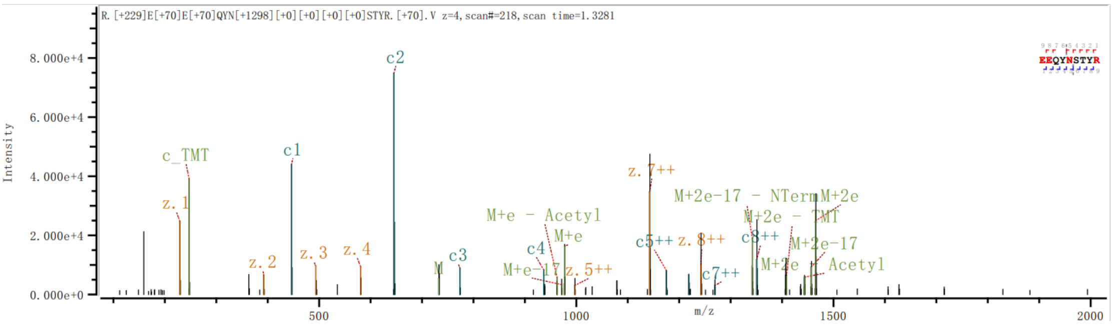

15: EEQYNSTYR

#	a. calc.	c calc.	c++ calc.	Seq.	y calc.	z. calc.	z.++ calc.	#
1	402.3210	**446.3347**	223.6710	E				9
2	601.4526	**645.4663**	323.2368	E	2499.1234	2483.1047	**1242.0560**	8
3	729.5112	**773.5248**	387.2661	Q	2299.9918	2283.9731	**1142.4902**	7
4	892.5745	**936.5882**	468.7977	Y	2171.9332	2155.9145	**1078.4609**	6
5	2305.0934	2349.1071	**1175.0572**	N	2008.8699	1992.8512	996.9292	5
6	2392.1254	2436.1391	**1218.5732**	S	596.3510	**580.3323**		4
7	2493.1731	2537.1868	1269.0970	T	509.3190	**493.3002**		3
8	2656.2365	2700.2501	**1350.6287**	Y	408.2713	**392.2526**		2
9				R	245.2080	**229.1892**		1

ETD MS2 spectrum of precursor m/z = 732.6162, z = 4^+ at 1.3281 min. This spectrum was assigned to the EEQYNSTYR with N-glycan composition of H3N4.

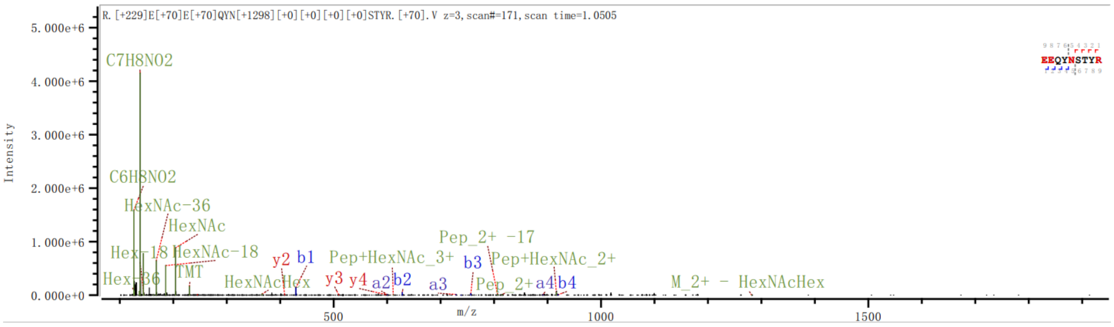

2257: EEQYNSTYR

#	a calc.	b calc.	b-18 calc.	b++ calc.	Seq.	y calc.	y++ calc.	#
1	401.3132	**429.3081**	411.2976	215.1577	E			9
2	**600.4448**	**628.4397**	610.4291	314.7235	E	2499.1234	1250.0653	8
3	**728.5034**	**756.4983**	738.4877	378.7528	Q	2299.9918	1150.4995	7
4	891.5667	919.5616	901.5511	460.2844	Y	2171.9332	1086.4702	6
5	2304.0856	2332.0805	2314.0699	1166.5439	N	2008.8699	1004.9386	5
6	2391.1176	2419.1125	2401.1020	1210.0599	S	**596.3510**	298.6791	4
7	2492.1653	2520.1602	2502.1497	1260.5837	T	**509.3190**	255.1631	3
8	2655.2286	2683.2235	2665.2130	1342.1154	Y	**408.2713**	204.6393	2
9					R	**245.2080**	123.1076	1

HCD MS2 spectrum of precursor m/z = 976.4789, z = 4^+ at 1.0505 min. This spectrum was assigned to the EEQYNSTYR with N-glycan composition of H3N4.

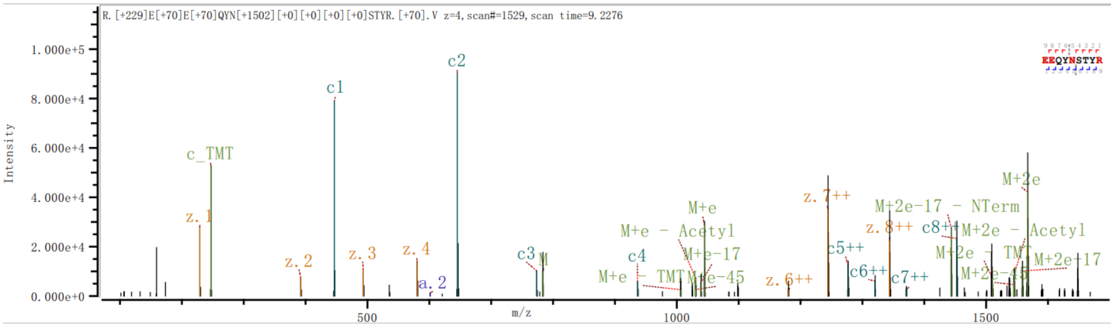

286: EEQYNSTYR

#	a. calc.	c calc.	c++ calc.	Seq.	y calc.	z. calc.	z.++ calc.	#
1	402.3210	**446.3347**	223.6710	E				9
2	**601.4526**	**645.4663**	323.2368	E	2702.2028	2686.1840	**1343.5957**	8
3	729.5112	**773.5248**	387.2661	Q	2503.0712	2487.0524	**1244.0299**	7
4	892.5745	**936.5882**	468.7977	Y	2375.0126	2358.9939	**1180.0006**	6
5	2508.1728	2552.1864	1276.5969	N	2211.9493	2195.9305	1098.4689	5
6	2595.2048	2639.2185	1320.1129	S	596.3510	**580.3323**		4
7	2696.2525	2740.2661	1370.6367	T	509.3190	**493.3002**		3
8	2859.3158	2903.3295	**1452.1684**	Y	408.2713	**392.2526**		2
9				R	245.2080	**229.1892**		1

ETD MS2 spectrum of precursor m/z = 783.3797, z = 4^+ at 9.2276 min. This spectrum was assigned to the EEQYNSTYR with N-glycan composition of H3N5.

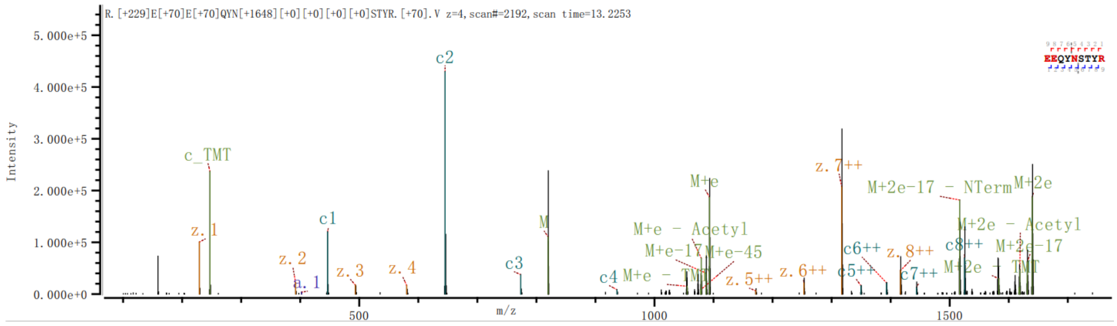

357: EEQYNSTYR

#	a. calc.	c calc.	c++ calc.	Seq.	y calc.	z. calc.	z.++ calc.	#
1	402.3210	**446.3347**	223.6710	E				9
2	601.4526	**645.4663**	323.2368	E	2848.2607	2832.2419	**1416.6246**	8
3	729.5112	**773.5248**	387.2661	Q	2649.1291	2633.1103	**1317.0588**	7
4	892.5745	**936.5882**	468.7977	Y	2521.0705	2505.0518	**1253.0295**	6
5	2654.2307	2698.2443	1349.6258	N	2358.0072	2341.9884	**1171.4979**	5
6	2741.2627	2785.2764	1393.1418	S	596.3510	**580.3323**		4
7	2842.3104	2886.3240	1443.6657	T	509.3190	**493.3002**		3
8	3005.3737	3049.3874	**1525.1973**	Y	408.2713	**392.2526**		2
9				R	245.2080	**229.1892**		1

ETD MS2 spectrum of precursor m/z = 819.8960, z = 4^+ at 13.2253 min. This spectrum was assigned to the EEQYNSTYR with N-glycan composition of H3N5F1.

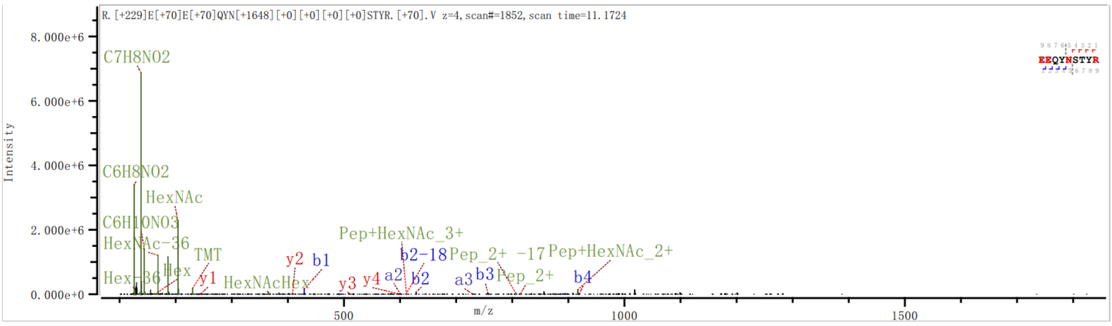

2574: EEQYNSTYR

#	a calc.	b calc.	b-18 calc.	b++ calc.	Seq.	y calc.	y++ calc.	y_3+ calc.	#
1	401.3132	**429.3081**	411.2976	215.1577	E				9
2	**600.4448**	**628.4397**	610.4291	314.7235	E	2848.2607	1424.6340	950.0917	8
3	**728.5034**	**756.4983**	738.4877	378.7528	Q	2649.1291	1325.0682	883.7145	7
4	891.5667	919.5616	901.5511	460.2844	Y	2521.0705	1261.0389	841.0283	6
5	2653.2229	2681.2178	2663.2072	1341.1125	N	2358.0072	1179.5072	786.6739	5
6	2740.2549	2768.2498	2750.2393	1384.6285	S	**596.3510**	298.6791	199.4552	4
7	2841.3026	2869.2975	2851.2869	1435.1524	T	**509.3190**	255.1631	170.4445	3
8	3004.3659	3032.3608	3014.3503	1516.6841	Y	**408.2713**	204.6393	136.7619	2
9					R	**245.2080**	123.1076	82.4075	1

HCD MS2 spectrum of precursor m/z = 819.8960, z = 4^+ at 11.1724 min. This spectrum was assigned to the EEQYNSTYR with N-glycan composition of H3N5F1.

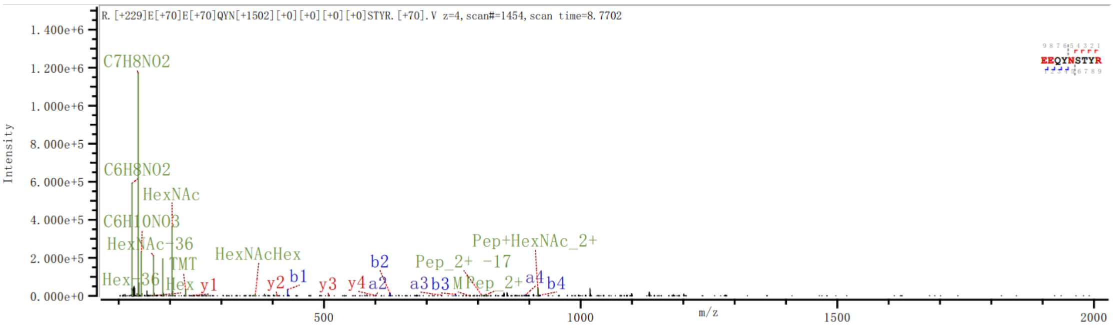

2523: EEQYNSTYR

#	a calc.	b calc.	b-18 calc.	b++ calc.	Seq.	y calc.	y++ calc.	y_3+ calc.	#
1	401.3132	**429.3081**	411.2976	215.1577	E				9
2	600.4448	**628.4397**	610.4291	314.7235	E	2702.2028	1351.6050	901.4058	8
3	**728.5034**	**756.4983**	738.4877	378.7528	Q	2503.0712	1252.0392	835.0286	7
4	891.5667	919.5616	901.5511	460.2844	Y	2375.0126	1188.0099	792.3424	6
5	2507.1650	2535.1599	2517.1493	1268.0836	N	2211.9493	1106.4783	737.9879	5
6	2594.1970	2622.1919	2604.1813	1311.5996	S	**596.3510**	298.6791	199.4552	4
7	2695.2447	2723.2396	2705.2290	1362.1234	T	**509.3190**	255.1631	170.4445	3
8	2858.3080	2886.3029	2868.2924	1443.6551	Y	**408.2713**	204.6393	136.7619	2
9					R	**245.2080**	123.1076	82.4075	1

HCD MS2 spectrum of precursor m/z = 783.3805, z = 4^+ at 8.7702 min. This spectrum was assigned to the EEQYNSTYR with N-glycan composition of H3N5.

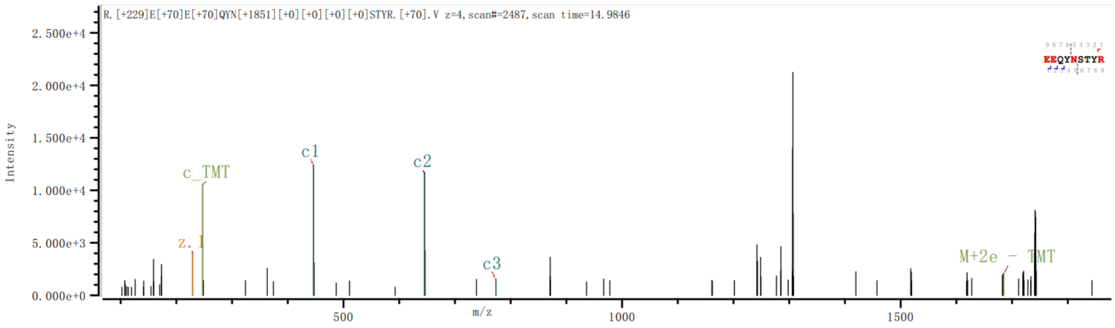

902: EEQYNSTYR

#	a. calc.	c calc.	c++ calc.	Seq.	y calc.	z. calc.	z.++ calc.	#
1	402.3210	**446.3347**	223.6710	E				9
2	601.4526	**645.4663**	323.2368	E	3051.3400	3035.3213	1518.1643	8
3	729.5112	**773.5248**	387.2661	Q	2852.2084	2836.1897	1418.5985	7
4	892.5745	936.5882	468.7977	Y	2724.1499	2708.1311	1354.5692	6
5	2857.3101	2901.3237	1451.1655	N	2561.0865	2545.0678	1273.0375	5
6	2944.3421	2988.3557	1494.6815	S	596.3510	580.3323		4
7	3045.3898	3089.4034	1545.2053	T	509.3190	493.3002		3
8	3208.4531	3252.4667	1626.7370	Y	408.2713	392.2526		2
9				R	245.2080	**229.1892**		1

ETD MS2 spectrum of precursor m/z = 870.6630, z = 4^{+} at 14.9846 min. This spectrum was assigned to the EEQYNSTYR with N-glycan composition of H3N6F1.

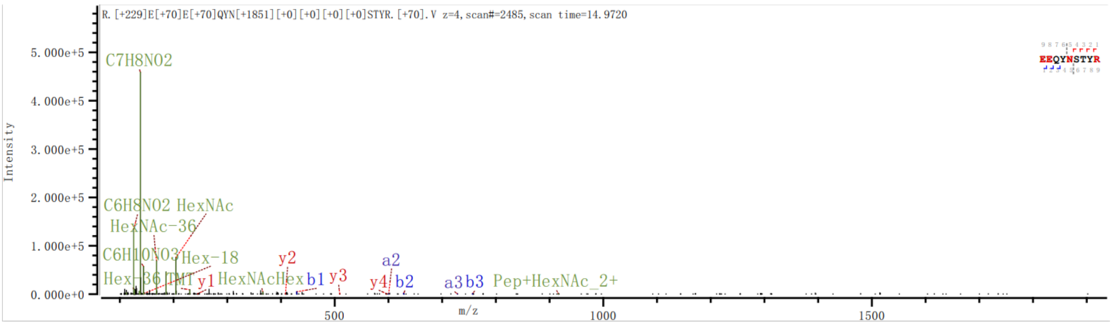

3151: EEQYNSTYR

#	a calc.	b calc.	b-18 calc.	b++ calc.	Seq.	y calc.	y++ calc.	y_3+ calc.	#
1	401.3132	**429.3081**	411.2976	215.1577	E				9
2	600.4448	**628.4397**	610.4291	314.7235	E	3051.3400	1526.1737	1017.7849	8
3	**728.5034**	**756.4983**	738.4877	378.7528	Q	2852.2084	1426.6079	951.4077	7
4	891.5667	919.5616	901.5511	460.2844	Y	2724.1499	1362.5786	908.7215	6
5	2856.3022	2884.2972	2866.2866	1442.6522	N	2561.0865	1281.0469	854.3670	5
6	2943.3343	2971.3292	2953.3186	1486.1682	S	**596.3510**	298.6791	199.4552	4
7	3044.3820	3072.3769	3054.3663	1536.6921	T	509.3190	255.1631	170.4445	3
8	3207.4453	3235.4402	3217.4296	1618.2237	Y	408.2713	204.6393	136.7619	2
9					R	245.2080	123.1076	82.4075	1

HCD MS2 spectrum of precursor m/z = 870.6630, z = 4^{+} at 14.9720 min. This spectrum was assigned to the EEQYNSTYR with N-glycan composition of H3N6F1.

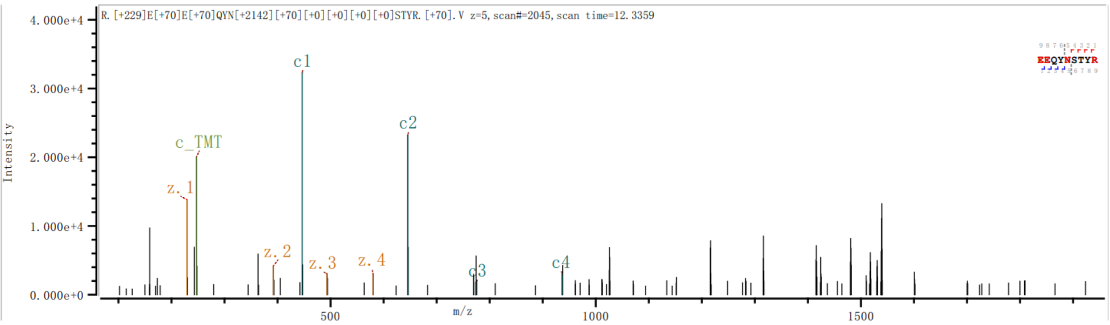

341: EEQYNSTYR

#	a. calc.	c calc.	c++ calc.	Seq.	y calc.	z. calc.	z.++ calc.	#
1	402.3210	**446.3347**	223.6710	E				9
2	601.4526	**645.4663**	323.2368	E	2645.1813	2629.1626	1315.0849	8
3	729.5112	**773.5248**	387.2661	Q	2446.0497	2430.0310	1215.5191	7
4	892.5745	**936.5882**	468.7977	Y	2317.9911	2301.9724	**1151.4898**	6
5	2451.1513	2495.1650	1248.0861	N	2154.9278	2138.9091	1069.9582	5
6	2538.1834	2582.1970	1291.6021	S	596.3510	**580.3323**		4
7	2639.2310	2683.2447	1342.1260	T	509.3190	**493.3002**		3
8	2802.2944	2846.3080	1423.6576	Y	408.2713	**392.2526**		2
9				R	245.2080	**229.1892**		1

HCD MS2 spectrum of precursor m/z = 769.1237, z = 4^{+} at 12.3359 min. This spectrum was assigned to the EEQYNSTYR with N-glycan composition of H3N6F1S1.

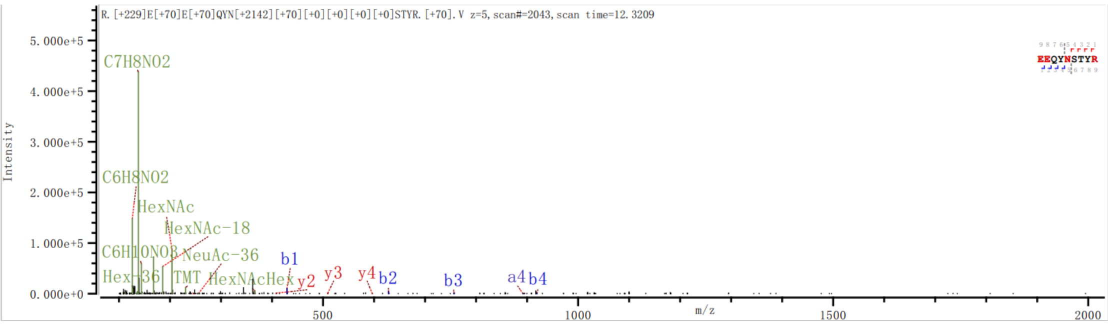

2590: EEQYNSTYR

#	a calc.	b calc.	b-18 calc.	b++ calc.	Seq.	y calc.	y++ calc.	y_3+ calc.	#
1	401.3132	**429.3081**	411.2976	215.1577	E				9
2	600.4448	**628.4397**	610.4291	314.7235	E	2645.1813	1323.0943	882.3986	8
3	728.5034	**756.4983**	738.4877	378.7528	Q	2446.0497	1223.5285	816.0214	7
4	891.5667	919.5616	901.5511	460.2844	Y	2317.9911	1159.4992	773.3352	6
5	2450.1435	2478.1384	2460.1279	1239.5728	N	2154.9278	1077.9675	718.9808	5
6	2537.1755	2565.1704	2547.1599	1283.0889	S	596.3510	298.6791	199.4552	4
7	2638.2232	2666.2181	2648.2076	1333.6127	T	**509.3190**	255.1631	170.4445	3
8	2801.2865	2829.2815	2811.2709	1415.1444	Y	**408.2713**	204.6393	136.7619	2
9					R	245.2080	123.1076	82.4075	1

HCD MS2 spectrum of precursor m/z = 769.1237, z = 4^{+} at 12.3209 min. This spectrum was assigned to the EEQYNSTYR with N-glycan composition of H3N6F1S1.

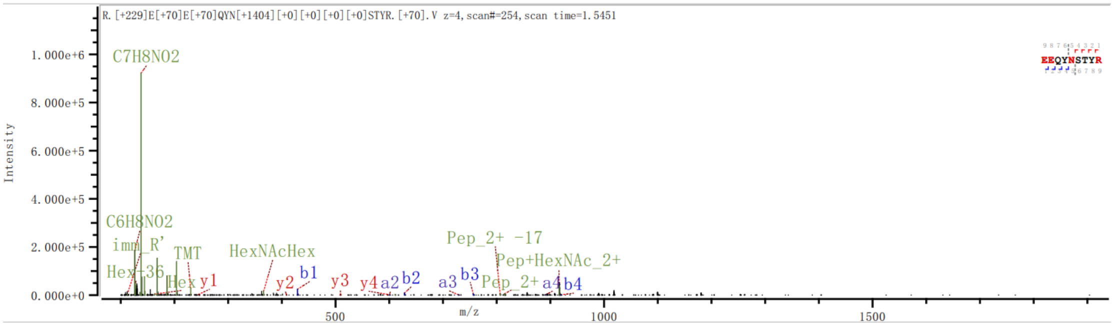

2272: EEQYNSTYR

#	a calc.	b calc.	b-18 calc.	b++ calc.	Seq.	y calc.	y++ calc.	y_3+ calc.	#
1	401.3132	**429.3081**	411.2976	215.1577	E				9
2	600.4448	**628.4397**	610.4291	314.7235	E	2604.1547	1302.5810	868.7231	8
3	**728.5034**	**756.4983**	738.4877	378.7528	Q	2405.0232	1203.0152	802.3459	7
4	891.5667	919.5616	901.5511	460.2844	Y	2276.9646	1138.9859	759.6597	6
5	2409.1170	2437.1119	2419.1013	1219.0596	N	2113.9012	1057.4543	705.3053	5
6	2496.1490	2524.1439	2506.1333	1262.5756	S	**596.3510**	298.6791	199.4552	4
7	2597.1967	2625.1916	2607.1810	1313.0994	T	**509.3190**	255.1631	170.4445	3
8	2760.2600	2788.2549	2770.2443	1394.6311	Y	**408.2713**	204.6393	136.7619	2
9					R	**245.2080**	123.1076	82.4075	1

HCD MS2 spectrum of precursor m/z = 769.1237, z = 4^+ at 1.5451 min. This spectrum was assigned to the EEQYNSTYR with N-glycan composition of H4N3F1.

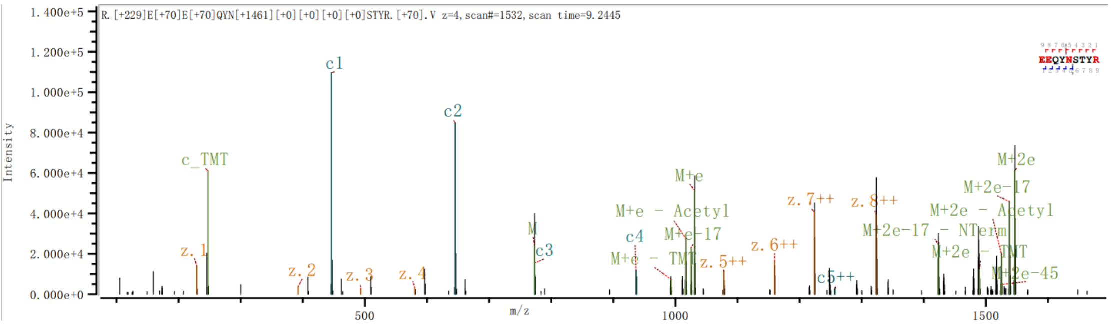

288: EEQYNSTYR

#	a. calc.	c calc.	c++ calc.	Seq.	y calc.	z. calc.	z.++ calc.	#
1	402.3210	**446.3347**	223.6710	E				9
2	601.4526	**645.4663**	323.2368	E	2661.1762	2645.1575	**1323.0824**	8
3	729.5112	**773.5248**	387.2661	Q	2462.0446	2446.0259	**1223.5166**	7
4	892.5745	**936.5882**	468.7977	Y	2333.9860	2317.9673	**1159.4873**	6
5	2467.1462	2511.1599	1256.0836	N	2170.9227	2154.9040	1077.9556	5
6	2554.1783	2598.1919	1299.5996	S	596.3510	**580.3323**		4
7	2655.2259	2699.2396	1350.1234	T	509.3190	493.3002		3
8	2818.2893	2862.3029	1431.6551	Y	408.2713	**392.2526**		2
9				R	245.2080	**229.1892**		1

ETD MS2 spectrum of precursor m/z = 773.1226, z = 4^{+} at 9.2445 min. This spectrum was assigned to the EEQYNSTYR with N-glycan composition of H4N4.

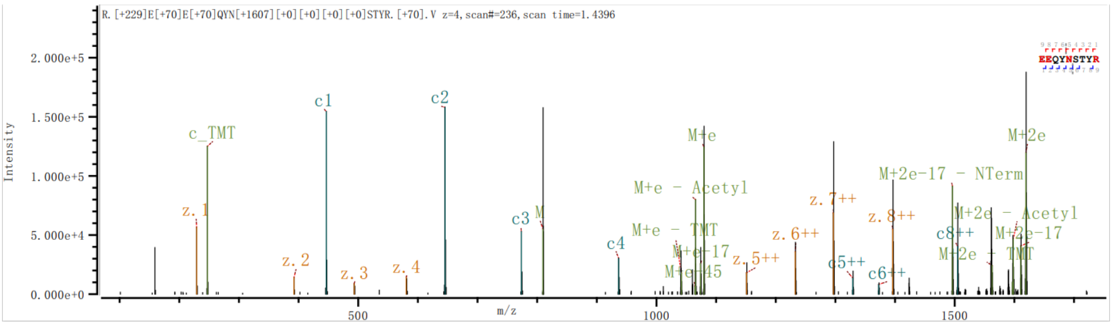

17: EEQYNSTYR

#	a. calc.	c calc.	c++ calc.	Seq.	y calc.	z. calc.	z.++ calc.	#
1	402.3210	**446.3347**	223.6710	E				9
2	601.4526	**645.4663**	323.2368	E	2807.2341	2791.2154	**1396.1113**	8
3	729.5112	**773.5248**	387.2661	Q	2608.1025	2592.0838	**1296.5455**	7
4	892.5745	**936.5882**	468.7977	Y	2480.0439	2464.0252	**1232.5162**	6
5	2613.2041	2657.2178	1329.1125	N	2316.9806	2300.9619	**1150.9846**	5
6	2700.2362	2744.2498	1372.6285	S	596.3510	**580.3323**		4
7	2801.2839	2845.2975	1423.1524	T	509.3190	**493.3002**		3
8	2964.3472	3008.3608	1504.6841	Y	408.2713	**392.2526**		2
9				R	245.2080	**229.1892**		1

ETD MS2 spectrum of precursor m/z = 809.6364, z = 4^+ at 1.4396 min. This spectrum was assigned to the EEQYNSTYR with N-glycan composition of H4N4F1.

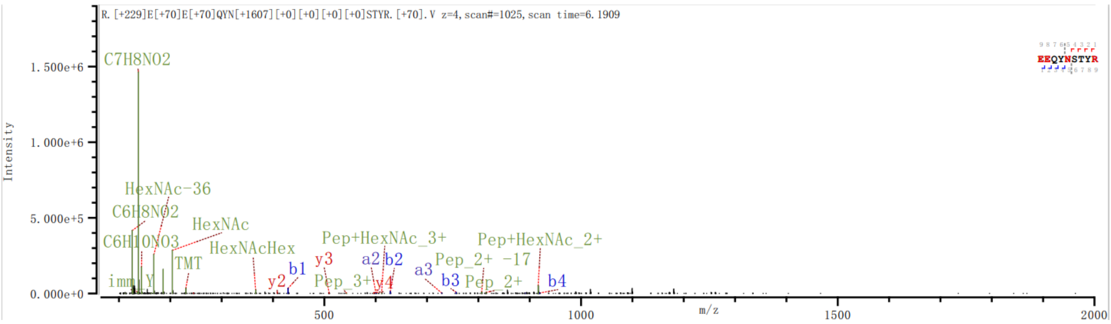

2395: EEQYNSTYR

#	a calc.	b calc.	b-18 calc.	b++ calc.	Seq.	y calc.	y++ calc.	y_3+ calc.	#
1	401.3132	**429.3081**	411.2976	215.1577	E				9
2	**600.4448**	**628.4397**	610.4291	314.7235	E	2807.2341	1404.1207	936.4162	8
3	**728.5034**	**756.4983**	738.4877	378.7528	Q	2608.1025	1304.5549	870.0390	7
4	891.5667	919.5616	901.5511	460.2844	Y	2480.0439	1240.5256	827.3528	6
5	2612.1963	2640.1912	2622.1807	1320.5993	N	2316.9806	1158.9939	772.9984	5
6	2699.2284	2727.2233	2709.2127	1364.1153	S	**596.3510**	298.6791	199.4552	4
7	2800.2760	2828.2709	2810.2604	1414.6391	T	**509.3190**	255.1631	170.4445	3
8	2963.3394	2991.3343	2973.3237	1496.1708	Y	**408.2713**	204.6393	136.7619	2
9					R	**245.2080**	123.1076	82.4075	1

ETD MS2 spectrum of precursor m/z = 809.6364, z = 4^{+} at 6.1909 min. This spectrum was assigned to the EEQYNSTYR with N-glycan composition of H4N4F1.

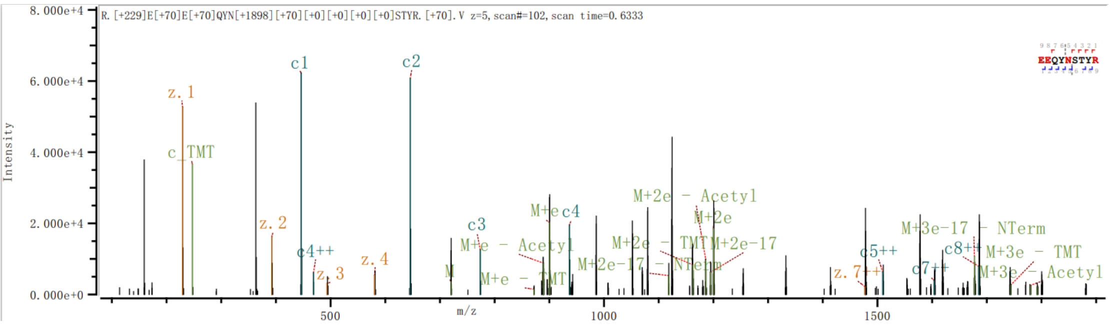

574: EEQYNSTYR

#	a. calc.	c calc.	c++ calc.	Seq.	y calc.	z. calc.	z.++ calc.	#
1	402.3210	**446.3347**	223.6710	E				9
2	601.4526	**645.4663**	323.2368	E	3168.4186	3152.3999	1576.7036	8
3	729.5112	**773.5248**	387.2661	Q	2969.2870	2953.2683	1477.1378	7
4	892.5745	936.5882	468.7977	Y	2841.2285	2825.2097	1413.1085	6
5	2974.3887	3018.4023	1509.7048	N	2678.1651	2662.1464	**1331.5768**	5
6	3061.4207	3105.4343	1553.2208	S	596.3510	**580.3323**		4
7	3162.4684	3206.4820	1603.7446	T	509.3190	493.3002		3
8	3325.5317	3369.5453	1685.2763	Y	408.2713	**392.2526**		2
9				R	245.2080	**229.1892**		1

ETD MS2 spectrum of precursor m/z = 720.1499, z = 5^+ at 0.6333 min. This spectrum was assigned to the EEQYNSTYR with N-glycan composition of H4N4F1S1.

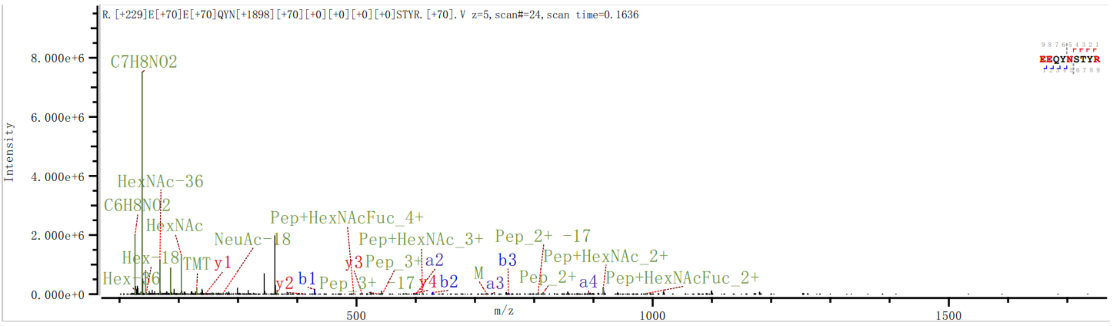

2809: EEQYNSTYR

#	a calc.	b calc.	b-18 calc.	b++ calc.	b_3+ calc.	Seq.	y calc.	y++ calc.	y_3+ calc.	#
1	401.3132	**429.3081**	411.2976	215.1577	143.7742	E				9
2	600.4448	**628.4397**	610.4291	314.7235	210.1514	E	3168.4186	1584.7130	1056.8111	8
3	728.5034	**756.4983**	738.4877	378.7528	252.8376	Q	2969.2870	1485.1472	990.4339	7
4	891.5667	919.5616	901.5511	460.2844	307.1921	Y	2841.2285	1421.1179	947.7477	6
5	2973.3808	3001.3758	2983.3652	1501.1915	1001.1301	N	2678.1651	1339.5862	893.3932	5
6	3060.4129	3088.4078	3070.3972	1544.7075	1030.1408	S	**596.3510**	298.6791	199.4552	4
7	3161.4606	3189.4555	3171.4449	1595.2314	1063.8233	T	509.3190	255.1631	170.4445	3
8	3324.5239	3352.5188	3334.5082	1676.7630	1118.1778	Y	**408.2713**	204.6393	136.7619	2
9						R	245.2080	123.1076	82.4075	1

ETD MS2 spectrum of precursor m/z = 720.1499, z = 5^+ at 0.1636 min. This spectrum was assigned to the EEQYNSTYR with N-glycan composition of H4N4F1S1.

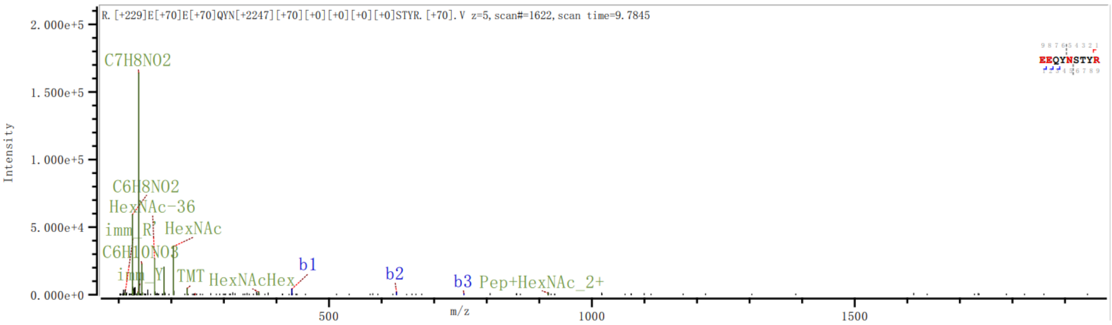

3957: EEQYNSTYR

#	a calc.	b calc.	b-18 calc.	b++ calc.	b_3+ calc.	Seq.	y calc.	y++ calc.	y_3+ calc.	#
1	401.3132	**429.3081**	411.2976	215.1577	143.7742	E				9
2	600.4448	**628.4397**	610.4291	314.7235	210.1514	E	3517.5559	1759.2816	1173.1902	8
3	728.5034	**756.4983**	738.4877	378.7528	252.8376	Q	3318.4243	1659.7158	1106.8130	7
4	891.5667	919.5616	901.5511	460.2844	307.1921	Y	3190.3657	1595.6865	1064.1268	6
5	3322.5181	3350.5130	3332.5025	1675.7602	1117.5092	N	3027.3024	1514.1548	1009.7723	5
6	3409.5502	3437.5451	3419.5345	1719.2762	1146.5199	S	596.3510	298.6791	199.4552	4
7	3510.5978	3538.5927	3520.5822	1769.8000	1180.2024	T	509.3190	255.1631	170.4445	3
8	3673.6612	3701.6561	3683.6455	1851.3317	1234.5569	Y	408.2713	204.6393	136.7619	2
9						R	**245.2080**	123.1076	82.4075	1

HCD MS2 spectrum of precursor m/z = 789.9729, z = 5^+ at 9.7845 min. This spectrum was assigned to the EEQYNSTYR with N-glycan composition of H4N4F2S1.

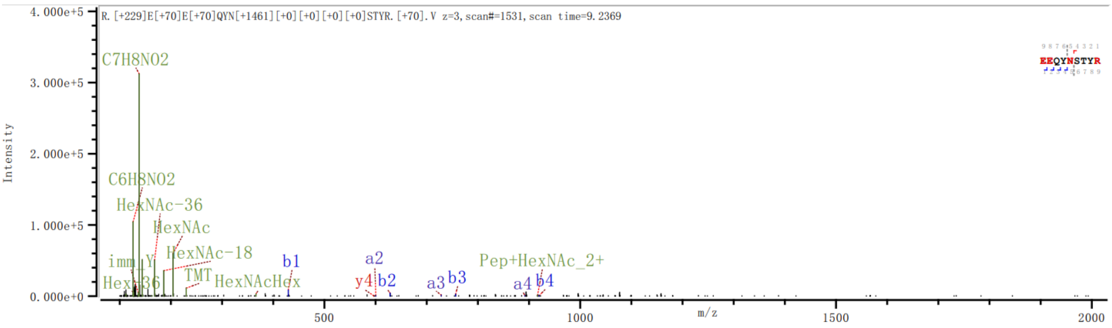

2539: EEQYNSTYR

#	a calc.	b calc.	b-18 calc.	b++ calc.	Seq.	y calc.	y++ calc.	#
1	401.3132	**429.3081**	411.2976	215.1577	E			9
2	**600.4448**	**628.4397**	610.4291	314.7235	E	2661.1762	1331.0917	8
3	**728.5034**	**756.4983**	738.4877	378.7528	Q	2462.0446	1231.5259	7
4	891.5667	919.5616	901.5511	460.2844	Y	2333.9860	1167.4967	6
5	2466.1384	2494.1333	2476.1228	1247.5703	N	2170.9227	1085.9650	5
6	2553.1704	2581.1654	2563.1548	1291.0863	S	**596.3510**	298.6791	4
7	2654.2181	2682.2130	2664.2025	1341.6102	T	509.3190	255.1631	3
8	2817.2815	2845.2764	2827.2658	1423.1418	Y	408.2713	204.6393	2
9					R	245.2080	123.1076	1

HCD MS2 spectrum of precursor m/z = 1030.4966, z = 3^+ at 9.2369 min. This spectrum was assigned to the EEQYNSTYR with N-glycan composition of H4N4.

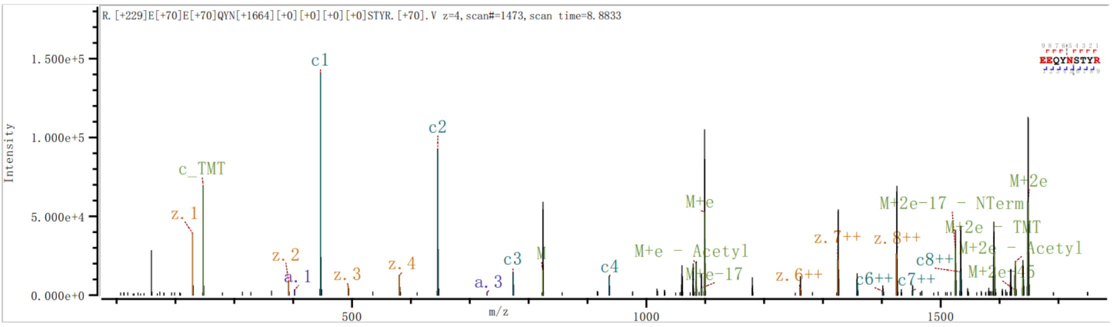

278: EEQYNSTYR

#	a. calc.	c calc.	c++ calc.	Seq.	y calc.	z. calc.	z.++ calc.	#
1	402.3210	**446.3347**	223.6710	E				9
2	601.4526	**645.4663**	323.2368	E	2864.2556	2848.2369	**1424.6221**	8
3	**729.5112**	**773.5248**	387.2661	Q	2665.1240	2649.1053	**1325.0563**	7
4	892.5745	**936.5882**	468.7977	Y	2537.0654	2521.0467	**1261.0270**	6
5	2670.2256	2714.2393	1357.6233	N	2374.0021	2357.9834	**1179.4953**	5
6	2757.2576	2801.2713	1401.1393	S	596.3510	**580.3323**		4
7	2858.3053	2902.3190	1451.6631	T	509.3190	**493.3002**		3
8	3021.3687	3065.3823	1533.1948	Y	408.2713	**392.2526**		2
9				R	245.2080	**229.1892**		1

ETD MS2 spectrum of precursor m/z = 823.8933, z = 4^+ at 8.8833 min. This spectrum was assigned to the EEQYNSTYR with N-glycan composition of H4N5.

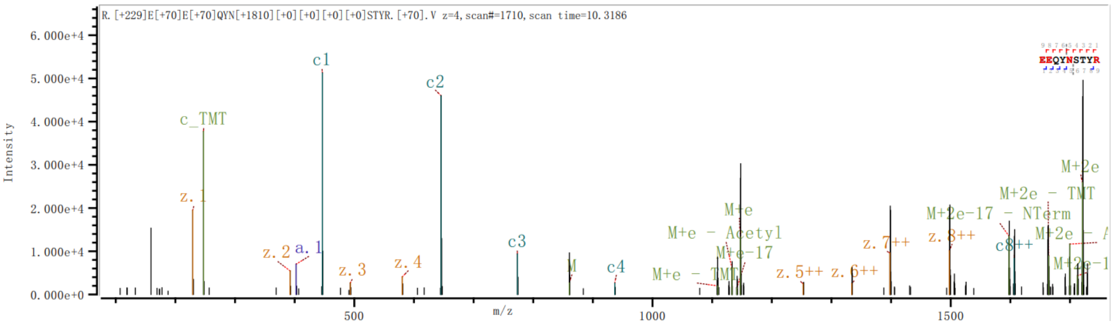

777: EEQYNSTYR

#	a. calc.	c calc.	c++ calc.	Seq.	y calc.	z. calc.	z.++ calc.	#
1	**402.3210**	**446.3347**	223.6710	E				9
2	601.4526	**645.4663**	323.2368	E	3010.3135	2994.2948	**1497.6510**	8
3	729.5112	**773.5248**	387.2661	Q	2811.1819	2795.1632	**1398.0852**	7
4	892.5745	**936.5882**	468.7977	Y	2683.1233	2667.1046	1334.0559	6
5	2816.2835	2860.2972	1430.6522	N	2520.0600	2504.0413	**1252.5243**	5
6	2903.3156	2947.3292	1474.1682	S	596.3510	**580.3323**		4
7	3004.3632	3048.3769	1524.6921	T	509.3190	**493.3002**		3
8	3167.4266	3211.4402	1606.2237	Y	408.2713	**392.2526**		2
9				R	245.2080	**229.1892**		1

ETD MS2 spectrum of precursor m/z = 860.4086, $z = 4^{+}$ at 10.3186 min. This spectrum was assigned to the EEQYNSTYR with N-glycan composition of H4N5F1.

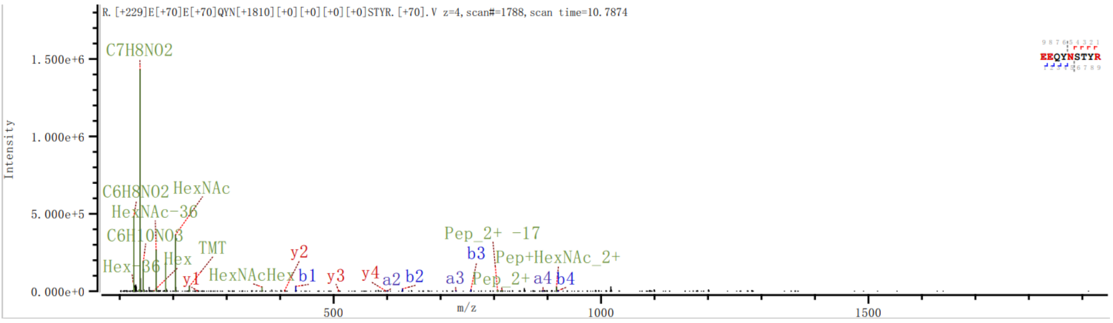

3041: EEQYNSTYR

#	a calc.	b calc.	b-18 calc.	b++ calc.	Seq.	y calc.	y++ calc.	y_3+ calc.	#
1	401.3132	**429.3081**	411.2976	215.1577	E				9
2	**600.4448**	**628.4397**	610.4291	314.7235	E	3010.3135	1505.6604	1004.1093	8
3	**728.5034**	**756.4983**	738.4877	378.7528	Q	2811.1819	1406.0946	937.7322	7
4	891.5667	919.5616	901.5511	460.2844	Y	2683.1233	1342.0653	895.0460	6
5	2815.2757	2843.2706	2825.2601	1422.1389	N	2520.0600	1260.5336	840.6915	5
6	2902.3077	2930.3026	2912.2921	1465.6550	S	**596.3510**	298.6791	199.4552	4
7	3003.3554	3031.3503	3013.3398	1516.1788	T	**509.3190**	255.1631	170.4445	3
8	3166.4187	3194.4137	3176.4031	1597.7105	Y	**408.2713**	204.6393	136.7619	2
9					R	**245.2080**	123.1076	82.4075	1

HCD MS2 spectrum of precursor m/z = 860.4084, z = 4^{+} at 10.7874 min. This spectrum was assigned to the EEQYNSTYR with N-glycan composition of H4N5F1.

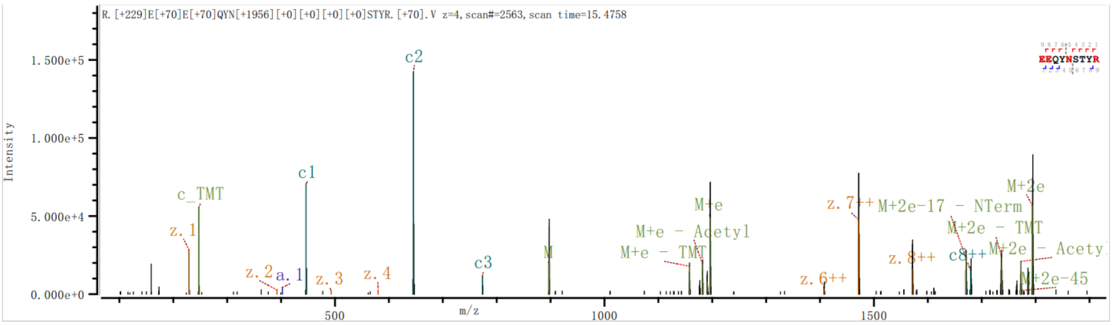

922: EEQYNSTYR

#	a. calc.	c calc.	c++ calc.	Seq.	y calc.	z. calc.	z.++ calc.	#
1	**402.3210**	**446.3347**	223.6710	E				9
2	601.4526	**645.4663**	323.2368	E	3156.3714	3140.3527	**1570.6800**	8
3	729.5112	**773.5248**	387.2661	Q	2957.2398	2941.2211	**1471.1142**	7
4	892.5745	936.5882	468.7977	Y	2829.1812	2813.1625	1407.0849	6
5	2962.3414	3006.3551	1503.6812	N	2666.1179	2650.0992	1325.5532	5
6	3049.3735	3093.3871	1547.1972	S	596.3510	**580.3323**		4
7	3150.4211	3194.4348	1597.7210	T	509.3190	**493.3002**		3
8	3313.4845	3357.4981	1679.2527	Y	408.2713	**392.2526**		2
9				R	245.2080	**229.1892**		1

ETD MS2 spectrum of precursor m/z = 896.9238, z = 4^+ at 15.4758 min. This spectrum was assigned to the EEQYNSTYR with N-glycan composition of H4N5F2.

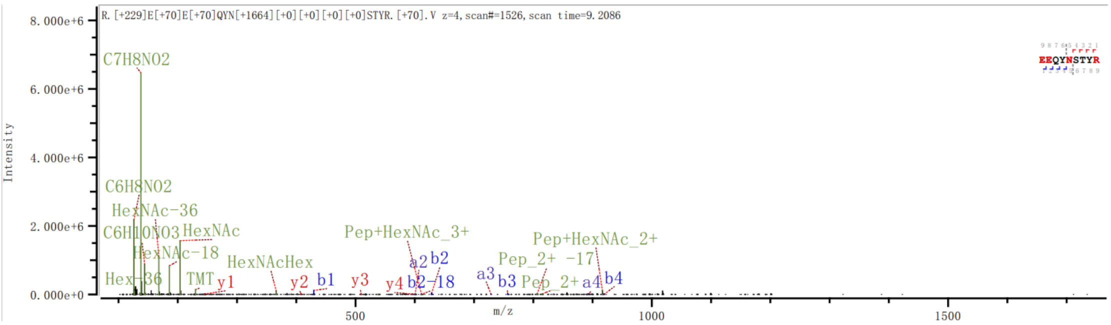

2533: EEQYNSTYR

#	a calc.	b calc.	b-18 calc.	b++ calc.	Seq.	y calc.	y++ calc.	y_3+ calc.	#
1	401.3132	**429.3081**	411.2976	215.1577	E				9
2	**600.4448**	**628.4397**	610.4291	314.7235	E	2864.2556	1432.6314	955.4234	8
3	**728.5034**	**756.4983**	738.4877	378.7528	Q	2665.1240	1333.0656	889.0462	7
4	891.5667	919.5616	901.5511	460.2844	Y	2537.0654	1269.0363	846.3600	6
5	2669.2178	2697.2127	2679.2021	1349.1100	N	2374.0021	1187.5047	792.0055	5
6	2756.2498	2784.2447	2766.2342	1392.6260	S	**596.3510**	298.6791	199.4552	4
7	2857.2975	2885.2924	2867.2819	1443.1498	T	**509.3190**	255.1631	170.4445	3
8	3020.3608	3048.3557	3030.3452	1524.6815	Y	**408.2713**	204.6393	136.7619	2
9					R	**245.2080**	123.1076	82.4075	1

ETD MS2 spectrum of precursor m/z = 823.8941, z = 4^+ at 9.2086 min. This spectrum was assigned to the EEQYNSTYR with N-glycan composition of H4N5.

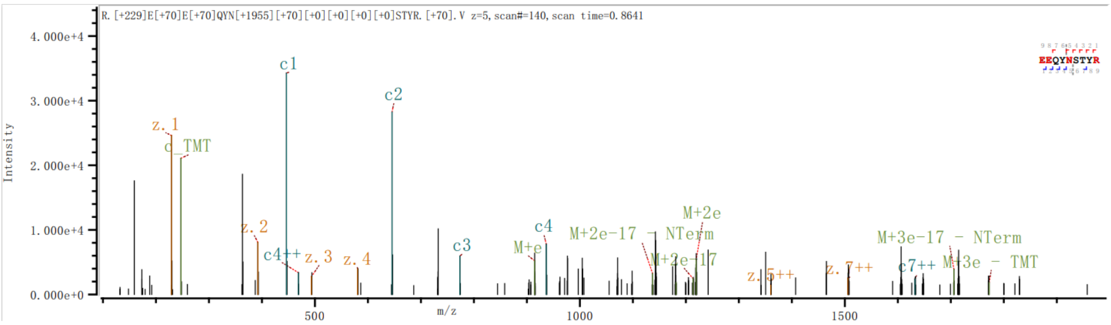

1272: EEQYNSTYR

#	a. calc.	c calc.	c++ calc.	Seq.	y calc.	z. calc.	z.++ calc.	#
1	402.3210	**446.3347**	223.6710	E				9
2	601.4526	**645.4663**	323.2368	E	3225.4401	3209.4214	1605.2143	8
3	729.5112	**773.5248**	387.2661	Q	3026.3085	3010.2898	**1505.6485**	7
4	892.5745	**936.5882**	468.7977	Y	2898.2499	2882.2312	1441.6192	6
5	3031.4101	3075.4238	1538.2155	N	2735.1866	2719.1679	**1360.0876**	5
6	3118.4422	3162.4558	1581.7315	S	596.3510	**580.3323**		4
7	3219.4898	3263.5035	1632.2554	T	509.3190	**493.3002**		3
8	3382.5532	3426.5668	1713.7870	Y	408.2713	**392.2526**		2
9				R	245.2080	**229.1892**		1

ETD MS2 spectrum of precursor m/z = 731.5514, z = 5^{+} at 0.8641 min. This spectrum was assigned to the EEQYNSTYR with N-glycan composition of H4N5S1.

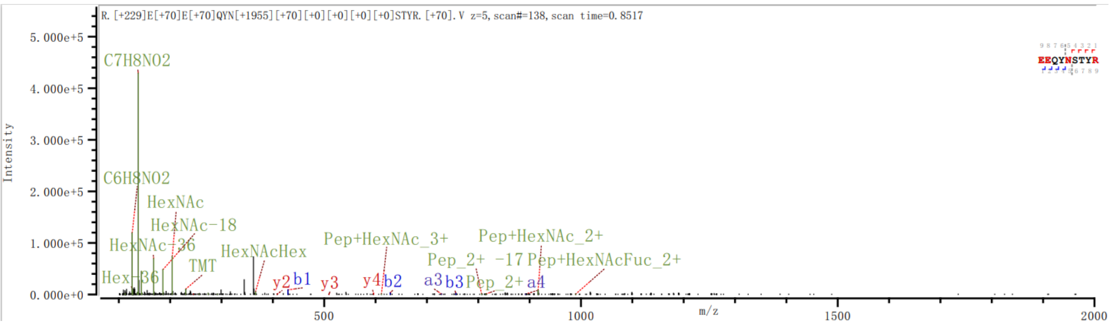

3521: EEQYNSTYR

#	a calc.	b calc.	b-18 calc.	b++ calc.	b_3+ calc.	Seq.	y calc.	y++ calc.	y_3+ calc.	#
1	401.3132	**429.3081**	411.2976	215.1577	143.7742	E				9
2	600.4448	**628.4397**	610.4291	314.7235	210.1514	E	3225.4401	1613.2237	1075.8182	8
3	**728.5034**	**756.4983**	738.4877	378.7528	252.8376	Q	3026.3085	1513.6579	1009.4410	7
4	891.5667	919.5616	901.5511	460.2844	307.1921	Y	2898.2499	1449.6286	966.7548	6
5	3030.4023	3058.3972	3040.3867	1529.7022	1020.1373	N	2735.1866	1368.0969	912.4004	5
6	3117.4343	3145.4292	3127.4187	1573.2183	1049.1479	S	**596.3510**	298.6791	199.4552	4
7	3218.4820	3246.4769	3228.4664	1623.7421	1082.8305	T	509.3190	255.1631	170.4445	3
8	3381.5453	3409.5403	3391.5297	1705.2738	1137.1849	Y	**408.2713**	204.6393	136.7619	2
9						R	245.2080	123.1076	82.4075	1

HCD MS2 spectrum of precursor m/z = 731.5514, z = 5^{+} at 0.8517 min. This spectrum was assigned to the EEQYNSTYR with N-glycan composition of H4N5S1.

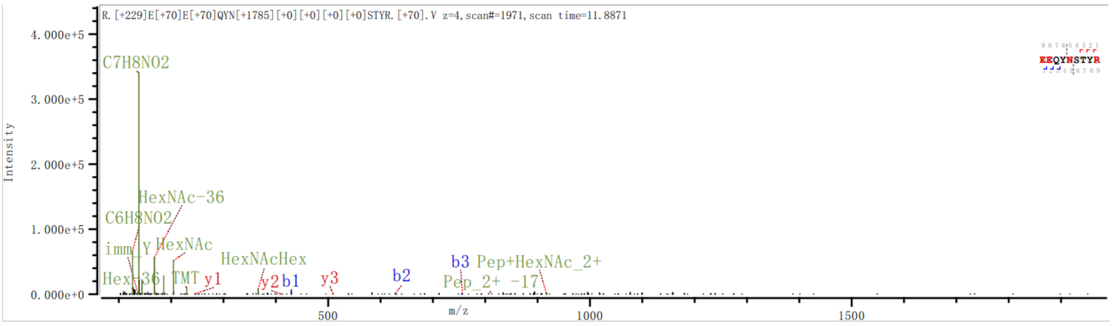

3085: EEQYNSTYR

#	a calc.	b calc.	b-18 calc.	b++ calc.	Seq.	y calc.	y++ calc.	y_3+ calc.	#
1	401.3132	**429.3081**	411.2976	215.1577	E				9
2	600.4448	**628.4397**	610.4291	314.7235	E	2985.2819	1493.1446	995.7655	8
3	728.5034	**756.4983**	738.4877	378.7528	Q	2786.1503	1393.5788	929.3883	7
4	891.5667	919.5616	901.5511	460.2844	Y	2658.0917	1329.5495	886.7021	6
5	2790.2441	2818.2390	2800.2284	1409.6231	N	2495.0284	1248.0178	832.3476	5
6	2877.2761	2905.2710	2887.2604	1453.1391	S	596.3510	298.6791	199.4552	4
7	2978.3238	3006.3187	2988.3081	1503.6630	T	**509.3190**	255.1631	170.4445	3
8	3141.3871	3169.3820	3151.3715	1585.1946	Y	**408.2713**	204.6393	136.7619	2
9					R	245.2080	123.1076	82.4075	1

HCD MS2 spectrum of precursor m/z = 854.1529, z = 5+ at 11.8871 min. This spectrum was assigned to the EEQYNSTYR with N-glycan composition of H4N6.

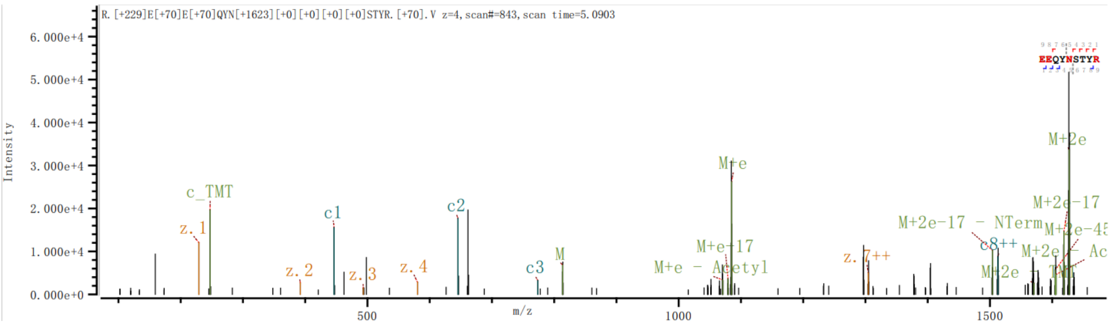

108: EEQYNSTYR

#	a. calc.	c calc.	c++ calc.	Seq.	y calc.	z. calc.	z.++ calc.	#
1	402.3210	**446.3347**	223.6710	E				9
2	601.4526	**645.4663**	323.2368	E	2823.2290	2807.2103	**1404.1088**	8
3	729.5112	**773.5248**	387.2661	Q	2624.0974	2608.0787	**1304.5430**	7
4	892.5745	936.5882	468.7977	Y	2496.0389	2480.0201	1240.5137	6
5	2629.1991	2673.2127	1337.1100	N	2332.9755	2316.9568	1158.9820	5
6	2716.2311	2760.2447	1380.6260	S	596.3510	**580.3323**		4
7	2817.2788	2861.2924	**1431.1498**	T	509.3190	493.3002		3
8	2980.3421	3024.3557	**1512.6815**	Y	408.2713	**392.2526**		2
9				R	245.2080	**229.1892**		1

ETD MS2 spectrum of precursor m/z = 813.6375, z = 4^{+} at 5.0903 min. This spectrum was assigned to the EEQYNSTYR with N-glycan composition of H5N4.

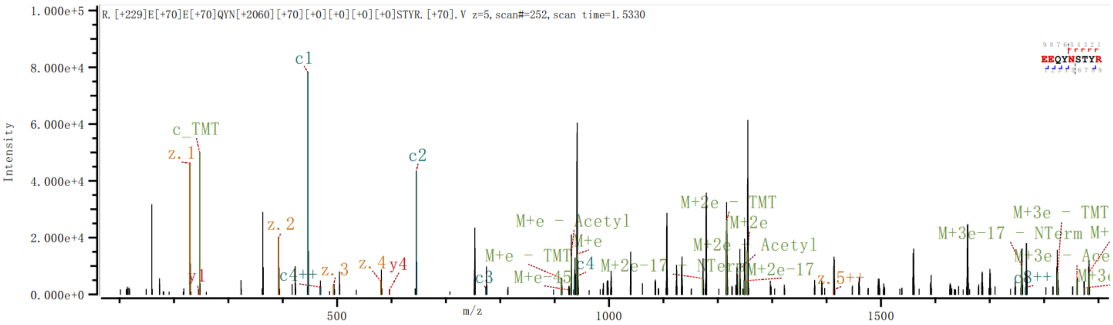

1289: EEQYNSTYR

#	a. calc.	c calc.	c++ calc.	Seq.	y calc.	z. calc.	z.++ calc.	#
1	402.3210	**446.3347**	223.6710	E				9
2	601.4526	**645.4663**	323.2368	E	3330.4715	3314.4527	1657.7300	8
3	729.5112	773.5248	387.2661	Q	3131.3399	3115.3211	1558.1642	7
4	892.5745	936.5882	468.7977	Y	3003.2813	2987.2626	1494.1349	6
5	3136.4415	3180.4551	1590.7312	N	2840.2180	2824.1992	1412.6033	5
6	3223.4735	3267.4872	1634.2472	S	596.3510	**580.3323**		4
7	3324.5212	3368.5348	1684.7711	T	509.3190	493.3002		3
8	3487.5845	3531.5982	1766.3027	Y	408.2713	**392.2526**		2
9				R	245.2080	**229.1892**		1

ETD MS2 spectrum of precursor m/z = 752.5551, z = 5^+ at 1.5330 min. This spectrum was assigned to the EEQYNSTYR with N-glycan composition of H5N4F1S1.

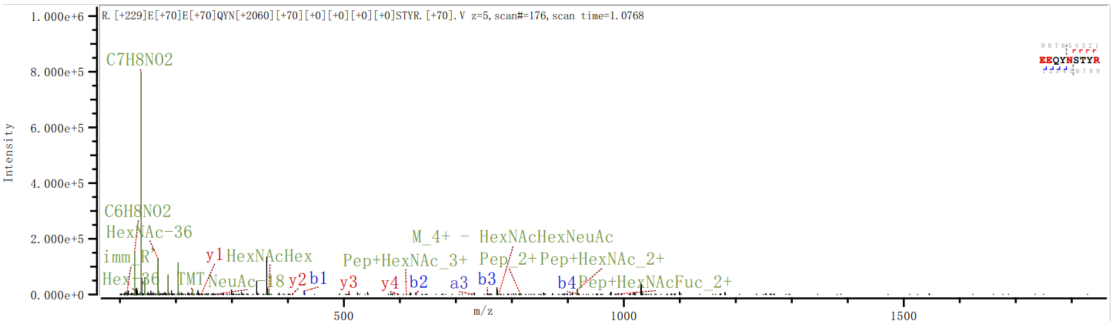

3524: EEQYNSTYR

#	a calc.	b calc.	b-18 calc.	b++ calc.	b_3+ calc.	Seq.	y calc.	y++ calc.	y_3+ calc.	#
1	401.3132	**429.3081**	411.2976	215.1577	143.7742	E				9
2	600.4448	**628.4397**	610.4291	314.7235	210.1514	E	3330.4715	1665.7394	1110.8287	8
3	728.5034	756.4983	738.4877	378.7528	252.8376	Q	3131.3399	1566.1736	1044.4515	7
4	891.5667	919.5616	901.5511	460.2844	307.1921	Y	3003.2813	1502.1443	1001.7653	6
5	3135.4337	3163.4286	3145.4180	1582.2179	1055.1477	N	2840.2180	1420.6126	947.4108	5
6	3222.4657	3250.4606	3232.4500	1625.7339	1084.1584	S	596.3510	298.6791	199.4552	4
7	3323.5134	3351.5083	3333.4977	1676.2578	1117.8409	T	**509.3190**	255.1631	170.4445	3
8	3486.5767	3514.5716	3496.5611	1757.7894	1172.1954	Y	**408.2713**	204.6393	136.7619	2
9						R	245.2080	123.1076	82.4075	1

HCD MS2 spectrum of precursor m/z = 752.5551, z = 5^+ at 1.0768 min. This spectrum was assigned to the EEQYNSTYR with N-glycan composition of H5N4F1S1.

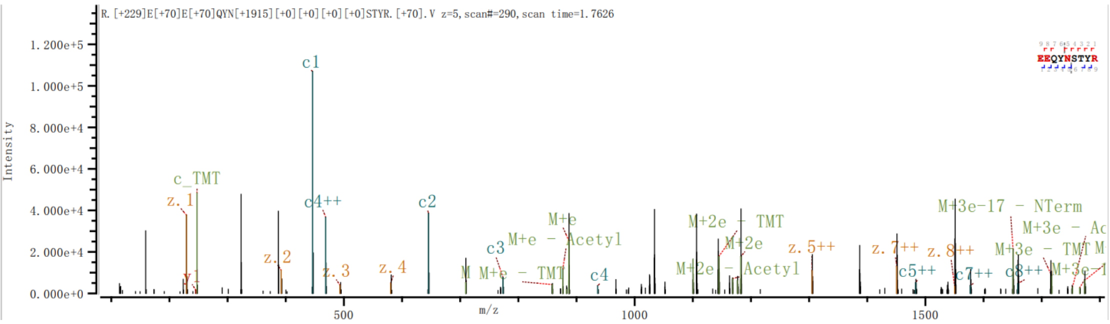

606: EEQYNSTYR

#	a. calc.	c calc.	c++ calc.	Seq.	y calc.	z. calc.	z.++ calc.	#
1	402.3210	**446.3347**	223.6710	E				9
2	601.4526	**645.4663**	323.2368	E	3115.3449	3099.3261	1550.1667	8
3	729.5112	**773.5248**	387.2661	Q	2916.2133	2900.1945	**1450.6009**	7
4	892.5745	**936.5882**	468.7977	Y	2788.1547	2772.1360	1386.5716	6
5	2921.3149	2965.3285	1483.1679	N	2625.0914	2609.0726	**1305.0400**	5
6	3008.3469	3052.3606	1526.6839	S	596.3510	**580.3323**		4
7	3109.3946	3153.4082	1577.2078	T	509.3190	493.3002		3
8	3272.4579	3316.4716	1658.7394	Y	408.2713	**392.2526**		2
9				R	245.2080	**229.1892**		1

ETD MS2 spectrum of precursor m/z = 709.5360, z = 5^+ at 1.7626 min. This spectrum was assigned to the EEQYNSTYR with N-glycan composition of H5N4F2.

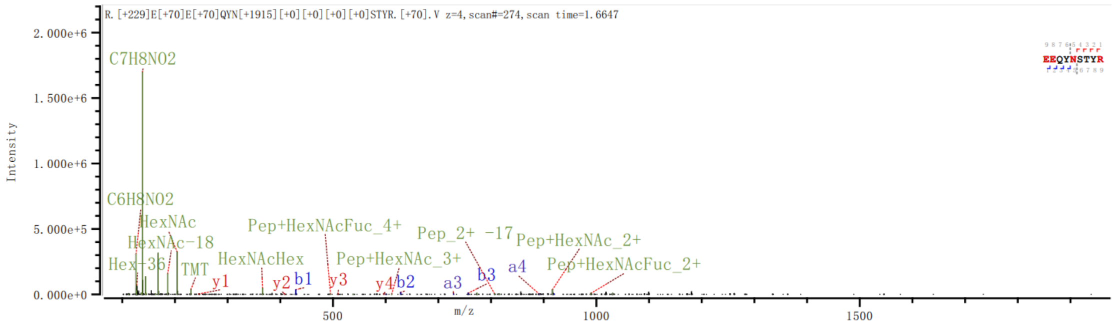

2851: EEQYNSTYR

#	a calc.	b calc.	b-18 calc.	b++ calc.	Seq.	y calc.	y++ calc.	y_3+ calc.	#
1	401.3132	**429.3081**	411.2976	215.1577	E				9
2	**600.4448**	**628.4397**	610.4291	314.7235	E	3115.3449	1558.1761	1039.1198	8
3	**728.5034**	**756.4983**	738.4877	378.7528	Q	2916.2133	1458.6103	972.7426	7
4	891.5667	919.5616	901.5511	460.2844	Y	2788.1547	1394.5810	930.0564	6
5	2920.3071	2948.3020	2930.2914	1474.6546	N	2625.0914	1313.0493	875.7020	5
6	3007.3391	3035.3340	3017.3234	1518.1706	S	**596.3510**	298.6791	199.4552	4
7	3108.3868	3136.3817	3118.3711	1568.6945	T	**509.3190**	255.1631	170.4445	3
8	3271.4501	3299.4450	3281.4345	1650.2261	Y	**408.2713**	204.6393	136.7619	2
9					R	**245.2080**	123.1076	82.4075	1

HCD MS2 spectrum of precursor m/z = 886.6656, z = 4^+ at 1.6647 min. This spectrum was assigned to the EEQYNSTYR with N-glycan composition of H5N4F2.

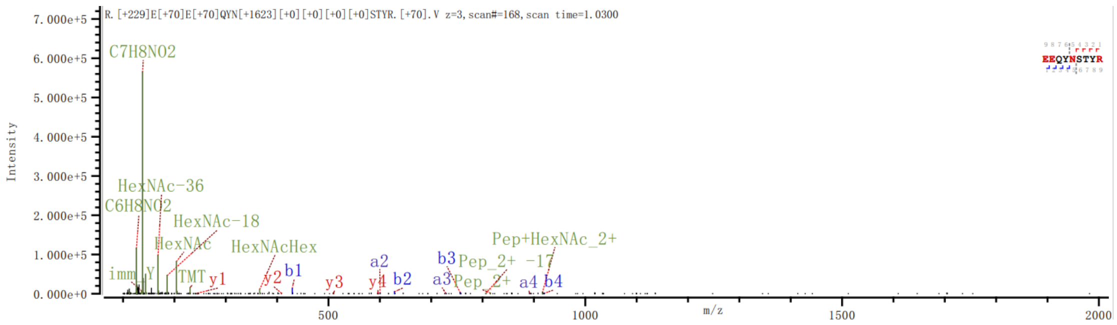

2255: EEQYNSTYR

#	a calc.	b calc.	b-18 calc.	b++ calc.	Seq.	y calc.	y++ calc.	#
1	401.3132	**429.3081**	411.2976	215.1577	E			9
2	**600.4448**	**628.4397**	610.4291	314.7235	E	2823.2290	1412.1182	8
3	**728.5034**	**756.4983**	738.4877	378.7528	Q	2624.0974	1312.5524	7
4	891.5667	**919.5616**	901.5511	460.2844	Y	2496.0389	1248.5231	6
5	2628.1912	2656.1862	2638.1756	1328.5967	N	2332.9755	1166.9914	5
6	2715.2233	2743.2182	2725.2076	1372.1127	S	**596.3510**	298.6791	4
7	2816.2710	2844.2659	2826.2553	1422.6366	T	**509.3190**	255.1631	3
8	2979.3343	3007.3292	2989.3186	1504.1682	Y	**408.2713**	204.6393	2
9					R	**245.2080**	123.1076	1

HCD MS2 spectrum of precursor m/z = 1084.5847, z = 3^+ at 1.0300 min. This spectrum was assigned to the EEQYNSTYR with N-glycan composition of H5N4.

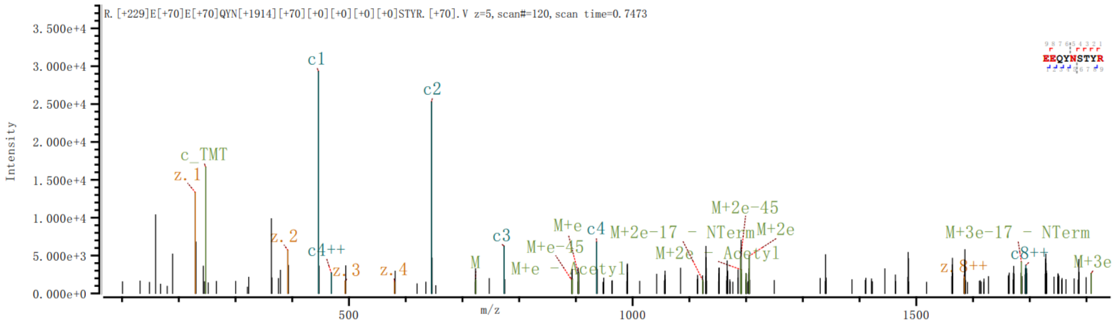

1269: EEQYNSTYR

#	a. calc.	c calc.	c++ calc.	Seq.	y calc.	z. calc.	z.++ calc.	#
1	402.3210	**446.3347**	223.6710	E				9
2	601.4526	**645.4663**	323.2368	E	3184.4135	3168.3948	1584.7010	8
3	729.5112	**773.5248**	387.2661	Q	2985.2820	2969.2632	1485.1353	7
4	892.5745	**936.5882**	468.7977	Y	2857.2234	2841.2046	1421.1060	6
5	2990.3836	3034.3972	1517.7022	N	2694.1600	2678.1413	1339.5743	5
6	3077.4156	3121.4292	1561.2183	S	596.3510	**580.3323**		4
7	3178.4633	3222.4769	1611.7421	T	509.3190	**493.3002**		3
8	3341.5266	3385.5403	**1693.2738**	Y	408.2713	**392.2526**		2
9				R	245.2080	**229.1892**		1

HCD MS2 spectrum of precursor m/z = 723.3478, z = 5^+ at 0.7473 min. This spectrum was assigned to the EEQYNSTYR with N-glycan composition of H5N4S1.

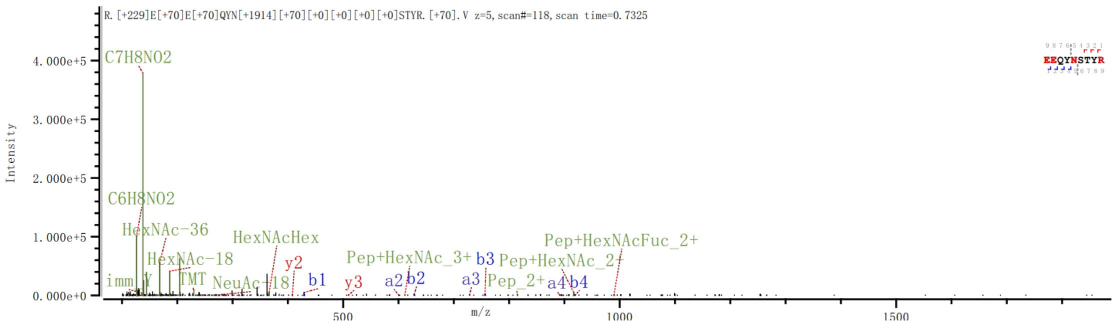

3518: EEQYNSTYR

#	a calc.	b calc.	b-18 calc.	b++ calc.	b_3+ calc.	Seq.	y calc.	y++ calc.	y_3+ calc.	#
1	401.3132	**429.3081**	411.2976	215.1577	143.7742	E				9
2	600.4448	**628.4397**	610.4291	314.7235	210.1514	E	3184.4135	1592.7104	1062.1427	8
3	**728.5034**	**756.4983**	738.4877	378.7528	252.8376	Q	2985.2820	1493.1446	995.7655	7
4	891.5667	919.5616	901.5511	460.2844	307.1921	Y	2857.2234	1429.1153	953.0793	6
5	2989.3758	3017.3707	2999.3601	1509.1890	1006.4617	N	2694.1600	1347.5837	898.7249	5
6	3076.4078	3104.4027	3086.3921	1552.7050	1035.4724	S	596.3510	298.6791	199.4552	4
7	3177.4555	3205.4504	3187.4398	1603.2288	1069.1550	T	**509.3190**	255.1631	170.4445	3
8	3340.5188	3368.5137	3350.5031	1684.7605	1123.5094	Y	**408.2713**	204.6393	136.7619	2
9						R	245.2080	123.1076	82.4075	1

HCD MS2 spectrum of precursor m/z = 723.3478, z = 5^+ at 0.7325 min. This spectrum was assigned to the EEQYNSTYR with N-glycan composition of H5N4S1.

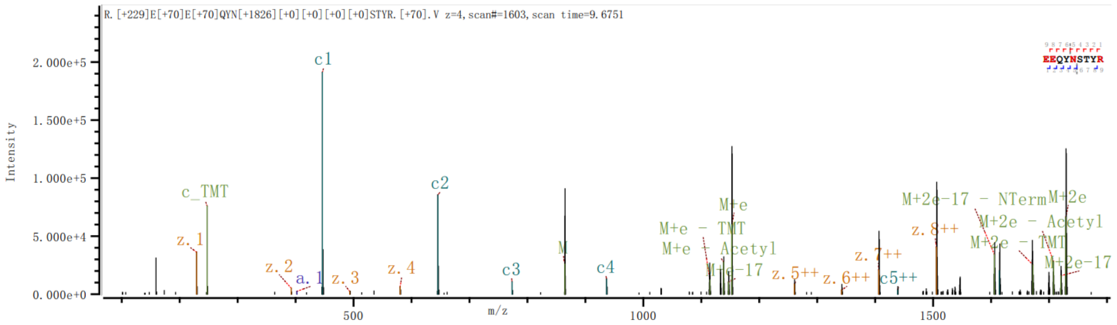

765: EEQYNSTYR

#	a. calc.	c calc.	c++ calc.	Seq.	y calc.	z. calc.	z.++ calc.	#
1	402.3210	**446.3347**	223.6710	E				9
2	601.4526	**645.4663**	323.2368	E	3026.3084	3010.2897	**1505.6485**	8
3	729.5112	**773.5248**	387.2661	Q	2827.1768	2811.1581	**1406.0827**	7
4	892.5745	**936.5882**	468.7977	Y	2699.1182	2683.0995	**1342.0534**	6
5	2832.2784	2876.2921	1438.6497	N	2536.0549	2520.0362	**1260.5217**	5
6	2919.3105	2963.3241	1482.1657	S	596.3510	**580.3323**		4
7	3020.3581	3064.3718	1532.6895	T	509.3190	493.3002		3
8	3183.4215	3227.4351	1614.2212	Y	408.2713	**392.2526**		2
9				R	245.2080	**229.1892**		1

ETD MS2 spectrum of precursor m/z = 864.4059, z = 4^+ at 9.6751 min. This spectrum was assigned to the EEQYNSTYR with N-glycan composition of H5N5.

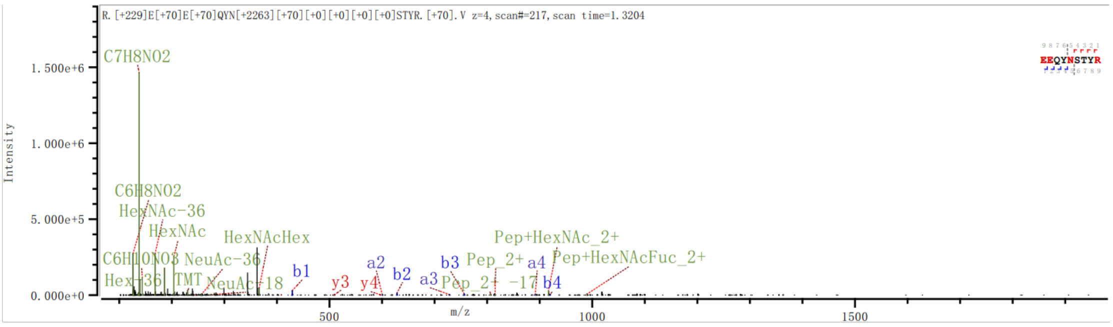

3944: EEQYNSTYR

#	a calc.	b calc.	b-18 calc.	b++ calc.	Seq.	y calc.	y++ calc.	y_3+ calc.	#
1	401.3132	**429.3081**	411.2976	215.1577	E				9
2	600.4448	**628.4397**	610.4291	314.7235	E	3533.5508	1767.2791	1178.5218	8
3	**728.5034**	**756.4983**	738.4877	378.7528	Q	3334.4192	1667.7133	1112.1446	7
4	891.5667	919.5616	901.5511	460.2844	Y	3206.3607	1603.6840	1069.4584	6
5	3338.5130	3366.5080	3348.4974	1683.7576	N	3043.2973	1522.1523	1015.1040	5
6	3425.5451	3453.5400	3435.5294	1727.2736	S	**596.3510**	298.6791	199.4552	4
7	3526.5928	3554.5877	3536.5771	1777.7975	T	**509.3190**	255.1631	170.4445	3
8	3689.6561	3717.6510	3699.6404	**1859.3291**	Y	**408.2713**	204.6393	136.7619	2
9					R	245.2080	123.1076	82.4075	1

HCD MS2 spectrum of precursor m/z = 991.2174, z = 4^+ at 1.3204 min. This spectrum was assigned to the EEQYNSTYR with N-glycan composition of H5N5F1S1.

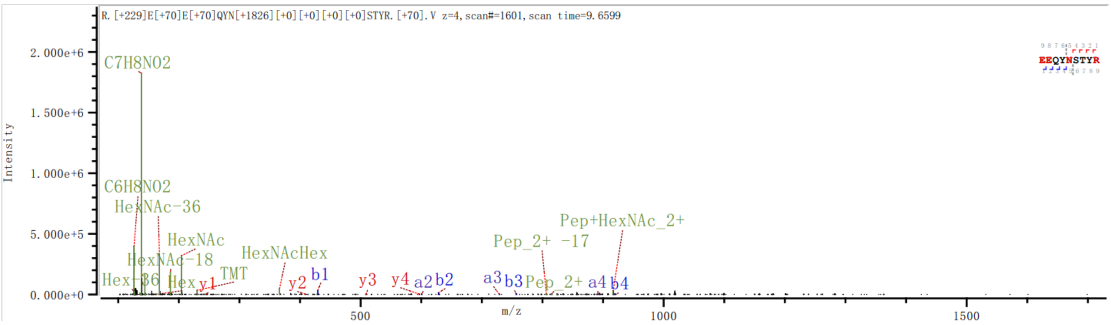

3014: EEQYNSTYR

#	a calc.	b calc.	b-18 calc.	b++ calc.	Seq.	y calc.	y++ calc.	y_3+ calc.	#
1	401.3132	**429.3081**	411.2976	215.1577	E				9
2	**600.4448**	**628.4397**	610.4291	314.7235	E	3026.3084	1513.6578	1009.4410	8
3	**728.5034**	**756.4983**	738.4877	378.7528	Q	2827.1768	1414.0920	943.0638	7
4	891.5667	919.5616	901.5511	460.2844	Y	2699.1182	1350.0628	900.3776	6
5	2831.2706	2859.2655	2841.2550	1430.1364	N	2536.0549	1268.5311	846.0232	5
6	2918.3026	2946.2976	2928.2870	1473.6524	S	**596.3510**	298.6791	199.4552	4
7	3019.3503	3047.3452	3029.3347	1524.1763	T	**509.3190**	255.1631	170.4445	3
8	3182.4137	3210.4086	3192.3980	1605.7079	Y	**408.2713**	204.6393	136.7619	2
9					R	**245.2080**	123.1076	82.4075	1

HCD MS2 spectrum of precursor m/z = 864.4059, z = 4^+ at 9.6599 min. This spectrum was assigned to the EEQYNSTYR with N-glycan composition of H5N5.

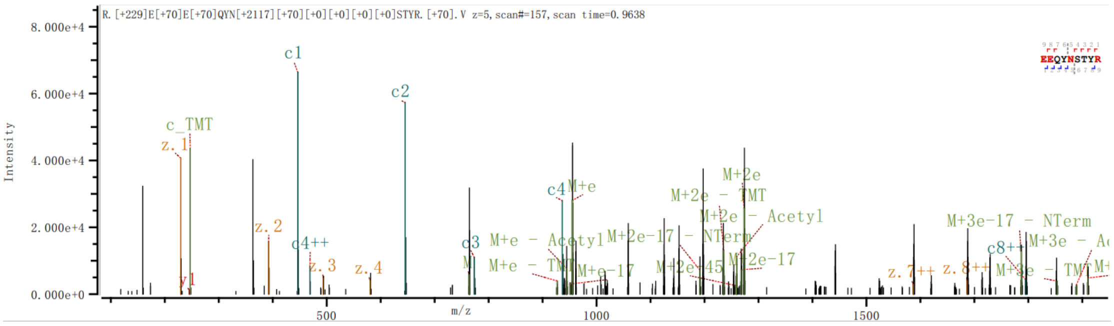

1273: EEQYNSTYR

#	a. calc.	c calc.	c++ calc.	Seq.	y calc.	z. calc.	z.++ calc.	#
1	402.3210	**446.3347**	223.6710	E				9
2	601.4526	**645.4663**	323.2368	E	3387.4929	3371.4742	1686.2407	8
3	729.5112	**773.5248**	387.2661	Q	3188.3613	3172.3426	1586.6749	7
4	892.5745	**936.5882**	468.7977	Y	3060.3028	3044.2840	1522.6457	6
5	3193.4630	3237.4766	1619.2419	N	2897.2394	2881.2207	1441.1140	5
6	3280.4950	3324.5086	1662.7580	S	596.3510	**580.3323**		4
7	3381.5427	3425.5563	1713.2818	T	509.3190	**493.3002**		3
8	3544.6060	3588.6196	1794.8135	Y	408.2713	**392.2526**		2
9				R	245.2080	**229.1892**		1

ETD MS2 spectrum of precursor m/z = 763.9627, z = 5^+ at 0.9638 min. This spectrum was assigned to the EEQYNSTYR with N-glycan composition of H5N5S1.

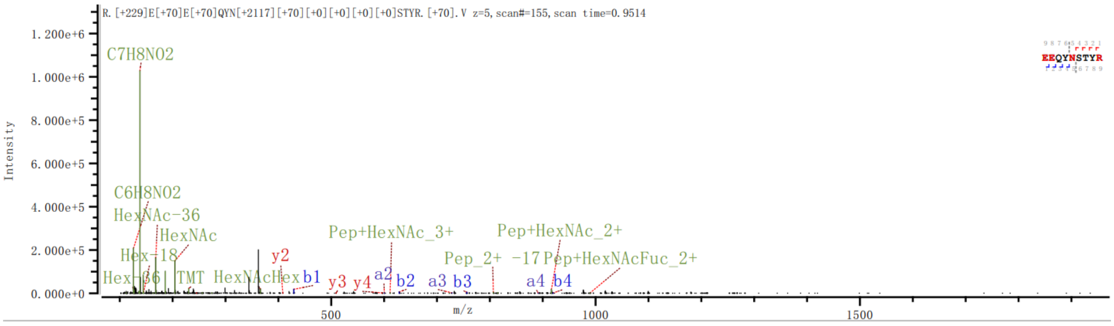

3522: EEQYNSTYR

#	a calc.	b calc.	b-18 calc.	b++ calc.	b_3+ calc.	Seq.	y calc.	y++ calc.	y_3+ calc.	#
1	401.3132	**429.3081**	411.2976	215.1577	143.7742	E				9
2	**600.4448**	**628.4397**	610.4291	314.7235	210.1514	E	3387.4929	1694.2501	1129.8358	8
3	728.5034	**756.4983**	738.4877	378.7528	252.8376	Q	3188.3613	1594.6843	1063.4586	7
4	891.5667	919.5616	901.5511	460.2844	307.1921	Y	3060.3028	1530.6550	1020.7724	6
5	3192.4551	3220.4500	3202.4395	1610.7287	1074.1549	N	2897.2394	1449.1233	966.4180	5
6	3279.4872	3307.4821	3289.4715	1654.2447	1103.1655	S	**596.3510**	298.6791	199.4552	4
7	3380.5348	3408.5298	3390.5192	1704.7685	1136.8481	T	509.3190	255.1631	170.4445	3
8	3543.5982	3571.5931	3553.5825	1786.3002	1191.2025	Y	408.2713	204.6393	136.7619	2
9						R	245.2080	123.1076	82.4075	1

HCD MS2 spectrum of precursor m/z = 763.9627, z = 5^+ at 0.9514 min. This spectrum was assigned to the EEQYNSTYR with N-glycan composition of H5N5S1.

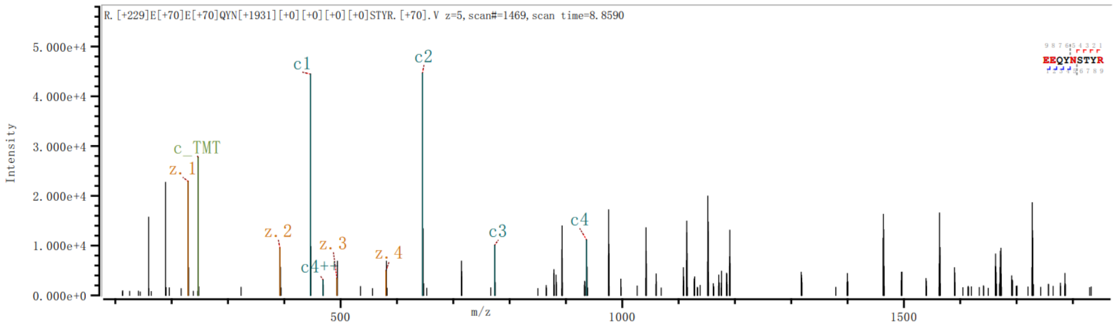

729: EEQYNSTYR

#	a. calc.	c calc.	c++ calc.	Seq.	y calc.	z. calc.	z.++ calc.	#
1	402.3210	**446.3347**	223.6710	E				9
2	601.4526	**645.4663**	323.2368	E	3131.3398	3115.3210	1558.1642	8
3	729.5112	**773.5248**	387.2661	Q	2932.2082	2916.1894	1458.5984	7
4	892.5745	**936.5882**	468.7977	Y	2804.1496	2788.1309	1394.5691	6
5	2937.3098	2981.3234	1491.1654	N	2641.0863	2625.0675	1313.0374	5
6	3024.3418	3068.3555	1534.6814	S	596.3510	**580.3323**		4
7	3125.3895	3169.4031	1585.2052	T	509.3190	**493.3002**		3
8	3288.4528	3332.4665	1666.7369	Y	408.2713	**392.2526**		2
9				R	245.2080	**229.1892**		1

ETD MS2 spectrum of precursor m/z = 712.7339, z = 5^+ at 8.8590 min. This spectrum was assigned to the EEQYNSTYR with N-glycan composition of H6N4F1.

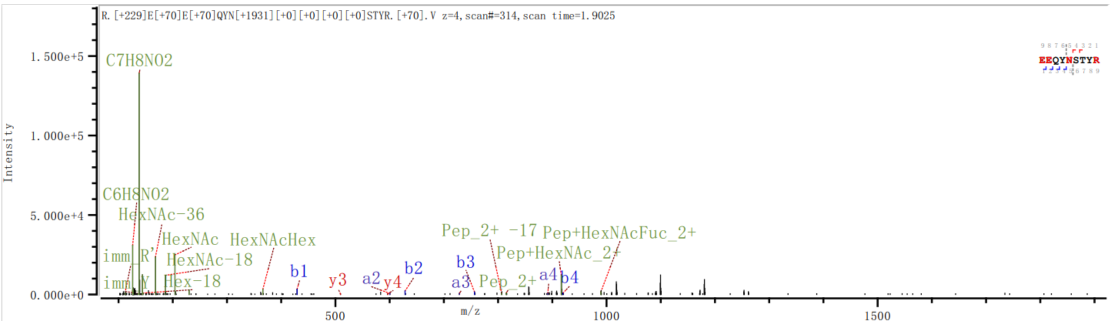

2863: EEQYNSTYR

#	a calc.	b calc.	b-18 calc.	b++ calc.	Seq.	y calc.	y++ calc.	y_3+ calc.	#
1	401.3132	**429.3081**	411.2976	215.1577	E				9
2	**600.4448**	**628.4397**	610.4291	314.7235	E	3131.3398	1566.1735	1044.4514	8
3	**728.5034**	**756.4983**	738.4877	378.7528	Q	2932.2082	1466.6077	978.0742	7
4	891.5667	919.5616	901.5511	460.2844	Y	2804.1496	1402.5784	935.3880	6
5	2936.3020	2964.2969	2946.2863	1482.6521	N	2641.0863	1321.0468	881.0336	5
6	3023.3340	3051.3289	3033.3184	1526.1681	S	**596.3510**	298.6791	199.4552	4
7	3124.3817	3152.3766	3134.3660	1576.6919	T	**509.3190**	255.1631	170.4445	3
8	3287.4450	3315.4399	3297.4294	1658.2236	Y	408.2713	204.6393	136.7619	2
9					R	245.2080	123.1076	82.4075	1

HCD MS2 spectrum of precursor m/z = 890.6651, z = 4^{+} at 1.9025 min. This spectrum was assigned to the EEQYNSTYR with N-glycan composition of H6N4F1.

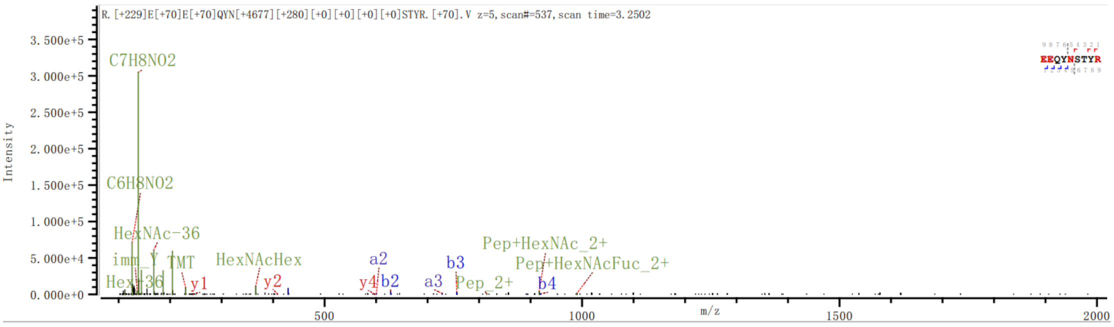

4182: EEQYNSTYR

#	a calc.	b calc.	b-18 calc.	b++ calc.	b_3+ calc.	Seq.	y calc.	y++ calc.	y_3+ calc.	#
1	401.3132	**429.3081**	411.2976	215.1577	143.7742	E				9
2	**600.4448**	**628.4397**	610.4291	314.7235	210.1514	E	6157.6329	3079.3201	2053.2158	8
3	**728.5034**	**756.4983**	738.4877	378.7528	252.8376	Q	5958.5013	2979.7543	1986.8386	7
4	891.5667	919.5616	901.5511	460.2844	307.1921	Y	5830.4427	2915.7250	1944.1524	6
5	5962.5951	5990.5900	5972.5795	2995.7986	1997.5349	N	5667.3794	2834.1933	1889.7980	5
6	6049.6271	6077.6220	6059.6115	3039.3147	2026.5455	S	**596.3510**	298.6791	199.4552	4
7	6150.6748	6178.6697	6160.6592	3089.8385	2060.2281	T	509.3190	255.1631	170.4445	3
8	6313.7381	6341.7331	6323.7225	3171.3702	2114.5825	Y	**408.2713**	204.6393	136.7619	2
9						R	**245.2080**	123.1076	82.4075	1

HCD MS2 spectrum of precursor m/z = 1317.9907, z = 5^+ at 3.2502 min. This spectrum was assigned to the EEQYNSTYR with N-glycan composition of H12N7F1S4.

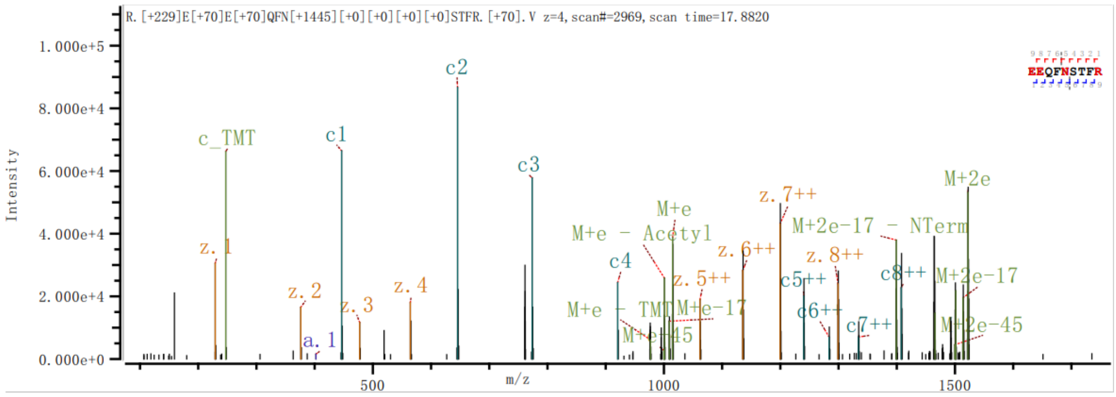

422: EEQFNSTFR

#	a. calc.	c calc.	c++ calc.	Seq.	y calc.	z. calc.	z.++ calc.	#
1	402.3210	**446.3347**	223.6710	E				9
2	601.4526	**645.4663**	323.2368	E	2613.1915	2597.1727	**1299.0900**	8
3	729.5112	**773.5248**	387.2661	Q	2414.0599	2398.0411	**1199.5242**	7
4	876.5796	**920.5932**	460.8003	F	2286.0013	2269.9826	**1135.4949**	6
5	2435.1564	2479.1700	1240.0887	N	2138.9329	2122.9141	**1061.9607**	5
6	2522.1884	2566.2021	1283.6047	S	580.3561	**564.3373**		4
7	2623.2361	2667.2498	1334.1285	T	493.3240	**477.3053**		3
8	2770.3045	2814.3182	**1407.6627**	F	392.2764	**376.2576**		2
9				R	245.2080	**229.1892**		1

ETD MS2 spectrum of precursor m/z = 761.1280, z = 4^{+} at 17.8820 min. This spectrum was assigned to the EEQFNSTFR with N-glycan composition of H3N4F1.

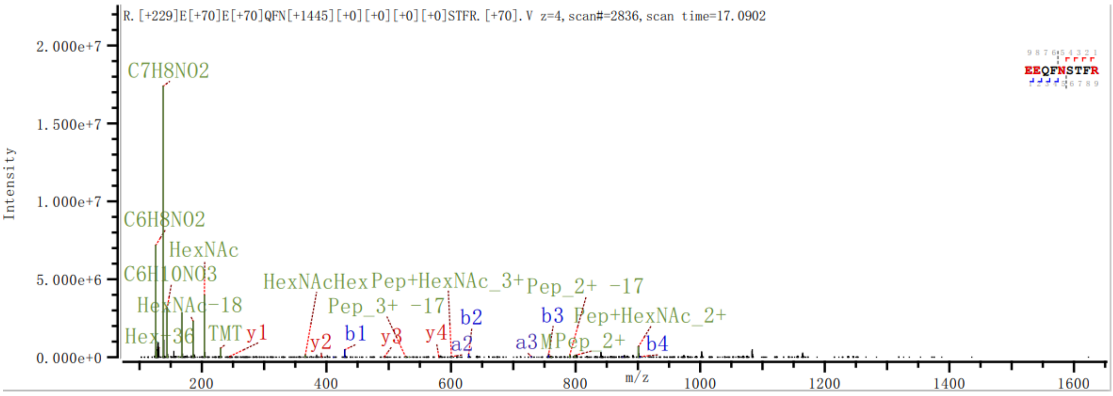

2663: EEQFNSTFR

#	a calc.	b calc.	b-18 calc.	b++ calc.	Seq.	y calc.	y++ calc.	y_3+ calc.	#
1	401.3132	429.3081	411.2976	215.1577	E				9
2	600.4448	628.4397	610.4291	314.7235	E	2613.1915	1307.0994	871.7353	8
3	728.5034	756.4983	738.4877	378.7528	Q	2414.0599	1207.5336	805.3581	7
4	875.5718	903.5667	885.5561	452.2870	F	2286.0013	1143.5043	762.6719	6
5	2434.1486	2462.1435	2444.1329	1231.5754	N	2138.9329	1069.9701	713.6491	5
6	2521.1806	2549.1755	2531.1650	1275.0914	S	580.3561	290.6817	194.1235	4
7	2622.2283	2650.2232	2632.2126	1325.6152	T	493.3240	247.1657	165.1129	3
8	2769.2967	2797.2916	2779.2811	1399.1494	F	392.2764	196.6418	131.4303	2
9					R	245.2080	123.1076	82.4075	1

HCD MS2 spectrum of precursor m/z = 761.1271, z = 4^+ at 17.0902 min. This spectrum was assigned to the EEQFNSTFR with N-glycan composition of H3N4F1.

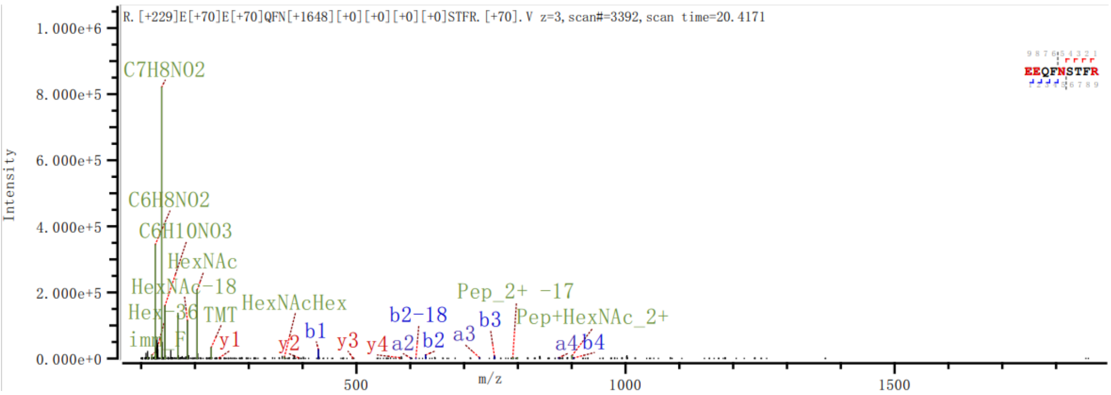

2681: EEQFNSTFR

#	a calc.	b calc.	b-18 calc.	b++ calc.	Seq.	y calc.	y++ calc.	#
1	401.3132	**429.3081**	411.2976	215.1577	E			9
2	**600.4448**	**628.4397**	610.4291	314.7235	E	2816.2708	1408.6390	8
3	**728.5034**	**756.4983**	738.4877	378.7528	Q	2617.1392	1309.0733	7
4	**875.5718**	**903.5667**	885.5561	452.2870	F	2489.0807	1245.0440	6
5	2637.2280	2665.2229	2647.2123	1333.1151	N	2342.0122	1171.5098	5
6	2724.2600	2752.2549	2734.2443	1376.6311	S	**580.3561**	290.6817	4
7	2825.3077	2853.3026	2835.2920	1427.1549	T	**493.3240**	247.1657	3
8	2972.3761	3000.3710	2982.3604	1500.6891	F	**392.2764**	196.6418	2
9					R	**245.2080**	123.1076	1

HCD MS2 spectrum of precursor m/z = 1082.1949, z = 3^+ at 20.4171 min. This spectrum was assigned to the EEQFNSTFR with N-glycan composition of H3N5F1.

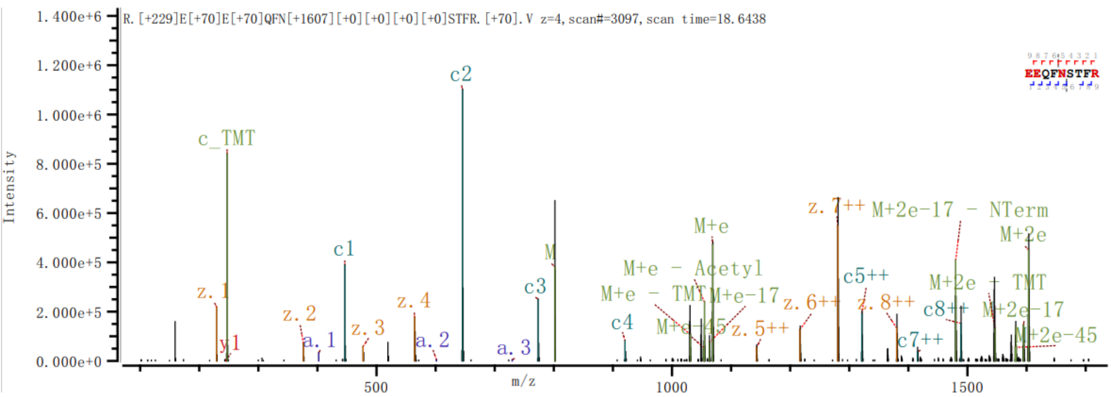

426: EEQFNSTFR

#	a. calc.	c calc.	c++ calc.	Seq.	y calc.	z. calc.	z.++ calc.	#
1	**402.3210**	**446.3347**	223.6710	E				9
2	**601.4526**	**645.4663**	323.2368	E	2775.2443	2759.2255	**1380.1164**	8
3	**729.5112**	**773.5248**	387.2661	Q	2576.1127	2560.0940	**1280.5506**	7
4	876.5796	**920.5932**	460.8003	F	2448.0541	2432.0354	1216.5213	6
5	2597.2092	2641.2229	**1321.1151**	N	2300.9857	2284.9670	**1142.9871**	5
6	2684.2413	2728.2549	1364.6311	S	580.3561	**564.3373**		4
7	2785.2889	2829.3026	1415.1549	T	493.3240	**477.3053**		3
8	2932.3573	2976.3710	**1488.6891**	F	392.2764	**376.2576**		2
9				R	245.2080	**229.1892**		1

ETD MS2 spectrum of precursor m/z = 801.6419, z = 4^+ at 18.6438 min. This spectrum was assigned to the EEQFNSTFR with N-glycan composition of H4N4F1.

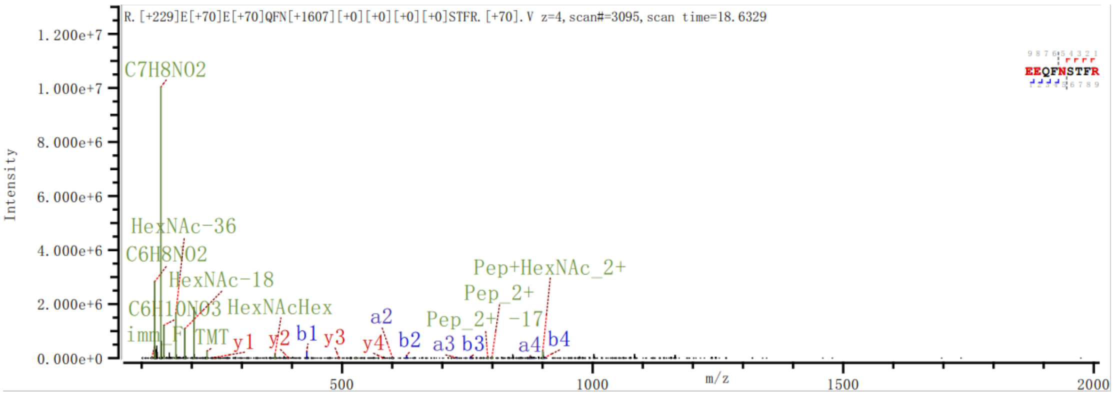

2675: EEQFNSTFR

#	a calc.	b calc.	b-18 calc.	b++ calc.	Seq.	y calc.	y++ calc.	y_3+ calc.	#
1	401.3132	**429.3081**	411.2976	215.1577	E				9
2	**600.4448**	**628.4397**	610.4291	314.7235	E	2775.2443	1388.1258	925.7529	8
3	728.5034	**756.4983**	738.4877	378.7528	Q	2576.1127	1288.5600	859.3757	7
4	875.5718	903.5667	885.5561	452.2870	F	2448.0541	1224.5307	816.6896	6
5	2596.2014	2624.1963	2606.1858	1312.6018	N	2300.9857	1150.9965	767.6667	5
6	2683.2334	2711.2283	2693.2178	1356.1178	S	**580.3561**	290.6817	194.1235	4
7	2784.2811	2812.2760	2794.2655	1406.6417	T	**493.3240**	247.1657	165.1129	3
8	2931.3495	2959.3444	2941.3339	1480.1759	F	**392.2764**	196.6418	131.4303	2
9					R	**245.2080**	123.1076	82.4075	1

HCD MS2 spectrum of precursor m/z = 801.6419, z = 4^{+} at 18.6329 min. This spectrum was assigned to the EEQFNSTFR with N-glycan composition of H4N4F1.

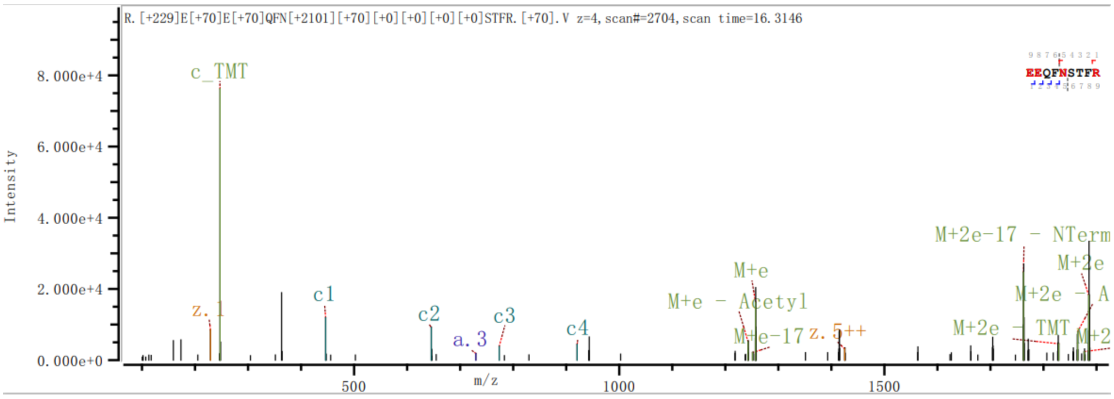

1406: EEQFNSTFR

#	a. calc.	c calc.	c++ calc.	Seq.	y calc.	z. calc.	z.++ calc.	#
1	402.3210	**446.3347**	223.6710	E				9
2	601.4526	**645.4663**	323.2368	E	3339.5082	3323.4894	1662.2484	8
3	**729.5112**	**773.5248**	387.2661	Q	3140.3766	3124.3578	**1562.6826**	7
4	876.5796	**920.5932**	460.8003	F	3012.3180	2996.2993	1498.6533	6
5	3161.4731	3205.4868	1603.2470	N	2865.2496	2849.2309	**1425.1191**	5
6	3248.5051	3292.5188	1646.7630	S	580.3561	564.3373		4
7	3349.5528	3393.5665	1697.2869	T	493.3240	477.3053		3
8	3496.6212	3540.6349	1770.8211	F	392.2764	376.2576		2
9				R	245.2080	**229.1892**		1

ETD MS2 spectrum of precursor m/z = 742.7084, z = 4^{+} at 16.3146 min. This spectrum was assigned to the EEQFNSTFR with N-glycan composition of H4N5F1S1.

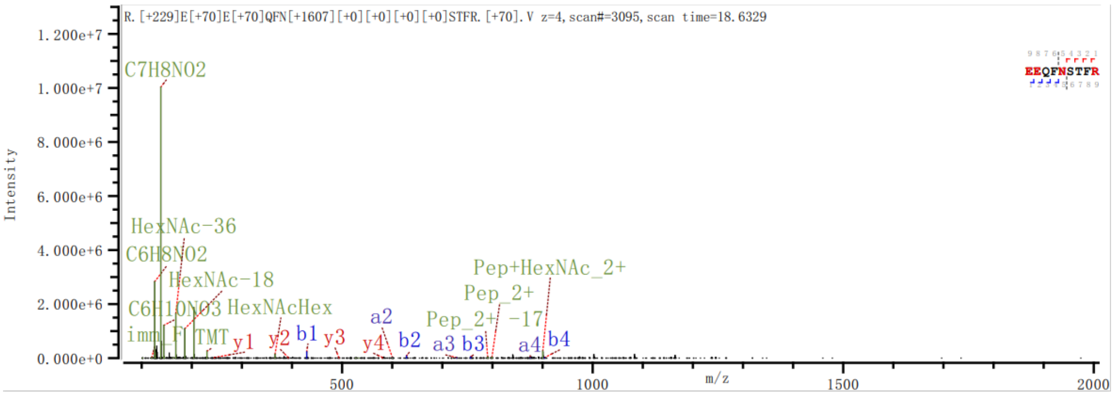

2675: EEQFNSTFR

#	a calc.	b calc.	b-18 calc.	b++ calc.	Seq.	y calc.	y++ calc.	y_3+ calc.	#
1	401.3132	**429.3081**	411.2976	215.1577	E				9
2	**600.4448**	**628.4397**	610.4291	314.7235	E	2775.2443	1388.1258	925.7529	8
3	728.5034	**756.4983**	738.4877	378.7528	Q	2576.1127	1288.5600	859.3757	7
4	875.5718	903.5667	885.5561	452.2870	F	2448.0541	1224.5307	816.6896	6
5	2596.2014	2624.1963	2606.1858	1312.6018	N	2300.9857	1150.9965	767.6667	5
6	2683.2334	2711.2283	2693.2178	1356.1178	S	**580.3561**	290.6817	194.1235	4
7	2784.2811	2812.2760	2794.2655	1406.6417	T	**493.3240**	247.1657	165.1129	3
8	2931.3495	2959.3444	2941.3339	1480.1759	F	**392.2764**	196.6418	131.4303	2
9					R	**245.2080**	123.1076	82.4075	1

HCD MS2 spectrum of precursor m/z = 801.6419, z = 4^+ at 18.6329 min. This spectrum was assigned to the EEQFNSTFR with N-glycan composition of H4N4F1.

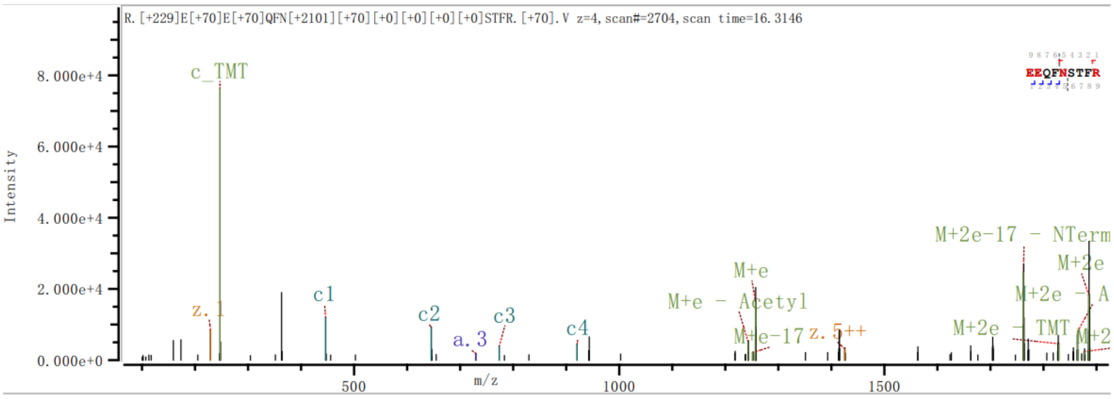

1406: EEQFNSTFR

#	a. calc.	c calc.	c++ calc.	Seq.	y calc.	z. calc.	z.++ calc.	#
1	402.3210	**446.3347**	223.6710	E				9
2	601.4526	**645.4663**	323.2368	E	3339.5082	3323.4894	1662.2484	8
3	**729.5112**	**773.5248**	387.2661	Q	3140.3766	3124.3578	**1562.6826**	7
4	876.5796	**920.5932**	460.8003	F	3012.3180	2996.2993	1498.6533	6
5	3161.4731	3205.4868	1603.2470	N	2865.2496	2849.2309	**1425.1191**	5
6	3248.5051	3292.5188	1646.7630	S	580.3561	564.3373		4
7	3349.5528	3393.5665	1697.2869	T	493.3240	477.3053		3
8	3496.6212	3540.6349	1770.8211	F	392.2764	376.2576		2
9				R	245.2080	**229.1892**		1

ETD MS2 spectrum of precursor m/z = 742.7084, z = 4^{+} at 16.3146 min. This spectrum was assigned to the EEQFNSTFR with N-glycan composition of H4N5F1S1.

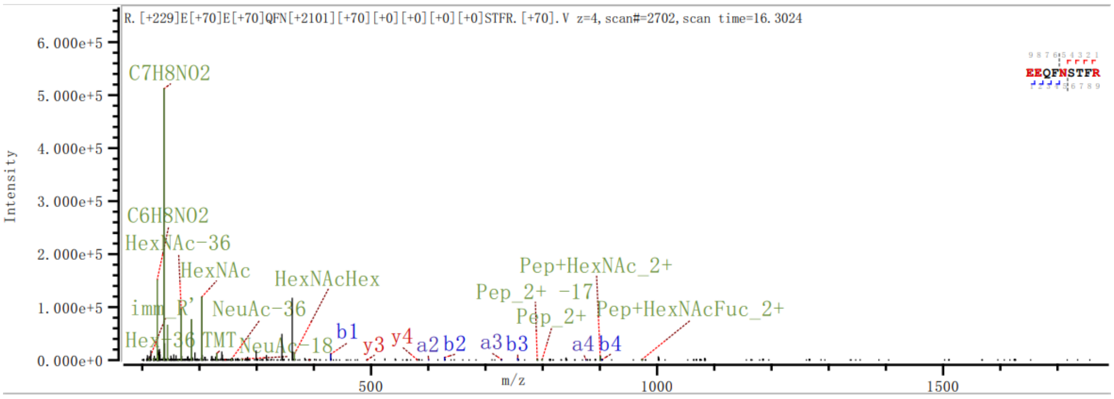

3655: EEQFNSTFR

#	a calc.	b calc.	b-18 calc.	b++ calc.	Seq.	y calc.	y++ calc.	y_3+ calc.	#
1	401.3132	**429.3081**	411.2976	215.1577	E				9
2	**600.4448**	**628.4397**	610.4291	314.7235	E	3339.5082	1670.2577	1113.8409	8
3	**728.5034**	**756.4983**	738.4877	378.7528	Q	3140.3766	1570.6919	1047.4637	7
4	875.5718	903.5667	885.5561	452.2870	F	3012.3180	1506.6626	1004.7775	6
5	3160.4653	3188.4602	3170.4496	1594.7337	N	2865.2496	1433.1284	955.7547	5
6	3247.4973	3275.4922	3257.4817	1638.2498	S	**580.3561**	290.6817	194.1235	4
7	3348.5450	3376.5399	3358.5294	1688.7736	T	**493.3240**	247.1657	165.1129	3
8	3495.6134	3523.6083	3505.5978	1762.3078	F	392.2764	196.6418	131.4303	2
9					R	245.2080	123.1076	82.4075	1

HCD MS2 spectrum of precursor m/z = 942.7084, z = 4^+ at 16.3024 min. This spectrum was assigned to the EEQFNSTFR with N-glycan composition of H4N5F1S1.

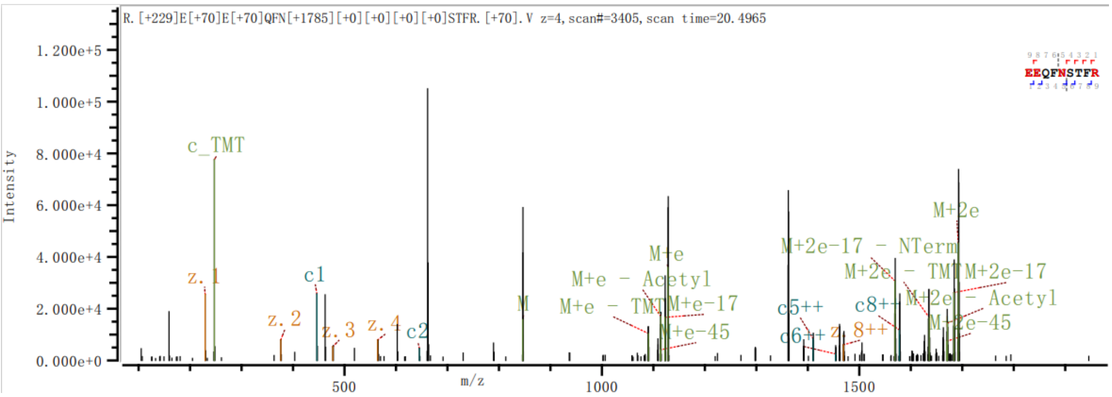

1020: EEQFNSTFR

#	a. calc.	c calc.	c++ calc.	Seq.	y calc.	z. calc.	z.++ calc.	#
1	402.3210	**446.3347**	223.6710	E				9
2	601.4526	**645.4663**	323.2368	E	2953.2920	2937.2733	1469.1403	8
3	729.5112	773.5248	387.2661	Q	2754.1604	2738.1417	1369.5745	7
4	876.5796	920.5932	460.8003	F	2626.1018	2610.0831	1305.5452	6
5	2775.2570	2819.2706	1410.1389	N	2479.0334	2463.0147	1232.0110	5
6	2862.2890	2906.3026	1453.6550	S	580.3561	**564.3373**		4
7	2963.3367	3007.3503	1504.1788	T	493.3240	**477.3053**		3
8	3110.4051	3154.4187	1577.7130	F	392.2764	**376.2576**		2
9				R	245.2080	**229.1892**		1

ETD MS2 spectrum of precursor m/z = 846.1519, z = 4^+ at 20.4965 min. This spectrum was assigned to the EEQFNSTFR with N-glycan composition of H4N6.

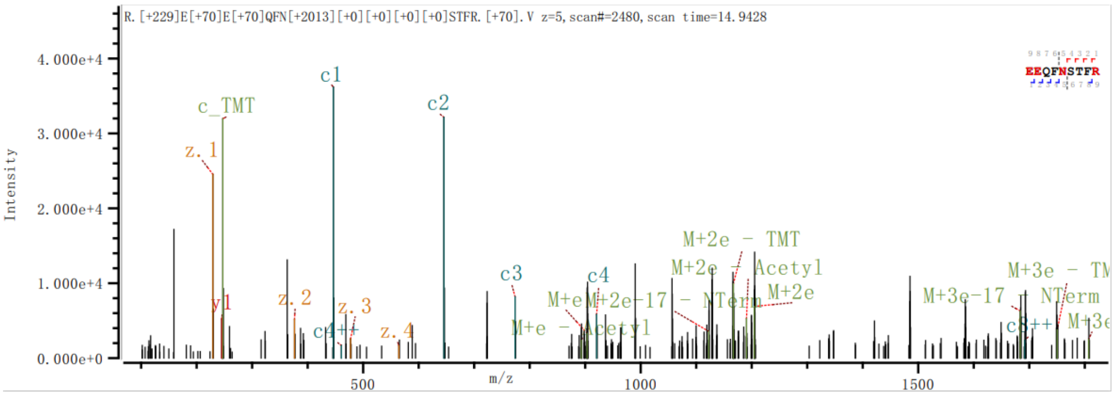

1396: EEQFNSTFR

#	a. calc.	c calc.	c++ calc.	Seq.	y calc.	z. calc.	z.++ calc.	#
1	402.3210	**446.3347**	223.6710	E				9
2	601.4526	**645.4663**	323.2368	E	3181.4030	3165.3843	1583.1958	8
3	729.5112	**773.5248**	387.2661	Q	2982.2714	2966.2527	1483.6300	7
4	876.5796	920.5932	460.8003	F	2854.2129	2838.1941	1419.6007	6
5	3003.3680	3047.3816	1524.1944	N	2707.1444	2691.1257	1346.0665	5
6	3090.4000	3134.4136	1567.7105	S	580.3561	**564.3373**		4
7	3191.4477	3235.4613	1618.2343	T	493.3240	**477.3053**		3
8	3338.5161	3382.5297	1691.7685	F	392.2764	**376.2576**		2
9				R	**245.2080**	**229.1892**		1

ETD MS2 spectrum of precursor m/z = 722.7534, z = 5^+ at 14.9428 min. This spectrum was assigned to the EEQFNSTFR with N-glycan composition of H4N6F1.

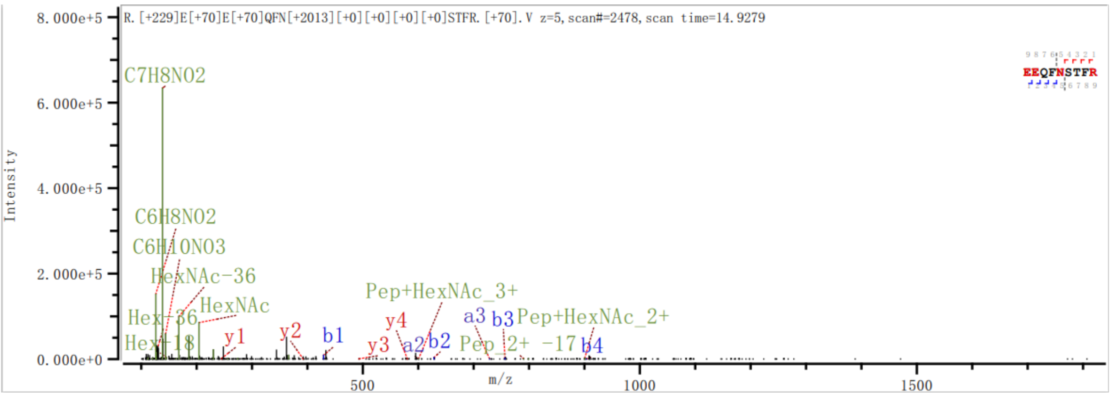

3645: EEQFNSTFR

#	a calc.	b calc.	b-18 calc.	b++ calc.	b_3+ calc.	Seq.	y calc.	y++ calc.	y_3+ calc.	#
1	401.3132	**429.3081**	411.2976	215.1577	143.7742	E				9
2	600.4448	**628.4397**	610.4291	314.7235	210.1514	E	3181.4030	1591.2051	1061.1392	8
3	**728.5034**	**756.4983**	738.4877	378.7528	252.8376	Q	2982.2714	1491.6394	994.7620	7
4	875.5718	903.5667	885.5561	452.2870	301.8604	F	2854.2129	1427.6101	952.0758	6
5	3002.3602	3030.3551	3012.3445	1515.6812	1010.7899	N	2707.1444	1354.0759	903.0530	5
6	3089.3922	3117.3871	3099.3765	1559.1972	1039.8005	S	580.3561	290.6817	194.1235	4
7	3190.4399	3218.4348	3200.4242	1609.7210	1073.4831	T	493.3240	247.1657	165.1129	3
8	3337.5083	3365.5032	3347.4926	1683.2552	1122.5059	F	392.2764	196.6418	131.4303	2
9						R	245.2080	123.1076	82.4075	1

HCD MS2 spectrum of precursor m/z = 722.7534, z = 5^+ at 14.9428 min. This spectrum was assigned to the EEQFNSTFR with N-glycan composition of H4N6F1.

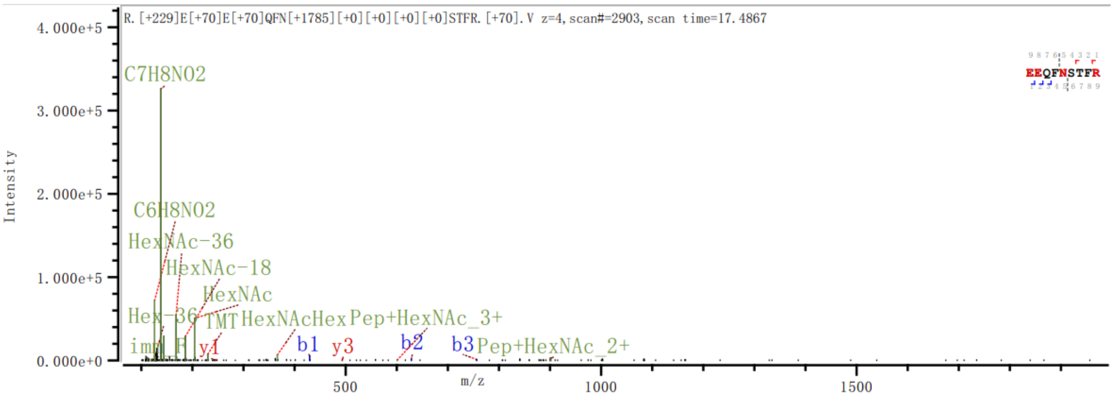

3214: EEQFNSTFR

#	a calc.	b calc.	b-18 calc.	b++ calc.	Seq.	y calc.	y++ calc.	y_3+ calc.	#
1	401.3132	**429.3081**	411.2976	215.1577	E				9
2	600.4448	**628.4397**	610.4291	314.7235	E	2953.2920	1477.1496	985.1022	8
3	728.5034	**756.4983**	738.4877	378.7528	Q	2754.1604	1377.5838	918.7250	7
4	875.5718	903.5667	885.5561	452.2870	F	2626.1018	1313.5546	876.0388	6
5	2774.2491	2802.2441	2784.2335	1401.6257	N	2479.0334	1240.0204	827.0160	5
6	2861.2812	2889.2761	2871.2655	1445.1417	S	580.3561	290.6817	194.1235	4
7	2962.3289	2990.3238	2972.3132	1495.6655	T	**493.3240**	247.1657	165.1129	3
8	3109.3973	3137.3922	3119.3816	1569.1997	F	392.2764	196.6418	131.4303	2
9					R	245.2080	123.1076	82.4075	1

HCD MS2 spectrum of precursor m/z = 846.1457, z = 4^+ at 17.4867 min. This spectrum was assigned to the EEQFNSTFR with N-glycan composition of H4N6.

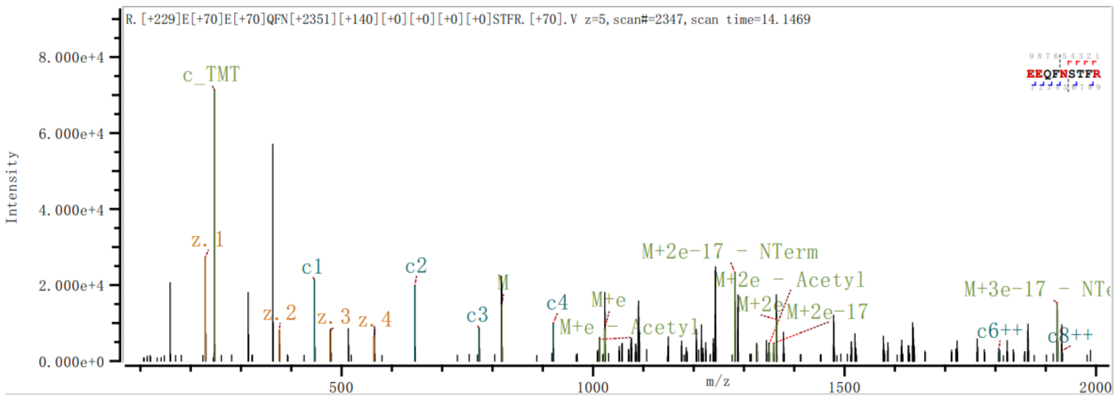

1745: EEQFNSTFR

#	a. calc.	c calc.	c++ calc.	Seq.	y calc.	z. calc.	z.++ calc.	#
1	402.3210	**446.3347**	223.6710	E				9
2	601.4526	**645.4663**	323.2368	E	3659.6661	3643.6474	1822.3273	8
3	729.5112	**773.5248**	387.2661	Q	3460.5345	3444.5158	1722.7615	7
4	876.5796	**920.5932**	460.8003	F	3332.4760	3316.4572	1658.7323	6
5	3481.6311	3525.6447	1763.3260	N	3185.4076	3169.3888	1585.1981	5
6	3568.6631	3612.6768	**1806.8420**	S	580.3561	**564.3373**		4
7	3669.7108	3713.7244	1857.3659	T	493.3240	**477.3053**		3
8	3816.7792	3860.7928	1930.9001	F	392.2764	**376.2576**		2
9				R	245.2080	**229.1892**		1

ETD MS2 spectrum of precursor m/z = 818.3982, z = 5^+ at 14.1469 min. This spectrum was assigned to the EEQFNSTFR with N-glycan composition of H5N4F1S2.

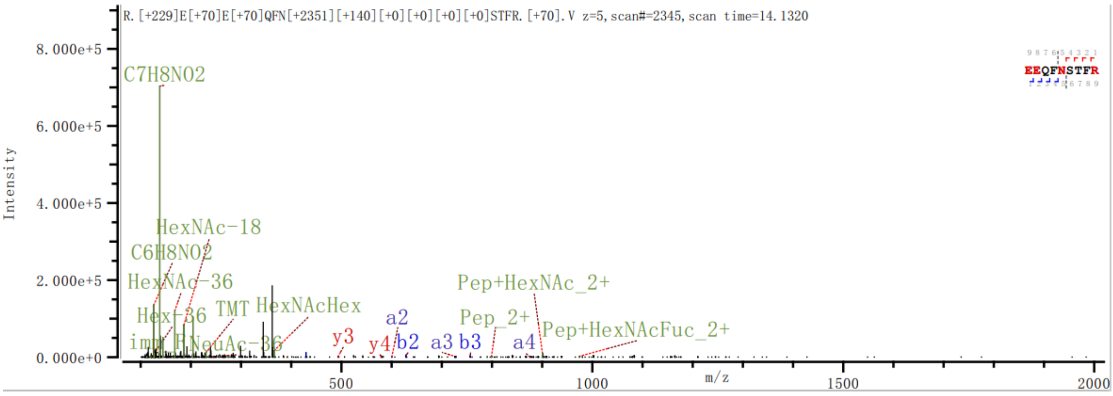

3994: EEQFNSTFR

#	a calc.	b calc.	b-18 calc.	b++ calc.	b_3+ calc.	Seq.	y calc.	y++ calc.	y_3+ calc.	#
1	401.3132	**429.3081**	411.2976	215.1577	143.7742	E				9
2	600.4448	**628.4397**	610.4291	314.7235	210.1514	E	3659.6661	1830.3367	1220.5602	8
3	728.5034	**756.4983**	738.4877	378.7528	252.8376	Q	3460.5345	1730.7709	1154.1830	7
4	875.5718	903.5667	885.5561	452.2870	301.8604	F	3332.4760	1666.7416	1111.4968	6
5	3480.6233	3508.6182	3490.6076	1754.8127	1170.2109	N	3185.4076	1593.2074	1062.4740	5
6	3567.6553	3595.6502	3577.6396	1798.3287	1199.2216	S	580.3561	290.6817	194.1235	4
7	3668.7030	3696.6979	3678.6873	1848.8526	1232.9041	T	**493.3240**	247.1657	165.1129	3
8	3815.7714	3843.7663	3825.7557	1922.3868	1281.9269	F	392.2764	196.6418	131.4303	2
9						R	245.2080	123.1076	82.4075	1

HCD MS2 spectrum of precursor m/z = 818.3982, z = 5^{+} at 14.1320 min. This spectrum was assigned to the EEQFNSTFR with N-glycan composition of H5N4F1S2.

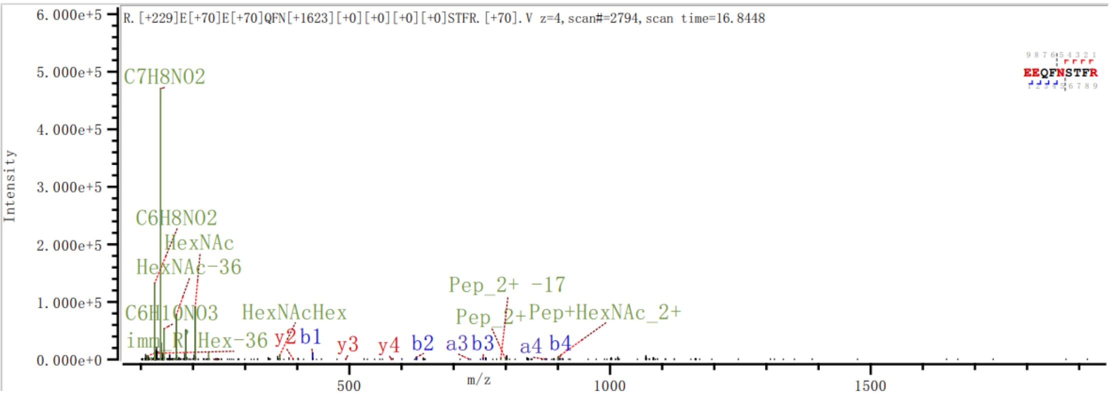

2656: EEQFNSTFR

#	a calc.	b calc.	b-18 calc.	b++ calc.	Seq.	y calc.	y++ calc.	y_3+ calc.	#
1	401.3132	**429.3081**	411.2976	215.1577	E				9
2	**600.4448**	**628.4397**	610.4291	314.7235	E	2791.2392	1396.1232	931.0846	8
3	728.5034	**756.4983**	738.4877	378.7528	Q	2592.1076	1296.5574	864.7074	7
4	875.5718	903.5667	885.5561	452.2870	F	2464.0490	1232.5281	822.0212	6
5	2612.1963	2640.1912	2622.1807	1320.5993	N	2316.9806	1158.9939	772.9984	5
6	2699.2284	2727.2233	2709.2127	1364.1153	S	**580.3561**	290.6817	194.1235	4
7	2800.2760	2828.2709	2810.2604	1414.6391	T	**493.3240**	247.1657	165.1129	3
8	2947.3444	2975.3394	2957.3288	1488.1733	F	392.2764	196.6418	131.4303	2
9					R	245.2080	123.1076	82.4075	1

HCD MS2 spectrum of precursor m/z = 805.6393, z = 4^+ at 16.8448 min. This spectrum was assigned to the EEQFNSTFR with N-glycan composition of H5N4.

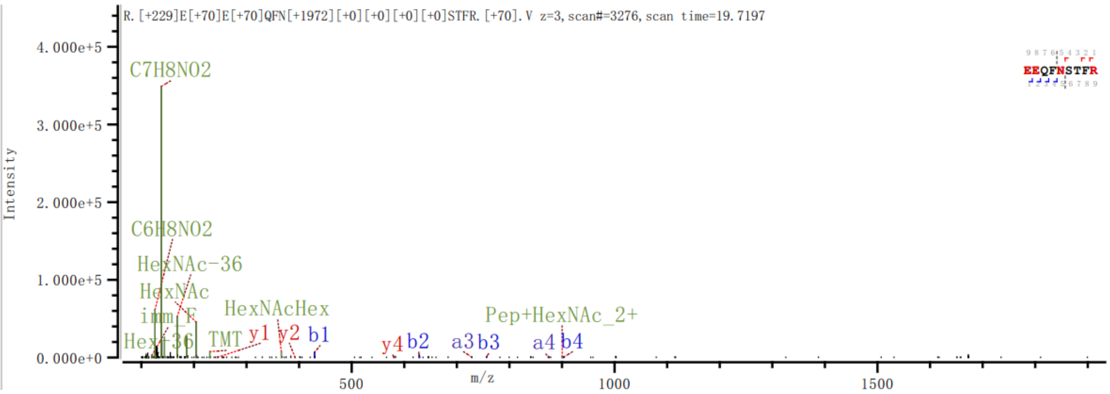

3246: EEQFNSTFR

#	a calc.	b calc.	b-18 calc.	b++ calc.	Seq.	y calc.	y++ calc.	#
1	401.3132	**429.3081**	411.2976	215.1577	E			9
2	600.4448	**628.4397**	610.4291	314.7235	E	3140.3765	1570.6919	8
3	**728.5034**	**756.4983**	738.4877	378.7528	Q	2941.2449	1471.1261	7
4	**875.5718**	**903.5667**	885.5561	452.2870	F	2813.1863	1407.0968	6
5	2961.3336	2989.3285	2971.3180	1495.1679	N	2666.1179	1333.5626	5
6	3048.3656	3076.3605	3058.3500	1538.6839	S	**580.3561**	290.6817	4
7	3149.4133	3177.4082	3159.3977	1589.2078	T	493.3240	247.1657	3
8	3296.4817	3324.4766	3306.4661	1662.7420	F	392.2764	196.6418	2
9					R	245.2080	123.1076	1

HCD MS2 spectrum of precursor m/z = 1190.2308, z = 3^+ at 19.7197 min. This spectrum was assigned to the EEQFNSTFR with N-glycan composition of H5N5F1.

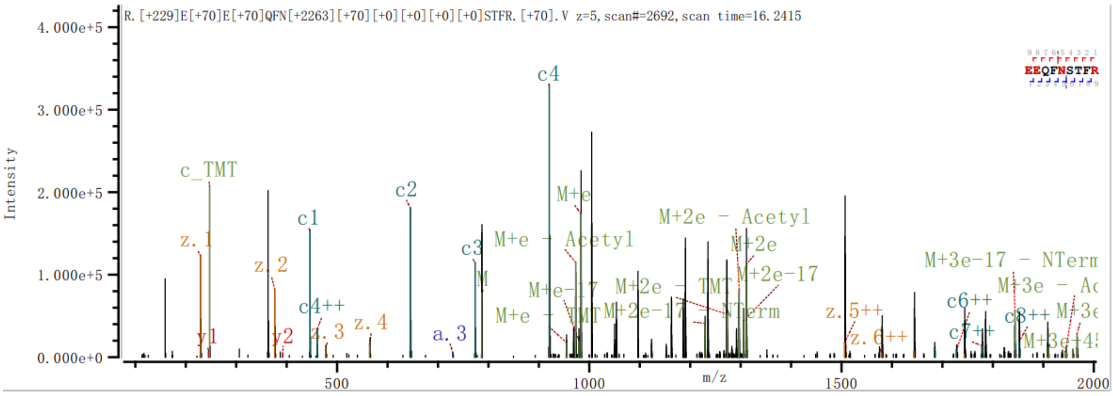

1749: EEQFNSTFR

#	a. calc.	c calc.	c++ calc.	Seq.	y calc.	z. calc.	z.++ calc.	#
1	402.3210	**446.3347**	223.6710	E				9
2	601.4526	**645.4663**	323.2368	E	3501.5610	3485.5423	1743.2748	8
3	729.5112	**773.5248**	387.2661	Q	3302.4294	3286.4107	1643.7090	7
4	876.5796	**920.5932**	460.8003	F	3174.3708	3158.3521	1579.6797	6
5	3323.5259	3367.5396	1684.2734	N	3027.3024	3011.2837	1506.1455	5
6	3410.5580	3454.5716	1727.7894	S	580.3561	**564.3373**		4
7	3511.6057	3555.6193	1778.3133	T	493.3240	477.3053		3
8	3658.6741	3702.6877	1851.8475	F	392.2764	**376.2576**		2
9				R	245.2080	**229.1892**		1

ETD MS2 spectrum of precursor m/z = 786.7786, z = 5^+ at 16.2415 min. This spectrum was assigned to the EEQFNSTFR with N-glycan composition of H5N5F1S1.

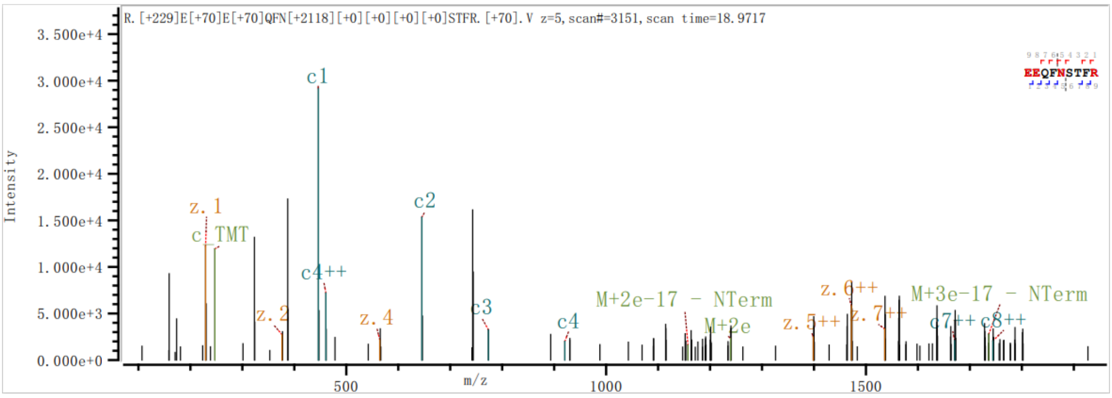

1410: EEQFNSTFR

#	a. calc.	c calc.	c++ calc.	Seq.	y calc.	z. calc.	z.++ calc.	#
1	402.3210	**446.3347**	223.6710	E				9
2	601.4526	**645.4663**	323.2368	E	3286.4344	3270.4157	1635.7115	8
3	729.5112	**773.5248**	387.2661	Q	3087.3028	3071.2841	1536.1457	7
4	876.5796	**920.5932**	460.8003	F	2959.2442	2943.2255	**1472.1164**	6
5	3108.3993	3152.4130	1576.7101	N	2812.1758	2796.1571	**1398.5822**	5
6	3195.4314	3239.4450	1620.2261	S	580.3561	**564.3373**		4
7	3296.4790	3340.4927	1670.7500	T	493.3240	477.3053		3
8	3443.5475	3487.5611	1744.2842	F	392.2764	376.2576		2
9				R	245.2080	**229.1892**		1

ETD MS2 spectrum of precursor m/z = 743.7533, z = 5^+ at 18.9717 min. This spectrum was assigned to the EEQFNSTFR with N-glycan composition of H5N5F2.

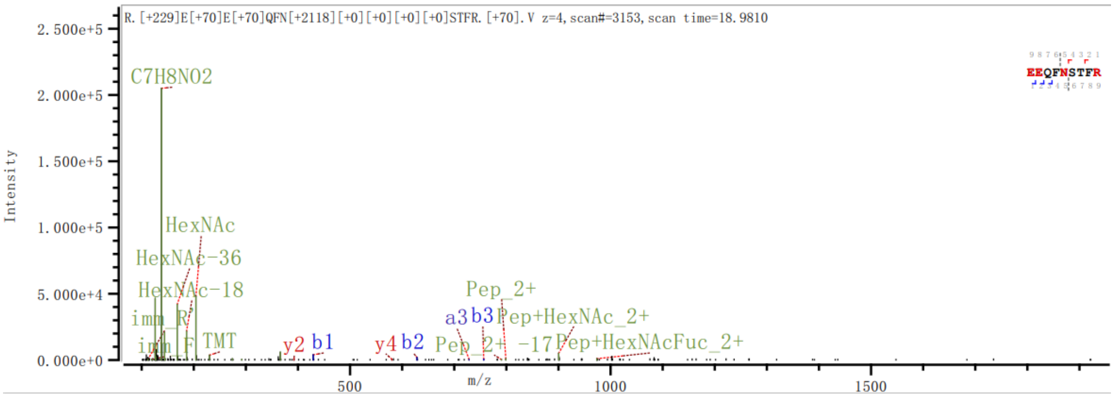

3660: EEQFNSTFR

#	a calc.	b calc.	b-18 calc.	b++ calc.	Seq.	y calc.	y++ calc.	y_3+ calc.	#
1	401.3132	**429.3081**	411.2976	215.1577	E				9
2	600.4448	**628.4397**	610.4291	314.7235	E	3286.4344	1643.7208	1096.1496	8
3	**728.5034**	**756.4983**	738.4877	378.7528	Q	3087.3028	1544.1550	1029.7724	7
4	875.5718	903.5667	885.5561	452.2870	F	2959.2442	1480.1257	987.0863	6
5	3107.3915	3135.3864	3117.3759	1568.1969	N	2812.1758	1406.5915	938.0635	5
6	3194.4235	3222.4185	3204.4079	1611.7129	S	**580.3561**	290.6817	194.1235	4
7	3295.4712	3323.4661	3305.4556	1662.2367	T	493.3240	247.1657	165.1129	3
8	3442.5396	3470.5345	3452.5240	1735.7709	F	392.2764	196.6418	131.4303	2
9					R	245.2080	123.1076	82.4075	1

HCD MS2 spectrum of precursor m/z = 929.4386, z = 4^{+} at 18.9810 min. This spectrum was assigned to the EEQFNSTFR with N-glycan composition of H5N5F2.

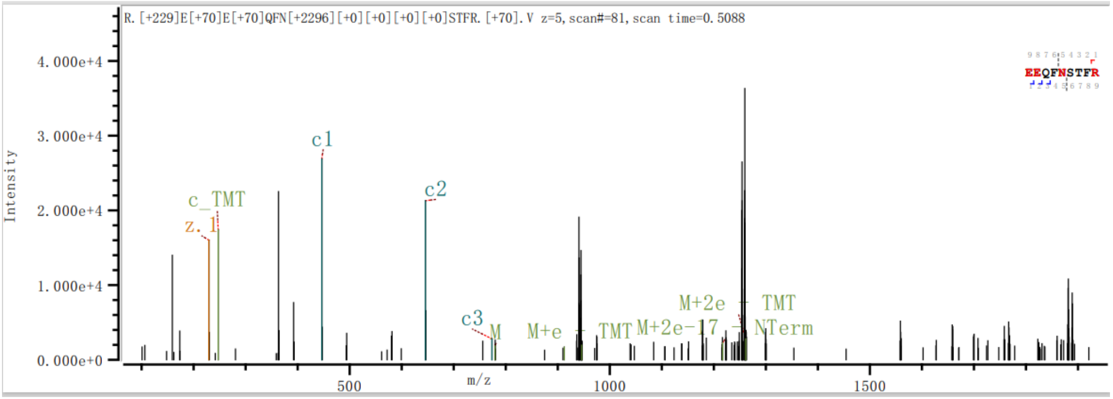

1692: EEQFNSTFR

#	a. calc.	c calc.	c++ calc.	Seq.	y calc.	z. calc.	z.++ calc.	#
1	402.3210	**446.3347**	223.6710	E				9
2	601.4526	**645.4663**	323.2368	E	3464.4821	3448.4634	1724.7353	8
3	729.5112	**773.5248**	387.2661	Q	3265.3505	3249.3318	1625.1695	7
4	876.5796	920.5932	460.8003	F	3137.2920	3121.2732	1561.1403	6
5	3286.4471	3330.4607	1665.7340	N	2990.2235	2974.2048	1487.6060	5
6	3373.4791	3417.4927	1709.2500	S	580.3561	564.3373		4
7	3474.5268	3518.5404	1759.7739	T	493.3240	477.3053		3
8	3621.5952	3665.6088	1833.3081	F	392.2764	376.2576		2
9				R	245.2080	**229.1892**		1

ETD MS2 spectrum of precursor m/z = 779.3648, z = 5^+ at 0.5088 min. This spectrum was assigned to the EEQFNSTFR with N-glycan composition of H5N7F1.

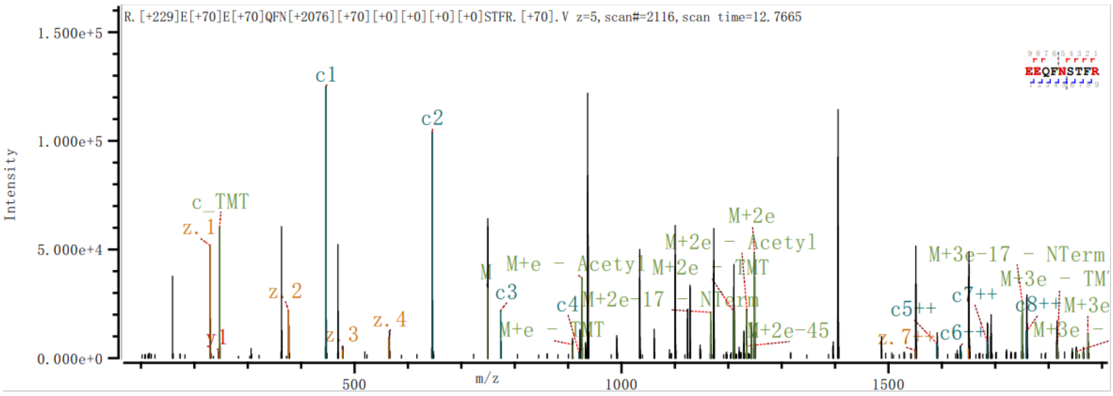

1356: EEQFNSTFR

#	a. calc.	c calc.	c++ calc.	Seq.	y calc.	z. calc.	z.++ calc.	#
1	402.3210	**446.3347**	223.6710	E				9
2	601.4526	**645.4663**	323.2368	E	3314.4765	3298.4578	1649.7325	8
3	729.5112	**773.5248**	387.2661	Q	3115.3449	3099.3262	1550.1667	7
4	876.5796	920.5932	460.8003	F	2987.2864	2971.2676	1486.1375	6
5	3136.4415	3180.4551	1590.7312	N	2840.2180	2824.1992	1412.6033	5
6	3223.4735	3267.4872	1634.2472	S	580.3561	**564.3373**		4
7	3324.5212	3368.5348	1684.7711	T	493.3240	477.3053		3
8	3471.5896	3515.6032	1758.3053	F	392.2764	**376.2576**		2
9				R	**245.2080**	**229.1892**		1

ETD MS2 spectrum of precursor m/z = 749.3604, z = 5^{+} at 12.7665 min. This spectrum was assigned to the EEQFNSTFR with N-glycan composition of H6N4S1.

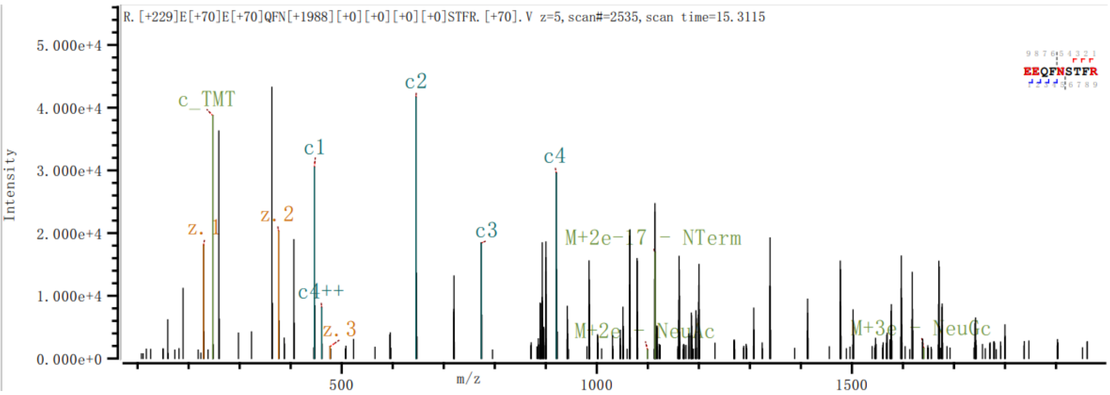

915: EEQFNSTFR

#	a. calc.	c calc.	c++ calc.	Seq.	y calc.	z. calc.	z.++ calc.	#
1	402.3210	**446.3347**	223.6710	E				9
2	601.4526	**645.4663**	323.2368	E	3156.3714	3140.3527	1570.6800	8
3	729.5112	**773.5248**	387.2661	Q	2957.2398	2941.2211	1471.1142	7
4	876.5796	**920.5932**	460.8003	F	2829.1812	2813.1625	1407.0849	6
5	2978.3363	3022.3500	1511.6786	N	2682.1128	2666.0941	1333.5507	5
6	3065.3684	3109.3820	1555.1946	S	580.3561	564.3373		4
7	3166.4160	3210.4297	1605.7185	T	493.3240	477.3053		3
8	3313.4845	3357.4981	1679.2527	F	392.2764	**376.2576**		2
9				R	245.2080	**229.1892**		1

ETD MS2 spectrum of precursor m/z = 717.7390, z = 5^+ at 15.3115 min. This spectrum was assigned to the EEQFNSTFR with N-glycan composition of H6N5.

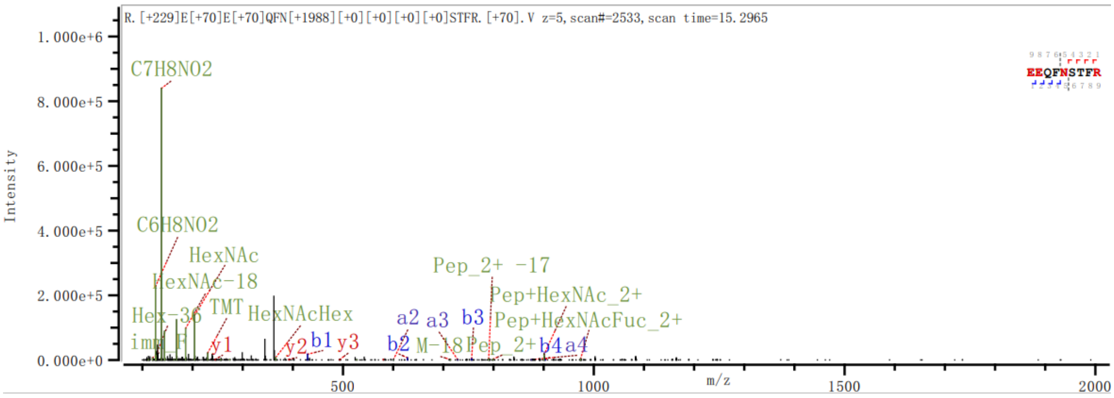

3164: EEQFNSTFR

#	a calc.	b calc.	b-18 calc.	b++ calc.	b_3+ calc.	Seq.	y calc.	y++ calc.	y_3+ calc.	#
1	401.3132	**429.3081**	411.2976	215.1577	143.7742	E				9
2	600.4448	**628.4397**	610.4291	314.7235	210.1514	E	3156.3714	1578.6893	1052.7953	8
3	728.5034	**756.4983**	738.4877	378.7528	252.8376	Q	2957.2398	1479.1235	986.4181	7
4	875.5718	903.5667	885.5561	452.2870	301.8604	F	2829.1812	1415.0942	943.7319	6
5	2977.3285	3005.3234	2987.3129	1503.1654	1002.4460	N	2682.1128	1341.5600	894.7091	5
6	3064.3605	3092.3555	3074.3449	1546.6814	1031.4567	S	580.3561	290.6817	194.1235	4
7	3165.4082	3193.4031	3175.3926	1597.2052	1065.1392	T	**493.3240**	247.1657	165.1129	3
8	3312.4766	3340.4715	3322.4610	1670.7394	1114.1620	F	392.2764	196.6418	131.4303	2
9						R	245.2080	123.1076	82.4075	1

HCD MS2 spectrum of precursor m/z = 717.7390, z = 5^{+} at 15.2965 min. This spectrum was assigned to the EEQFNSTFR with N-glycan composition of H6N5.

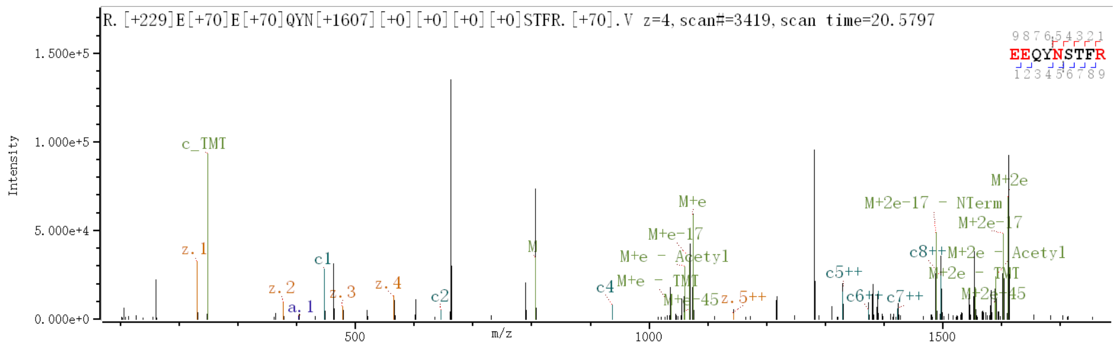

PID=434: EEQYNSTFR

#	a. calc.	c calc.	c++ calc.	c-HexN calc.	c-HexNAc calc.	Seq.	y calc.	z. calc.	z.++ calc.	#
1	**402.3210**	**446.3347**	223.6710			E				9
2	601.4526	**645.4663**	323.2368			E	2791.2392		1388.1139	8
3	729.5112	773.5248	387.2661			Q	2592.1076		1288.5481	7
4	892.5745	**936.5882**	468.7977			Y	2464.0490		1224.5188	6
5	2613.2041	2657.2178	**1329.1125**			N	2300.9857	2284.9670	**1142.9871**	5
6	2700.2362	2744.2498	**1372.6285**			S	580.3561	**564.3373**	282.6723	4
7	2801.2839	2845.2975	**1423.1524**			T	493.3240	**477.3053**		3
8	2948.3523	2992.3659	**1496.6866**			F	392.2764	**376.2576**		2
9						R	245.2080	**229.1892**		1

ETD MS2 spectrum of precursor m/z = 805.6387, z = 4^{+} at 20.5797 min. This spectrum was assigned to the EEQYNSTFR with N-glycan composition of H4N4F1.

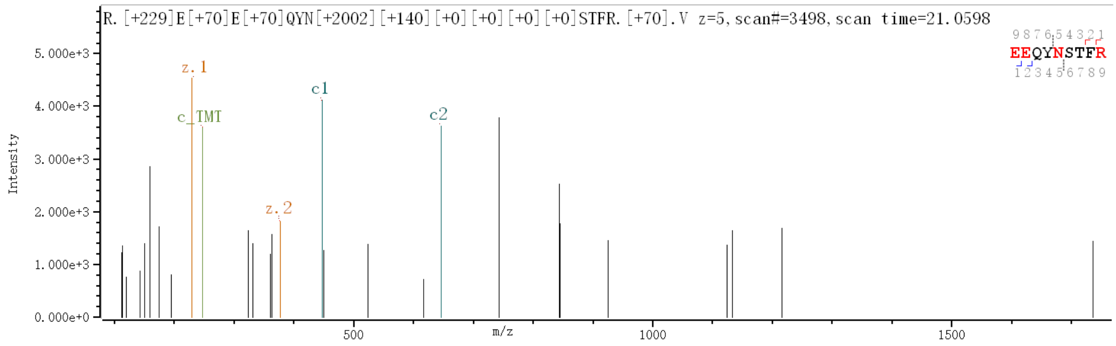

PID=1463: EEQYNSTFR

#	a. calc.	c calc.	c++ calc.	c-HexN calc.	c-HexNAc calc.	Seq.	y calc.	z. calc.	z.++ calc.	#
1	402.3210	**446.3347**	223.6710			E				9
2	601.4526	**645.4663**	323.2368			E	3326.5238		1655.7562	8
3	729.5112	773.5248	387.2661			Q	3127.3922		1556.1904	7
4	892.5745	936.5882	468.7977			Y	2999.3336		1492.1611	6
5	3148.4887	3192.5024	1596.7548			N	2836.2703	2820.2515	1410.6294	5
6	3235.5208	3279.5344	1640.2708			S	580.3561	564.3373	282.6723	4
7	3336.5684	3380.5821	1690.7947			T	493.3240	477.3053		3
8	3483.6368	3527.6505	1764.3289			F	392.2764	**376.2576**		2
9						R	245.2080	**229.1892**		1

ETD MS2 spectrum of precursor m/z = 751.7714, z = 5^{+} at 21.0598 min. This spectrum was assigned to the EEQYNSTFR with N-glycan composition of H5N3S2.

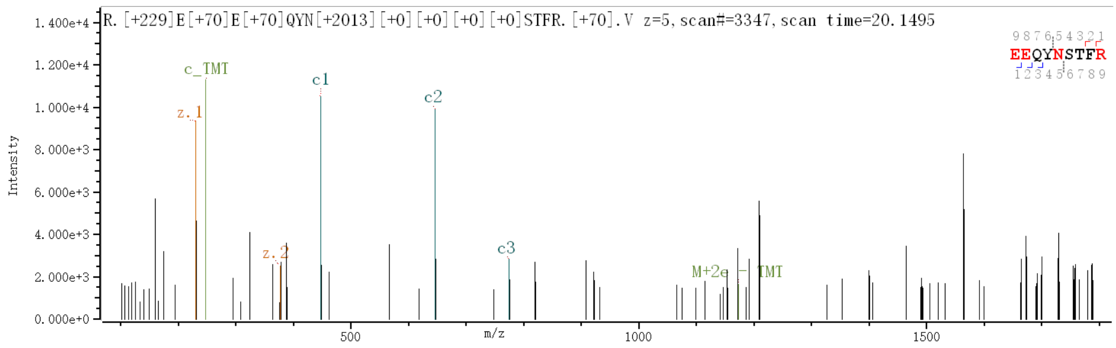

PID=1441: EEQYNSTFR

#	a. calc.	c calc.	c++ calc.	c-HexN calc.	c-HexNAc calc.	Seq.	y calc.	z. calc.	z.++ calc.	#
1	402.3210	**446.3347**	223.6710			E				9
2	601.4526	**645.4663**	323.2368			E	3197.3979		1591.1932	8
3	729.5112	**773.5248**	387.2661			Q	2998.2664		1491.6275	7
4	892.5745	936.5882	468.7977			Y	2870.2078		1427.5982	6
5	3019.3629	3063.3765	1532.1919			N	2707.1444	2691.1257	1346.0665	5
6	3106.3949	3150.4086	1575.7079			S	580.3561	564.3373	282.6723	4
7	3207.4426	3251.4562	1626.2318			T	493.3240	477.3053		3
8	3354.5110	3398.5247	1699.7660			F	392.2764	**376.2576**		2
9						R	245.2080	**229.1892**		1

ETD MS2 spectrum of precursor m/z = 725.9478, z = 5^{+} at 20.1495 min. This spectrum was assigned to the EEQYNSTFR with N-glycan composition of H4N6F1.

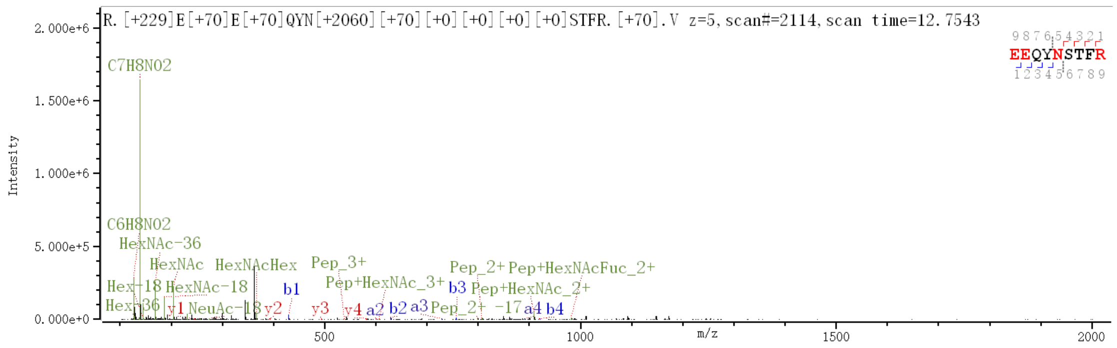

PID=3605: EEQYNSTFR

#	a calc.	b calc.	b-18 calc.	b++ calc.	b++ -HexNAc calc.	b++ -HexN calc.	b-HexN calc.	b_3+ calc.	b_4+ calc.	Seq.	y calc.	y++ calc.	y_3+ calc.	y_4+ calc.	#
1	401.3132	**429.3081**	411.2976	215.1577				143.7742	108.0825	E					9
2	**600.4448**	**628.4397**	610.4291	314.7235				210.1514	157.8654	E	3314.4765	1657.7419	1105.4970	829.3746	8
3	**728.5034**	**756.4983**	738.4877	378.7528				252.8376	189.8800	Q	3115.3449	1558.1761	1039.1198	779.5917	7
4	**891.5667**	**919.5616**	901.5511	460.2844				307.1921	230.6459	Y	2987.2864	1494.1468	996.4336	747.5770	6
5	3135.4337	3163.4286	3145.4180	1582.2179				1055.1477	791.6126	N	2824.2230	1412.6152	942.0792	706.8112	5
6	3222.4657	3250.4606	3232.4500	1625.7339				1084.1584	813.3706	S	**580.3561**	290.6817	194.1235	145.8445	4
7	3323.5134	3351.5083	3333.4977	1676.2578				1117.8409	838.6325	T	**493.3240**	247.1657	165.1129	124.0865	3
8	3470.5818	3498.5767	3480.5661	1749.7920				1166.8637	875.3996	F	**392.2764**	196.6418	131.4303	98.8245	2
9										R	**245.2080**	123.1076	82.4075	62.0574	1

HCD MS2 spectrum of precursor m/z = 749.3604, z = 5^+ at 21.7543 min. This spectrum was assigned to the EEQYNSTFR with N-glycan composition of H5N4F1S1.

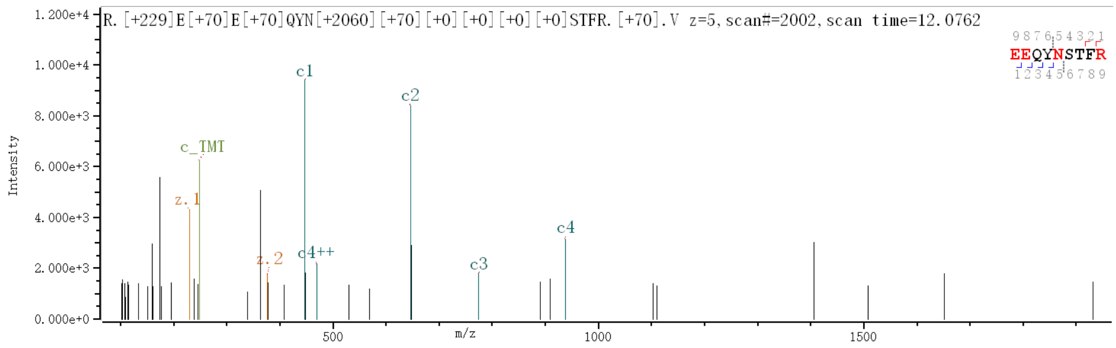

PID=1334: EEQYNSTFR

#	a. calc.	c calc.	c++ calc.	c-HexN calc.	c-HexNAc calc.	Seq.	y calc.	z. calc.	z.++ calc.	#
1	402.3210	**446.3347**	223.6710			E				9
2	601.4526	**645.4663**	323.2368			E	3314.4765		1649.7325	8
3	729.5112	**773.5248**	387.2661			Q	3115.3449		1550.1667	7
4	892.5745	**936.5882**	**468.7977**			Y	2987.2864		1486.1375	6
5	3136.4415	3180.4551	1590.7312			N	2824.2230	2808.2043	1404.6058	5
6	3223.4735	3267.4872	1634.2472			S	580.3561	564.3373	282.6723	4
7	3324.5212	3368.5348	1684.7711			T	493.3240	477.3053		3
8	3471.5896	3515.6032	1758.3053			F	392.2764	**376.2576**		2
9						R	245.2080	**229.1892**		1

ETD MS2 spectrum of precursor m/z = 749.3617, z = 5^+ at 12.0762 min. This spectrum was assigned to the EEQYNSTFR with N-glycan composition of H5N4F1S1.

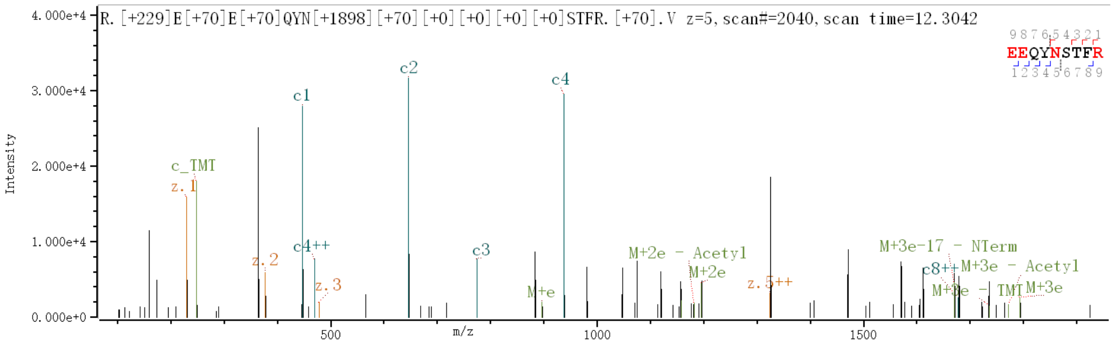

PID=854: EEQYNSTFR

#	a. calc.	c calc.	c++ calc.	c-HexN calc.	c-HexNAc calc.	Seq.	y calc.	z. calc.	z.++ calc.	#
1	402.3210	**446.3347**	223.6710			E				9
2	601.4526	**645.4663**	323.2368			E	3152.4237		1568.7061	8
3	729.5112	**773.5248**	387.2661			Q	2953.2921		1469.1403	7
4	892.5745	**936.5882**	**468.7977**			Y	2825.2335		1405.1110	6
5	2974.3887	3018.4023	1509.7048			N	2662.1702	2646.1515	**1323.5794**	5
6	3061.4207	3105.4343	1553.2208			S	580.3561	564.3373	282.6723	4
7	3162.4684	3206.4820	1603.7446			T	493.3240	**477.3053**		3
8	3309.5368	3353.5504	**1677.2789**			F	392.2764	**376.2576**		2
9						R	245.2080	**229.1892**		1

ETD MS2 spectrum of precursor m/z = 716.9484, z = 5^+ at 12.3042 min. This spectrum was assigned to the EEQYNSTFR with N-glycan composition of H4N4F1S1.

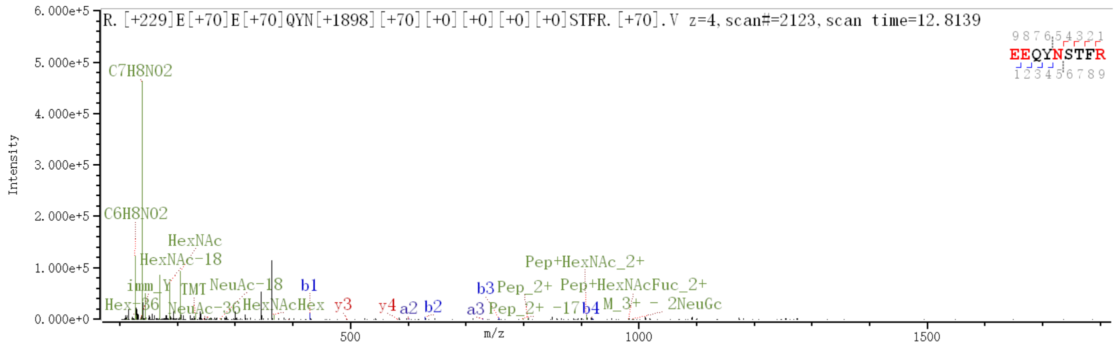

PID=3124: EEQYNSTFR

#	a calc.	b calc.	b-18 calc.	b++ calc.	b++ -HexNAc calc.	b++ -HexN calc.	b-HexN calc.	b_3+ calc.	Seq.	y calc.	y++ calc.	y_3+ calc.	#
1	401.3132	**429.3081**	411.2976	215.1577				143.7742	E				9
2	**600.4448**	**628.4397**	610.4291	314.7235				210.1514	E	3152.4237	1576.7155	1051.4794	8
3	**728.5034**	**756.4983**	738.4877	378.7528				252.8376	Q	2953.2921	1477.1497	985.1022	7
4	891.5667	**919.5616**	901.5511	460.2844				307.1921	Y	2825.2335	1413.1204	942.4160	6
5	2973.3808	3001.3758	2983.3652	1501.1915				1001.1301	N	2662.1702	1331.5887	888.0616	5
6	3060.4129	3088.4078	3070.3972	1544.7075				1030.1408	S	**580.3561**	290.6817	194.1235	4
7	3161.4606	3189.4555	3171.4449	1595.2314				1063.8233	T	**493.3240**	247.1657	165.1129	3
8	3308.5290	3336.5239	3318.5133	1668.7656				1112.8461	F	**392.2764**	196.6418	131.4303	2
9									R	**245.2080**	123.1076	82.4075	1

HCD MS2 spectrum of precursor m/z = 895.9372, z = 4^+ at 12.8139 min. This spectrum was assigned to the EEQYNSTFR with N-glycan composition of H4N4F1S1.

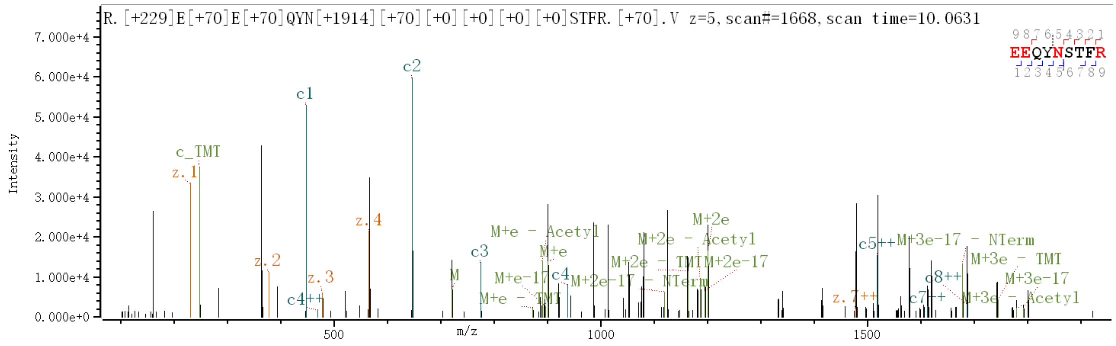

PID=772: EEQYNSTFR

#	a. calc.	c calc.	c++ calc.	c-HexN calc.	c-HexNAc calc.	Seq.	y calc.	z. calc.	z.++ calc.	#
1	402.3210	**446.3347**	223.6710			E				9
2	601.4526	**645.4663**	323.2368			E	3168.4186		1576.7036	8
3	729.5112	**773.5248**	387.2661			Q	2969.2870		**1477.1378**	7
4	892.5745	**936.5882**	**468.7977**			Y	2841.2285		1413.1085	6
5	2990.3836	3034.3972	**1517.7022**			N	2678.1651	2662.1464	1331.5768	5
6	3077.4156	3121.4292	1561.2183			S	580.3561	**564.3373**	282.6723	4
7	3178.4633	3222.4769	**1611.7421**			T	493.3240	**477.3053**		3
8	3325.5317	3369.5453	**1685.2763**			F	392.2764	**376.2576**		2
9						R	245.2080	**229.1892**		1

ETD MS2 spectrum of precursor m/z = 720.1491, z = 5^+ at 10.0631 min. This spectrum was assigned to the EEQYNSTFR with N-glycan composition of H5N4S1.

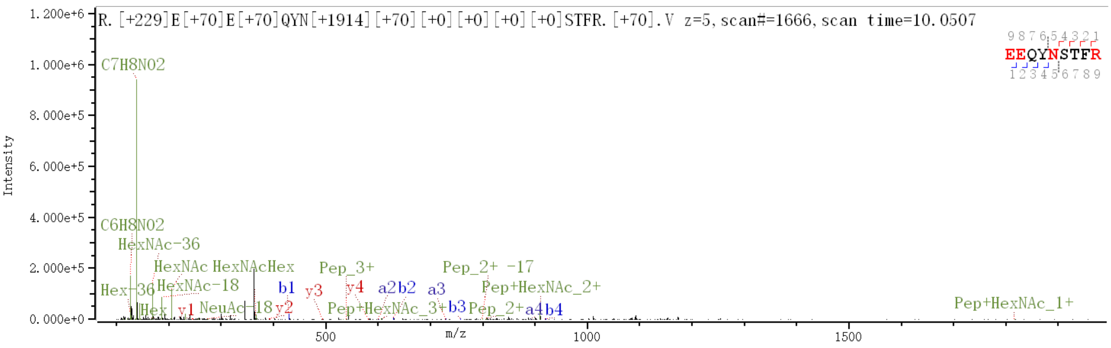

PID=3021: EEQYNSTFR

#	a calc.	b calc.	b-18 calc.	b++ calc.	b++ -HexNAc calc.	b++ -HexN calc.	b-HexN calc.	b_3+ calc.	b_4+ calc.	Seq.	y calc.	y++ calc.	y_3+ calc.	y_4+ calc.	#
1	401.3132	**429.3081**	411.2976	215.1577				143.7742	108.0825	E					9
2	**600.4448**	**628.4397**	610.4291	314.7235				210.1514	157.8654	E	3168.4186	1584.7129	1056.8111	792.8601	8
3	**728.5034**	**756.4983**	738.4877	378.7528				252.8376	189.8800	Q	2969.2870	1485.1472	990.4339	743.0772	7
4	**891.5667**	**919.5616**	901.5511	460.2844				307.1921	230.6459	Y	2841.2285	1421.1179	947.7477	711.0626	6
5	2989.3758	3017.3707	2999.3601	1509.1890				1006.4617	755.0981	N	2678.1651	1339.5862	893.3932	670.2967	5
6	3076.4078	3104.4027	3086.3921	1552.7050				1035.4724	776.8561	S	**580.3561**	290.6817	194.1235	145.8445	4
7	3177.4555	3205.4504	3187.4398	1603.2288				1069.1550	802.1181	T	**493.3240**	247.1657	165.1129	124.0865	3
8	3324.5239	3352.5188	3334.5082	1676.7630				1118.1778	838.8852	F	**392.2764**	196.6418	131.4303	98.8245	2
9										R	**245.2080**	123.1076	82.4075	62.0574	1

HCD MS2 spectrum of precursor m/z = 720.1491, z = 5^{+} at 10.0507 min. This spectrum was assigned to the EEQYNSTFR with N-glycan composition of H5N4S1.

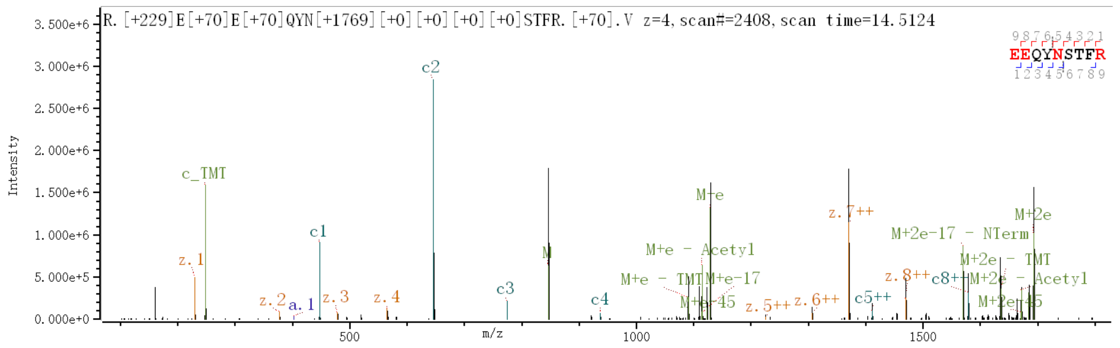

PID=893: EEQYNSTFR

#	a. calc.	c calc.	c++ calc.	c-HexN calc.	c-HexNAc calc.	Seq.	y calc.	z. calc.	z.++ calc.	#
1	**402.3210**	**446.3347**	223.6710			E				9
2	601.4526	**645.4663**	323.2368			E	2953.2920		**1469.1403**	8
3	729.5112	**773.5248**	387.2661			Q	2754.1604		**1369.5745**	7
4	892.5745	**936.5882**	468.7977			Y	2626.1019		**1305.5452**	6
5	2775.2570	2819.2706	**1410.1389**			N	2463.0385	2447.0198	**1224.0135**	5
6	2862.2890	2906.3026	1453.6550			S	580.3561	**564.3373**	282.6723	4
7	2963.3367	3007.3503	1504.1788			T	493.3240	**477.3053**		3
8	3110.4051	3154.4187	**1577.7130**			F	392.2764	**376.2576**		2
9						R	245.2080	**229.1892**		1

ETD MS2 spectrum of precursor m/z = 846.1520, z = 4^+ at 14.5124 min. This spectrum was assigned to the EEQYNSTFR with N-glycan composition of H5N4F1.

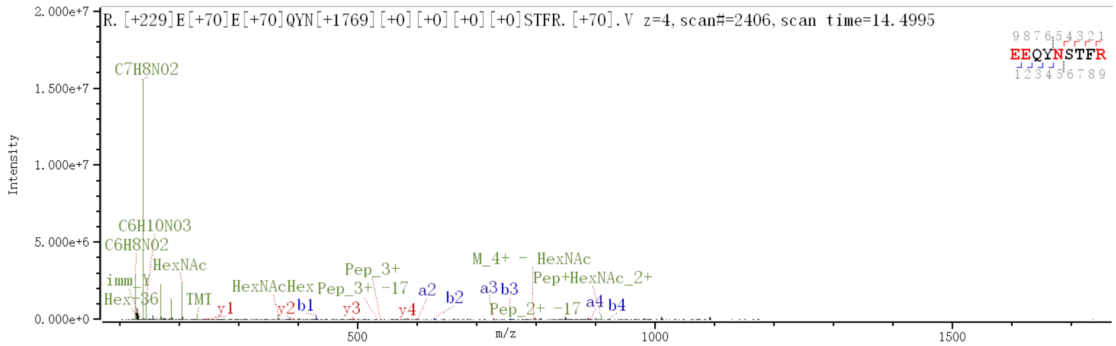

PID=3142: EEQYNSTFR

#	a calc.	b calc.	b-18 calc.	b++ calc.	b++ -HexNAc calc.	b++ -HexN calc.	b-HexN calc.	b_3+ calc.	Seq.	y calc.	y++ calc.	y_3+ calc.	#
1	401.3132	**429.3081**	411.2976	215.1577				143.7742	E				9
2	**600.4448**	**628.4397**	**610.4291**	314.7235				210.1514	E	2953.2920	1477.1496	985.1022	8
3	**728.5034**	**756.4983**	738.4877	378.7528				252.8376	Q	2754.1604	1377.5839	918.7250	7
4	**891.5667**	**919.5616**	901.5511	460.2844				307.1921	Y	2626.1019	1313.5546	876.0388	6
5	2774.2492	2802.2441	2784.2335	1401.6257				934.7529	N	2463.0385	1232.0229	821.6844	5
6	2861.2812	2889.2761	2871.2655	1445.1417				963.7635	S	**580.3561**	290.6817	194.1235	4
7	2962.3289	2990.3238	2972.3132	1495.6655				997.4461	T	**493.3240**	247.1657	165.1129	3
8	3109.3973	3137.3922	3119.3816	1569.1997				1046.4689	F	**392.2764**	196.6418	131.4303	2
9									R	**245.2080**	123.1076	82.4075	1

HCD MS2 spectrum of precursor m/z = 846.1520, z = 4^{+} at 14.4995 min. This spectrum was assigned to the EEQYNSTFR with N-glycan composition of H5N4F1.

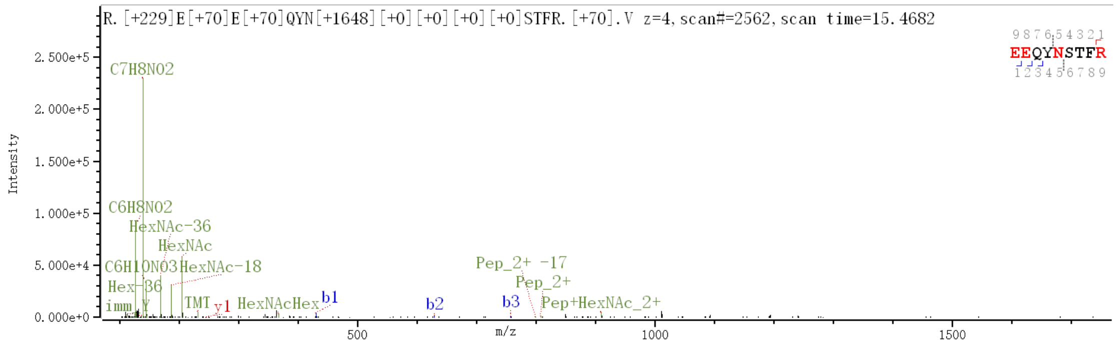

PID=2650: EEQYNSTFR

#	a calc.	b calc.	b-18 calc.	b++ calc.	b++ -HexNAc calc.	b++ -HexN calc.	b-HexN calc.	b_3+ calc.	Seq.	y calc.	y++ calc.	y_3+ calc.	#
1	401.3132	**429.3081**	411.2976	215.1577				143.7742	E				9
2	600.4448	**628.4397**	610.4291	314.7235				210.1514	E	2832.2657	1416.6365	944.7601	8
3	728.5034	**756.4983**	738.4877	378.7528				252.8376	Q	2633.1342	1317.0707	878.3829	7
4	891.5667	919.5616	901.5511	460.2844				307.1921	Y	2505.0756	1253.0414	835.6967	6
5	2653.2229	2681.2178	2663.2072	1341.1125				894.4108	N	2342.0122	1171.5098	781.3423	5
6	2740.2549	2768.2498	2750.2393	1384.6285				923.4215	S	580.3561	290.6817	194.1235	4
7	2841.3026	2869.2975	2851.2869	1435.1524				957.1040	T	493.3240	247.1657	165.1129	3
8	2988.3710	3016.3659	2998.3553	1508.6866				1006.1268	F	392.2764	196.6418	131.4303	2
9									R	**245.2080**	123.1076	82.4075	1

HCD MS2 spectrum of precursor m/z = 815.8931, z = 4^{+} at 15.4682 min. This spectrum was assigned to the EEQYNSTFR with N-glycan composition of H3N5F1.

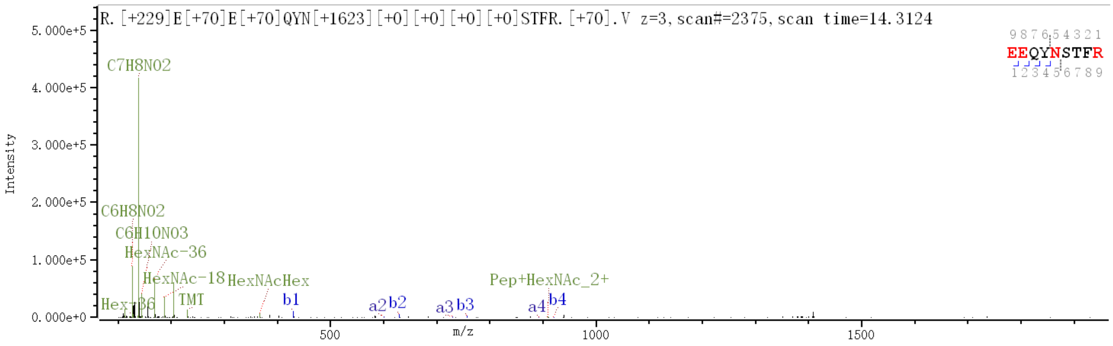

PID=2630: EEQYNSTFR

#	a calc.	b calc.	b-18 calc.	b++ calc.	b++ -HexNAc calc.	b++ -HexN calc.	b-HexN calc.	Seq.	y calc.	y++ calc.	#
1	401.3132	**429.3081**	411.2976	215.1577				E			9
2	**600.4448**	**628.4397**	610.4291	314.7235				E	2807.2341	1404.1207	8
3	**728.5034**	**756.4983**	738.4877	378.7528				Q	2608.1025	1304.5549	7
4	**891.5667**	**919.5616**	901.5511	460.2844				Y	2480.0439	1240.5256	6
5	2628.1912	2656.1862	2638.1756	1328.5967				N	2316.9806	1158.9939	5
6	2715.2233	2743.2182	2725.2076	1372.1127				S	580.3561	290.6817	4
7	2816.2710	2844.2659	2826.2553	1422.6366				T	493.3240	247.1657	3
8	2963.3394	2991.3343	2973.3237	1496.1708				F	392.2764	196.6418	2
9								R	245.2080	123.1076	1

HCD MS2 spectrum of precursor m/z = 1079.1841, z = 3^{+} at 14.3124 min. This spectrum was assigned to the EEQYNSTFR with N-glycan composition of H5N4.

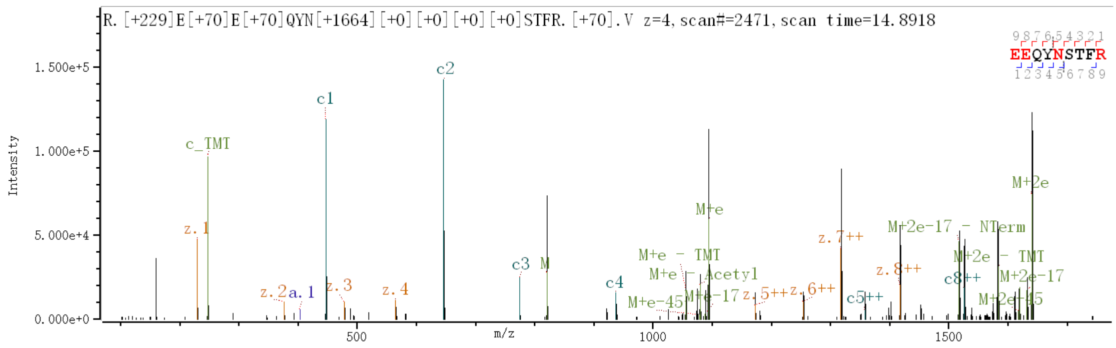

PID=393: EEQYNSTFR

#	a. calc.	c calc.	c++ calc.	c-HexN calc.	c-HexNAc calc.	Seq.	y calc.	z. calc.	z.++ calc.	#
1	**402.3210**	**446.3347**	223.6710			E				9
2	601.4526	**645.4663**	323.2368			E	2848.2607		**1416.6246**	8
3	729.5112	**773.5248**	387.2661			Q	2649.1291		**1317.0588**	7
4	892.5745	**936.5882**	468.7977			Y	2521.0705		**1253.0295**	6
5	2670.2256	2714.2393	**1357.6233**			N	2358.0072	2341.9884	**1171.4979**	5
6	2757.2576	2801.2713	1401.1393			S	580.3561	**564.3373**	282.6723	4
7	2858.3053	2902.3190	1451.6631			T	493.3240	**477.3053**		3
8	3005.3737	3049.3874	**1525.1973**			F	392.2764	**376.2576**		2
9						R	245.2080	**229.1892**		1

ETD MS2 spectrum of precursor m/z = 819.8942, z = 4^+ at 14.8918 min. This spectrum was assigned to the EEQYNSTFR with N-glycan composition of H4N5.

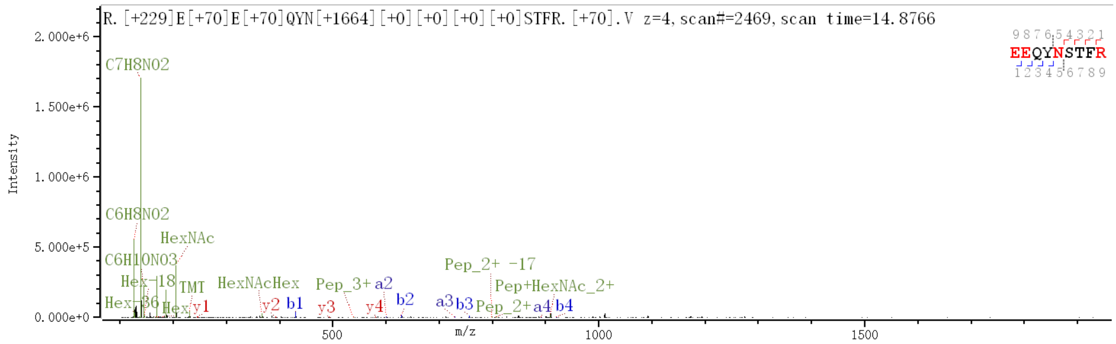

PID=2642: EEQYNSTFR

#	a calc.	b calc.	b-18 calc.	b++ calc.	b++ -HexNAc calc.	b++ -HexN calc.	b-HexN calc.	b_3+ calc.	Seq.	y calc.	y++ calc.	y_3+ calc.	#
1	401.3132	**429.3081**	411.2976	215.1577				143.7742	E				9
2	**600.4448**	**628.4397**	610.4291	314.7235				210.1514	E	2848.2607	1424.6340	950.0917	8
3	**728.5034**	**756.4983**	738.4877	378.7528				252.8376	Q	2649.1291	1325.0682	883.7145	7
4	**891.5667**	**919.5616**	901.5511	460.2844				307.1921	Y	2521.0705	1261.0389	841.0283	6
5	2669.2178	2697.2127	2679.2021	1349.1100				899.7424	N	2358.0072	1179.5072	786.6739	5
6	2756.2498	2784.2447	2766.2342	1392.6260				928.7531	S	**580.3561**	290.6817	194.1235	4
7	2857.2975	2885.2924	2867.2819	1443.1498				962.4357	T	**493.3240**	247.1657	165.1129	3
8	3004.3659	3032.3608	3014.3503	1516.6841				1011.4585	F	**392.2764**	196.6418	131.4303	2
9									R	**245.2080**	123.1076	82.4075	1

HCD MS2 spectrum of precursor m/z = 819.8942, z = 4^+ at 14.8766 min. This spectrum was assigned to the EEQYNSTFR with N-glycan composition of H4N5.

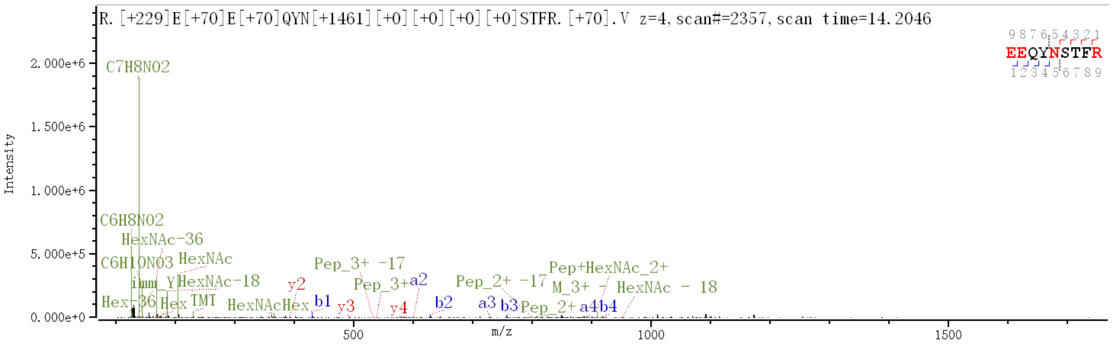

PID=2624: EEQYNSTFR

#	a calc.	b calc.	b-18 calc.	b++ calc.	b++ -HexNAc calc.	b++ -HexN calc.	b-HexN calc.	b_3+ calc.	Seq.	y calc.	y++ calc.	y_3+ calc.	#
1	401.3132	**429.3081**	411.2976	215.1577				143.7742	E				9
2	**600.4448**	**628.4397**	**610.4291**	314.7235				210.1514	E	2645.1813	1323.0943	882.3986	8
3	**728.5034**	**756.4983**	738.4877	378.7528				252.8376	Q	2446.0497	1223.5285	816.0214	7
4	**891.5667**	**919.5616**	901.5511	460.2844				307.1921	Y	2317.9911	1159.4992	773.3352	6
5	2466.1384	2494.1333	2476.1228	1247.5703				832.0493	N	2154.9278	1077.9675	718.9808	5
6	2553.1704	2581.1654	2563.1548	1291.0863				861.0600	S	**580.3561**	290.6817	194.1235	4
7	2654.2181	2682.2130	2664.2025	1341.6102				894.7425	T	**493.3240**	247.1657	165.1129	3
8	2801.2865	2829.2814	2811.2709	1415.1444				943.7653	F	**392.2764**	196.6418	131.4303	2
9									R	**245.2080**	123.1076	82.4075	1

HCD MS2 spectrum of precursor m/z = 769.1262, z = 4^+ at 14.2046 min. This spectrum was assigned to the EEQYNSTFR with N-glycan composition of H4N4.

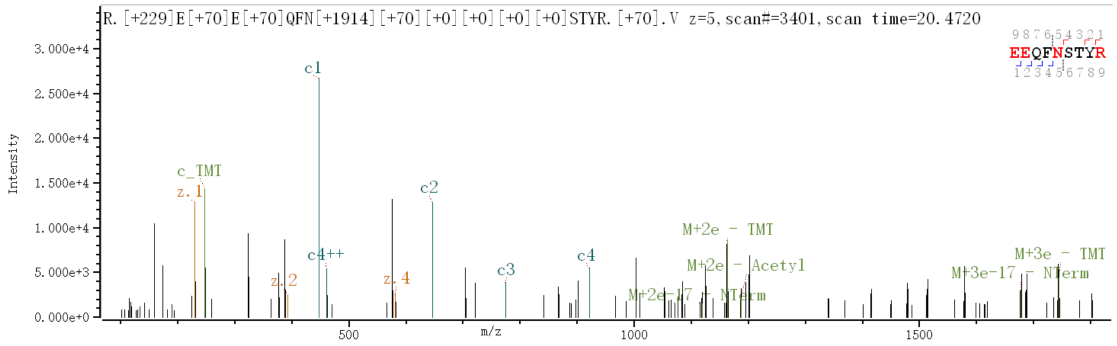

PID=1015: EEQFNSTYR

#	a. calc.	c calc.	c++ calc.	c-HexN calc.	c-HexNAc calc.	Seq.	y calc.	z. calc.	z.++ calc.	#
1	402.3210	**446.3347**	223.6710			E				9
2	601.4526	**645.4663**	323.2368			E	3168.4186		1576.7036	8
3	729.5112	**773.5248**	387.2661			Q	2969.2870		1477.1378	7
4	876.5796	**920.5932**	**460.8003**			F	2841.2285		1413.1085	6
5	2974.3887	3018.4023	1509.7048			N	2694.1600	2678.1413	1339.5743	5
6	3061.4207	3105.4343	1553.2208			S	596.3510	**580.3323**	290.6698	4
7	3162.4684	3206.4820	1603.7446			T	509.3190	493.3002		3
8	3325.5317	3369.5453	1685.2763			Y	408.2713	**392.2526**		2
9						R	245.2080	**229.1892**		1

ETD MS2 spectrum of precursor m/z = 720.1442, z = 5^+ at 20.4720 min. This spectrum was assigned to the EEQFNSTYR with N-glycan composition of H5N4S1.

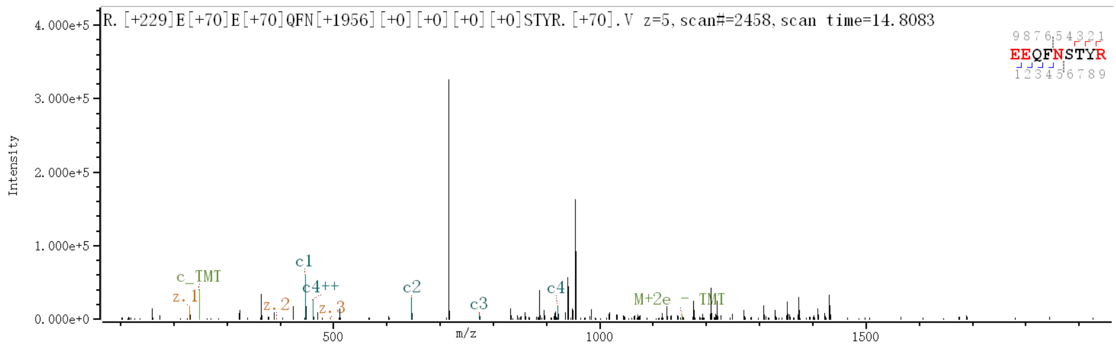

PID=898: EEQFNSTYR

#	a. calc.	c calc.	c++ calc.	c-HexN calc.	c-HexNAc calc.	Seq.	y calc.	z. calc.	z.++ calc.	#
1	402.3210	**446.3347**	223.6710			E				9
2	601.4526	**645.4663**	323.2368			E	3140.3765		1562.6825	8
3	729.5112	**773.5248**	387.2661			Q	2941.2449		1463.1167	7
4	876.5796	**920.5932**	**460.8003**			F	2813.1863		1399.0874	6
5	2946.3465	2990.3601	1495.6837			N	2666.1179	2650.0992	1325.5532	5
6	3033.3785	3077.3922	1539.1997			S	596.3510	580.3323	290.6698	4
7	3134.4262	3178.4399	1589.7236			T	509.3190	**493.3002**		3
8	3297.4895	3341.5032	1671.2552			Y	408.2713	**392.2526**		2
9						R	245.2080	**229.1892**		1

ETD MS2 spectrum of precursor m/z = 714.5393, z = 5^+ at 14.8083 min. This spectrum was assigned to the EEQFNSTYR with N-glycan composition of H4N5F2.

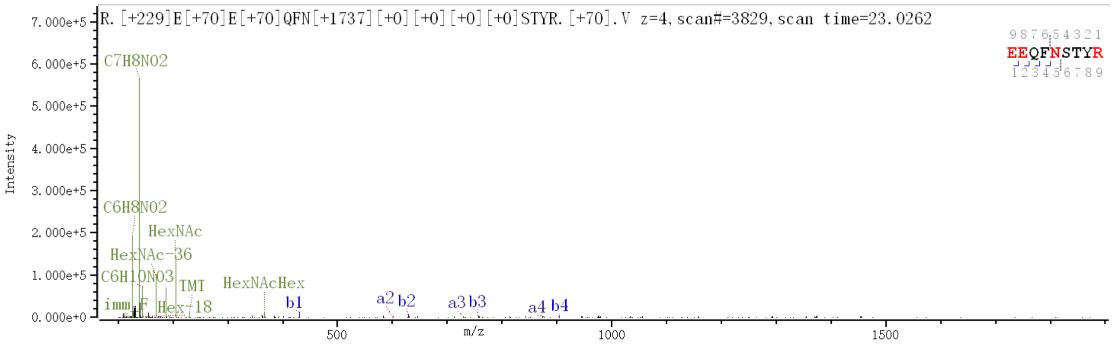

PID=3287: EEQFNSTYR

#	a calc.	b calc.	b-18 calc.	b++ calc.	b++ -HexNAc calc.	b++ -HexN calc.	b-HexN calc.	b_3+ calc.	Seq.	y calc.	y++ calc.	y_3+ calc.	#
1	401.3132	**429.3081**	411.2976	215.1577				143.7742	E				9
2	**600.4448**	**628.4397**	610.4291	314.7235				210.1514	E	2921.3022	1461.1547	974.4389	8
3	**728.5034**	**756.4983**	738.4877	378.7528				252.8376	Q	2722.1706	1361.5889	908.0617	7
4	**875.5718**	**903.5667**	885.5561	452.2870				301.8604	F	2594.1120	1297.5596	865.3755	6
5	2726.2644	2754.2593	2736.2488	1377.6333				918.7580	N	2447.0436	1224.0254	816.3527	5
6	2813.2964	2841.2913	2823.2808	1421.1493				947.7686	S	596.3510	298.6791	199.4552	4
7	2914.3441	2942.3390	2924.3285	1471.6732				981.4512	T	509.3190	255.1631	170.4445	3
8	3077.4074	3105.4024	3087.3918	1553.2048				1035.8056	Y	408.2713	204.6393	136.7619	2
9									R	245.2080	123.1076	82.4075	1

HCD MS2 spectrum of precursor m/z = 838.1587, z = 4^+ at 23.0262 min. This spectrum was assigned to the EEQFNSTYR with N-glycan composition of H3N4F3.

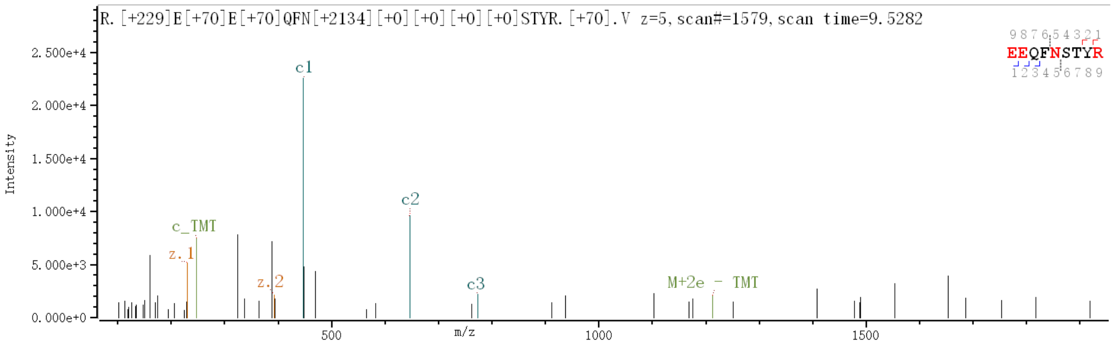

PID=1309: EEQFNSTYR

#	a. calc.	c calc.	c++ calc.	c-HexN calc.	c-HexNAc calc.	Seq.	y calc.	z. calc.	z.++ calc.	#
1	402.3210	**446.3347**	223.6710			E				9
2	601.4526	**645.4663**	323.2368			E	3318.4242		1651.7064	8
3	729.5112	**773.5248**	387.2661			Q	3119.2926		1552.1406	7
4	876.5796	920.5932	460.8003			F	2991.2340		1488.1113	6
5	3124.3942	3168.4079	1584.7076			N	2844.1656	2828.1469	1414.5771	5
6	3211.4263	3255.4399	1628.2236			S	596.3510	580.3323	290.6698	4
7	3312.4740	3356.4876	1678.7474			T	509.3190	493.3002		3
8	3475.5373	3519.5509	1760.2791			Y	408.2713	**392.2526**		2
9						R	245.2080	**229.1892**		1

ETD MS2 spectrum of precursor m/z = 750.1487, z = 4^+ at 9.5282 min. This spectrum was assigned to the EEQFNSTYR with N-glycan composition of H6N5F1.

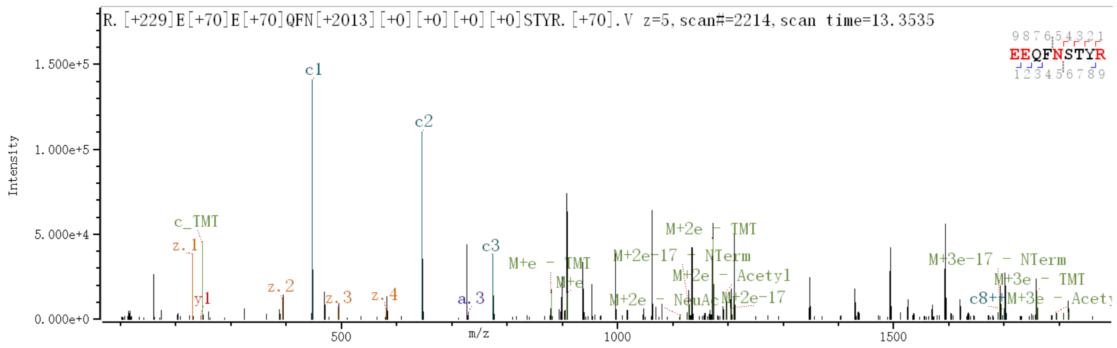

PID=1378: EEQFNSTYR

#	a. calc.	c calc.	c++ calc.	c-HexN calc.	c-HexNAc calc.	Seq.	y calc.	z. calc.	z.++ calc.	#
1	402.3210	**446.3347**	223.6710			E				9
2	601.4526	**645.4663**	323.2368			E	3197.3979		1591.1932	8
3	**729.5112**	**773.5248**	387.2661			Q	2998.2664		1491.6275	7
4	876.5796	920.5932	460.8003			F	2870.2078		1427.5982	6
5	3003.3680	3047.3816	1524.1944			N	2723.1394	2707.1206	1354.0640	5
6	3090.4000	3134.4136	1567.7105			S	596.3510	**580.3323**	290.6698	4
7	3191.4477	3235.4613	1618.2343			T	509.3190	**493.3002**		3
8	3354.5110	3398.5247	**1699.7660**			Y	408.2713	**392.2526**		2
9						R	**245.2080**	**229.1892**		1

ETD MS2 spectrum of precursor m/z = 725.9509, z = 5^+ at 13.3535 min. This spectrum was assigned to the EEQFNSTYR with N-glycan composition of H4N6F1.

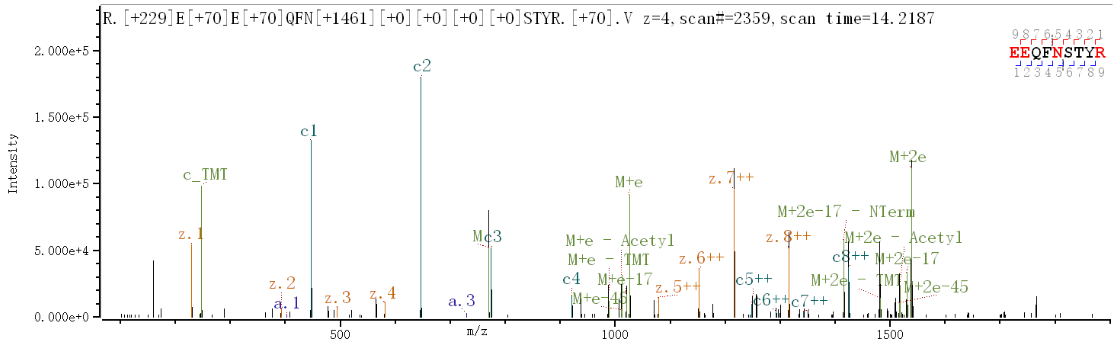

PID=375: EEQFNSTYR

#	a. calc.	c calc.	c++ calc.	c-HexN calc.	c-HexNAc calc.	Seq.	y calc.	z. calc.	z.++ calc.	#
1	**402.3210**	**446.3347**	223.6710			E				9
2	601.4526	**645.4663**	323.2368			E	2645.1813		**1315.0849**	8
3	**729.5112**	**773.5248**	387.2661			Q	2446.0497		**1215.5191**	7
4	876.5796	**920.5932**	460.8003			F	2317.9911		**1151.4898**	6
5	2451.1513	2495.1650	**1248.0861**			N	2170.9227	2154.9040	**1077.9556**	5
6	2538.1833	2582.1970	**1291.6021**			S	596.3510	**580.3323**	290.6698	4
7	2639.2310	2683.2447	**1342.1260**			T	509.3190	**493.3002**		3
8	2802.2944	2846.3080	**1423.6576**			Y	408.2713	**392.2526**		2
9						R	245.2080	**229.1892**		1

ETD MS2 spectrum of precursor m/z = 769.1262, z = 4^+ at 14.2187 min. This spectrum was assigned to the EEQFNSTYR with N-glycan composition of H4N4.

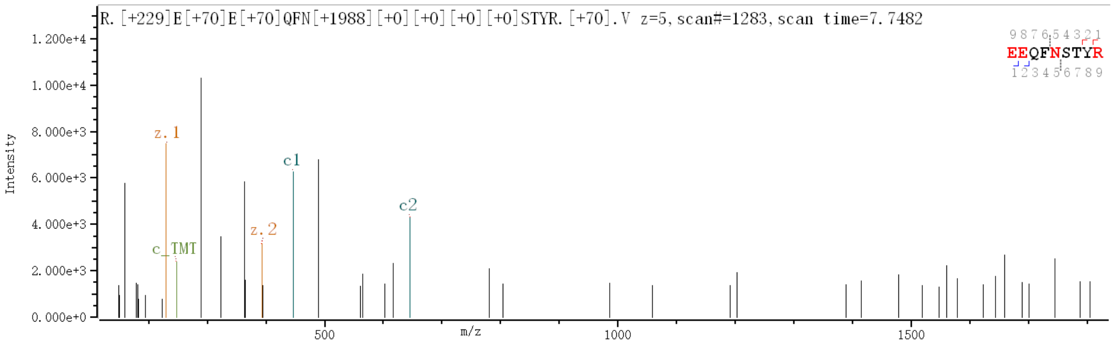

PID=706: EEQFNSTYR

#	a. calc.	c calc.	c++ calc.	c-HexN calc.	c-HexNAc calc.	Seq.	y calc.	z. calc.	z.++ calc.	#
1	402.3210	**446.3347**	223.6710			E				9
2	601.4526	**645.4663**	323.2368			E	3172.3663		1578.6774	8
3	729.5112	773.5248	387.2661			Q	2973.2347		1479.1116	7
4	876.5796	920.5932	460.8003			F	2845.1761		1415.0823	6
5	2978.3363	3022.3500	1511.6786			N	2698.1077	2682.0890	1341.5481	5
6	3065.3684	3109.3820	1555.1946			S	596.3510	580.3323	290.6698	4
7	3166.4160	3210.4297	1605.7185			T	509.3190	493.3002		3
8	3329.4794	3373.4930	1687.2501			Y	408.2713	**392.2526**		2
9						R	245.2080	**229.1892**		1

ETD MS2 spectrum of precursor m/z = 720.9346, z = 5^{+} at 7.7482 min. This spectrum was assigned to the EEQFNSTYR with N-glycan composition of H6N5.

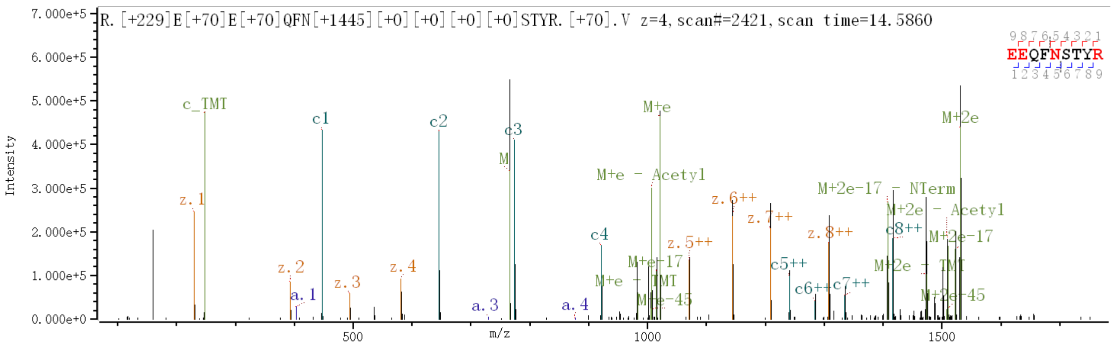

PID=387: EEQFNSTYR

#	a. calc.	c calc.	c++ calc.	c-HexN calc.	c-HexNAc calc.	Seq.	y calc.	z. calc.	z.++ calc.	#
1	**402.3210**	**446.3347**	223.6710			E				9
2	601.4526	**645.4663**	323.2368			E	2629.1864		**1307.0875**	8
3	**729.5112**	**773.5248**	387.2661			Q	2430.0548		**1207.5217**	7
4	**876.5796**	**920.5932**	460.8003			F	2301.9962		**1143.4924**	6
5	2435.1564	2479.1700	**1240.0887**			N	2154.9278	2138.9091	**1069.9582**	5
6	2522.1884	2566.2021	**1283.6047**			S	596.3510	**580.3323**	290.6698	4
7	2623.2361	2667.2498	**1334.1285**			T	509.3190	**493.3002**		3
8	2786.2994	2830.3131	**1415.6602**			Y	408.2713	**392.2526**		2
9						R	245.2080	**229.1892**		1

ETD MS2 spectrum of precursor m/z = 765.1275, z = 4^+ at 14.5860 min. This spectrum was assigned to the EEQFNSTYR with N-glycan composition of H3N4F1.

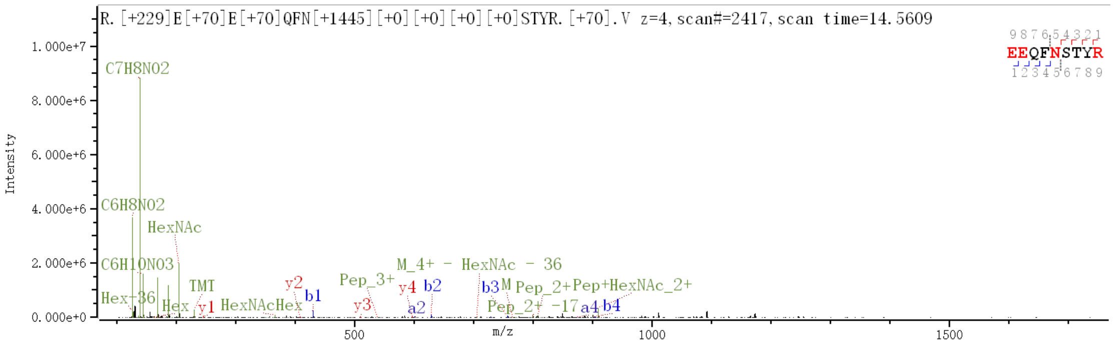

PID=2636: EEQFNSTYR

#	a calc.	b calc.	b-18 calc.	b++ calc.	b++ -HexNAc calc.	b++ -HexN calc.	b-HexN calc.	b_3+ calc.	Seq.	y calc.	y++ calc.	y_3+ calc.	#
1	401.3132	**429.3081**	411.2976	215.1577				143.7742	E				9
2	**600.4448**	**628.4397**	610.4291	314.7235				210.1514	E	2629.1864	1315.0968	877.0670	8
3	**728.5034**	**756.4983**	738.4877	378.7528				252.8376	Q	2430.0548	1215.5310	810.6898	7
4	**875.5718**	**903.5667**	885.5561	452.2870				301.8604	F	2301.9962	1151.5017	768.0036	6
5	2434.1486	2462.1435	2444.1329	1231.5754				821.3860	N	2154.9278	1077.9675	718.9808	5
6	2521.1806	2549.1755	2531.1650	1275.0914				850.3967	S	**596.3510**	298.6791	199.4552	4
7	2622.2283	2650.2232	2632.2126	1325.6152				884.0793	T	**509.3190**	255.1631	170.4445	3
8	2785.2916	2813.2865	2795.2760	1407.1469				938.4337	Y	**408.2713**	204.6393	136.7619	2
9									R	**245.2080**	123.1076	82.4075	1

HCD MS2 spectrum of precursor m/z = 765.1275, z = 4^{+} at 14.5609 min. This spectrum was assigned to the EEQFNSTYR with N-glycan composition of H3N4F1.

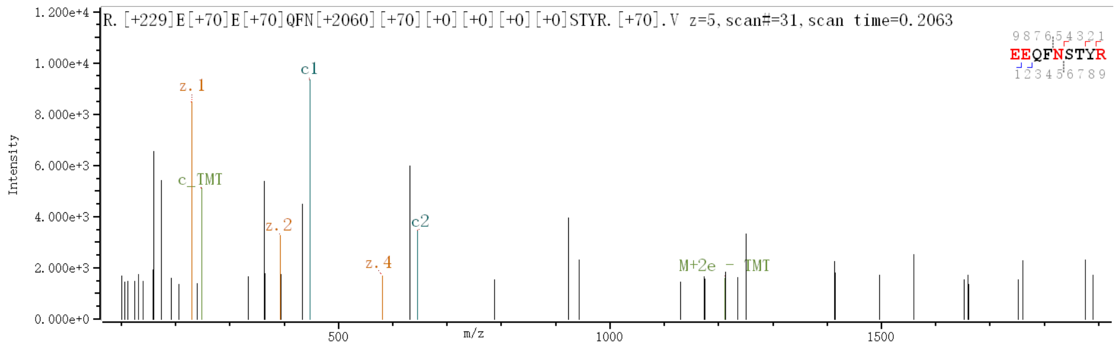

PID=1255: EEQFNSTYR

#	a. calc.	c calc.	c++ calc.	c-HexN calc.	c-HexNAc calc.	Seq.	y calc.	z. calc.	z.++ calc.	#
1	402.3210	**446.3347**	223.6710			E				9
2	601.4526	**645.4663**	323.2368			E	3314.4765		1649.7325	8
3	729.5112	773.5248	387.2661			Q	3115.3449		1550.1667	7
4	876.5796	920.5932	460.8003			F	2987.2864		1486.1375	6
5	3120.4466	3164.4602	1582.7337			N	2840.2180	2824.1992	1412.6033	5
6	3207.4786	3251.4922	1626.2498			S	596.3510	**580.3323**	290.6698	4
7	3308.5263	3352.5399	1676.7736			T	509.3190	493.3002		3
8	3471.5896	3515.6032	1758.3053			Y	408.2713	**392.2526**		2
9						R	245.2080	**229.1892**		1

ETD MS2 spectrum of precursor m/z = 749.3560, z = 5$^+$ at 0.2063 min. This spectrum was assigned to the EEQFNSTYR with N-glycan composition of H5N4F1S1.

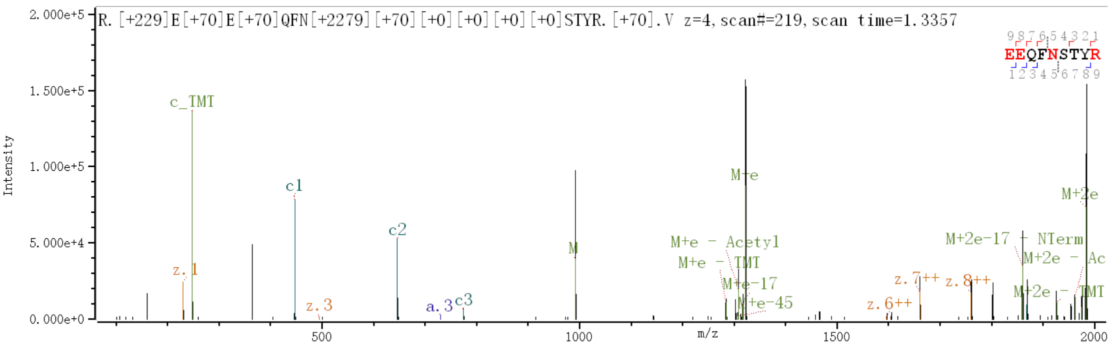

PID=1695: EEQFNSTYR

#	a. calc.	c calc.	c++ calc.	c-HexN calc.	c-HexNAc calc.	Seq.	y calc.	z. calc.	z.++ calc.	#
1	402.3210	**446.3347**	223.6710			E				9
2	601.4526	**645.4663**	323.2368			E	3533.5508		**1759.2697**	8
3	**729.5112**	**773.5248**	387.2661			Q	3334.4192		**1659.7039**	7
4	876.5796	920.5932	460.8003			F	3206.3607		**1595.6746**	6
5	3339.5209	3383.5345	1692.2709			N	3059.2922	3043.2735	1522.1404	5
6	3426.5529	3470.5665	1735.7869			S	596.3510	580.3323	290.6698	4
7	3527.6006	3571.6142	1786.3107			T	509.3190	**493.3002**		3
8	3690.6639	3734.6775	**1867.8424**			Y	408.2713	392.2526		2
9						R	245.2080	**229.1892**		1

ETD MS2 spectrum of precursor m/z = 991.2174, z = 4^+ at 1.3357 min. This spectrum was assigned to the EEQFNSTYR with N-glycan composition of H6N5S1.

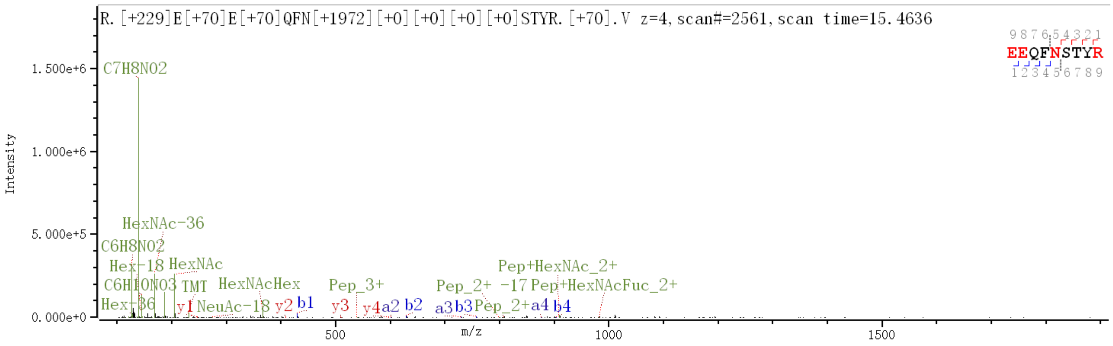

PID=3171: EEQFNSTYR

#	a calc.	b calc.	b-18 calc.	b++ calc.	b++ -HexNAc calc.	b++ -HexN calc.	b-HexN calc.	b_3+ calc.	Seq.	y calc.	y++ calc.	y_3+ calc.	#
1	401.3132	**429.3081**	411.2976	215.1577				143.7742	E				9
2	**600.4448**	**628.4397**	610.4291	314.7235				210.1514	E	3156.3714	1578.6893	1052.7953	8
3	**728.5034**	**756.4983**	738.4877	378.7528				252.8376	Q	2957.2398	1479.1235	986.4181	7
4	**875.5718**	**903.5667**	885.5561	452.2870				301.8604	F	2829.1812	1415.0942	943.7319	6
5	2961.3336	2989.3285	2971.3180	1495.1679				997.1144	N	2682.1128	1341.5600	894.7091	5
6	3048.3656	3076.3605	3058.3500	1538.6839				1026.1250	S	**596.3510**	298.6791	199.4552	4
7	3149.4133	3177.4082	3159.3977	1589.2078				1059.8076	T	**509.3190**	255.1631	170.4445	3
8	3312.4766	3340.4716	3322.4610	1670.7394				1114.1620	Y	**408.2713**	204.6393	136.7619	2
9									R	**245.2080**	123.1076	82.4075	1

HCD MS2 spectrum of precursor m/z = 896.9238, z = 4^{+} at 15.4636 min. This spectrum was assigned to the EEQFNSTYR with N-glycan composition of H5N5F1.

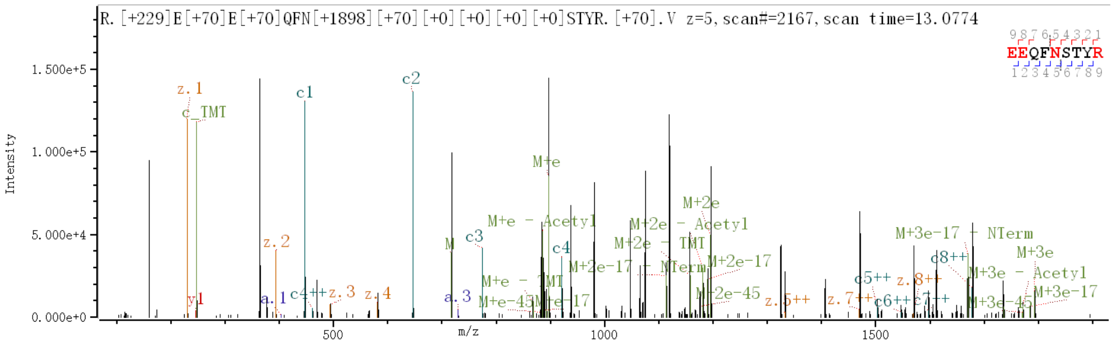

PID=879: EEQFNSTYR

#	a. calc.	c calc.	c++ calc.	c-HexN calc.	c-HexNAc calc.	Seq.	y calc.	z. calc.	z.++ calc.	#
1	**402.3210**	**446.3347**	223.6710			E				9
2	601.4526	**645.4663**	323.2368			E	3152.4237		**1568.7061**	8
3	**729.5112**	**773.5248**	387.2661			Q	2953.2921		**1469.1403**	7
4	876.5796	**920.5932**	**460.8003**			F	2825.2335		1405.1110	6
5	2958.3937	3002.4074	**1501.7073**			N	2678.1651	2662.1464	**1331.5768**	5
6	3045.4258	3089.4394	**1545.2233**			S	596.3510	**580.3323**	290.6698	4
7	3146.4735	3190.4871	**1595.7472**			T	509.3190	**493.3002**		3
8	3309.5368	3353.5504	**1677.2789**			Y	408.2713	**392.2526**		2
9						R	**245.2080**	**229.1892**		1

ETD MS2 spectrum of precursor m/z = 716.9518, z = 5^+ at 13.0774 min. This spectrum was assigned to the EEQFNSTYR with N-glycan composition of H4N4F1S1.

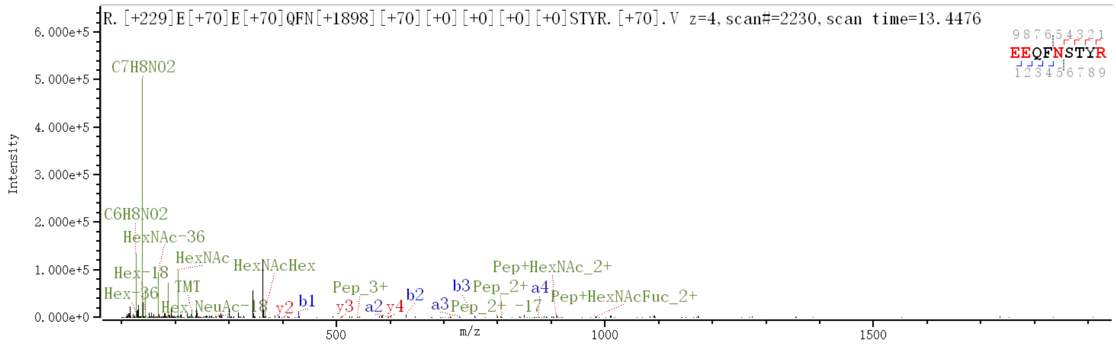

PID=3132: EEQFNSTYR

#	a calc.	b calc.	b-18 calc.	b++ calc.	b++ -HexNAc calc.	b++ -HexN calc.	b-HexN calc.	b_3+ calc.	Seq.	y calc.	y++ calc.	y_3+ calc.	#
1	401.3132	**429.3081**	411.2976	215.1577				143.7742	E				9
2	**600.4448**	**628.4397**	610.4291	314.7235				210.1514	E	3152.4237	1576.7155	1051.4794	8
3	**728.5034**	**756.4983**	738.4877	378.7528				252.8376	Q	2953.2921	1477.1497	985.1022	7
4	**875.5718**	**903.5667**	885.5561	452.2870				301.8604	F	2825.2335	1413.1204	942.4160	6
5	2957.3859	2985.3808	2967.3703	1493.1941				995.7985	N	2678.1651	1339.5862	893.3932	5
6	3044.4180	3072.4129	3054.4023	1536.7101				1024.8091	S	**596.3510**	298.6791	199.4552	4
7	3145.4656	3173.4605	3155.4500	1587.2339				1058.4917	T	**509.3190**	255.1631	170.4445	3
8	3308.5290	3336.5239	3318.5133	1668.7656				1112.8461	Y	**408.2713**	204.6393	136.7619	2
9									R	**245.2080**	123.1076	82.4075	1

HCD MS2 spectrum of precursor m/z = 895.9353, z = 4^+ at 13.4476 min. This spectrum was assigned to the EEQFNSTYR with N-glycan composition of H4N4F1S1.

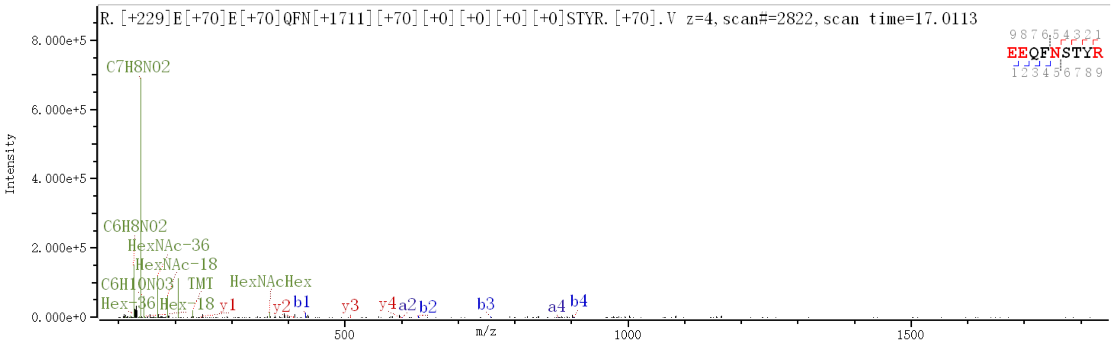

PID=3203: EEQFNSTYR

#	a calc.	b calc.	b-18 calc.	b++ calc.	b++ -HexNAc calc.	b++ -HexN calc.	b-HexN calc.	b_3+ calc.	Seq.	y calc.	y++ calc.	y_3+ calc.	#
1	401.3132	**429.3081**	411.2976	215.1577				143.7742	E				9
2	**600.4448**	**628.4397**	610.4291	314.7235				210.1514	E	2965.3393	1483.1733	989.1179	8
3	728.5034	**756.4983**	738.4877	378.7528				252.8376	Q	2766.2077	1383.6075	922.7407	7
4	**875.5718**	**903.5667**	885.5561	452.2870				301.8604	F	2638.1491	1319.5782	880.0545	6
5	2770.3015	2798.2964	2780.2858	1399.6518				933.4370	N	2491.0807	1246.0440	831.0317	5
6	2857.3335	2885.3284	2867.3178	1443.1678				962.4477	S	**596.3510**	298.6791	199.4552	4
7	2958.3812	2986.3761	2968.3655	1493.6917				996.1302	T	**509.3190**	255.1631	170.4445	3
8	3121.4445	3149.4394	3131.4289	1575.2233				1050.4847	Y	**408.2713**	204.6393	136.7619	2
9									R	**245.2080**	123.1076	82.4075	1

HCD MS2 spectrum of precursor m/z = 849.1580, z = 4^{+} at 17.0113 min. This spectrum was assigned to the EEQFNSTYR with N-glycan composition of H5N3S1.

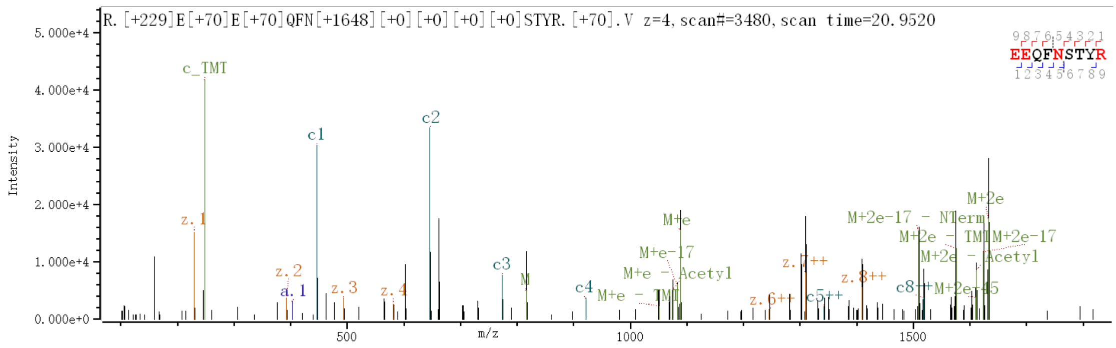

PID=442: EEQFNSTYR

#	a. calc.	c calc.	c++ calc.	c-HexN calc.	c-HexNAc calc.	Seq.	y calc.	z. calc.	z.++ calc.	#
1	**402.3210**	**446.3347**	223.6710			E				9
2	601.4526	**645.4663**	323.2368			E	2832.2657		**1408.6271**	8
3	729.5112	**773.5248**	387.2661			Q	2633.1342		**1309.0614**	7
4	876.5796	**920.5932**	460.8003			F	2505.0756		**1245.0321**	6
5	2638.2358	2682.2494	**1341.6283**			N	2358.0072	2341.9884	1171.4979	5
6	2725.2678	2769.2814	1385.1444			S	596.3510	**580.3323**	290.6698	4
7	2826.3155	2870.3291	1435.6682			T	509.3190	**493.3002**		3
8	2989.3788	3033.3925	**1517.1999**			Y	408.2713	**392.2526**		2
9						R	245.2080	**229.1892**		1

ETD MS2 spectrum of precursor m/z = 815.8985, z = 4^+ at 20.9520 min. This spectrum was assigned to the EEQFNSTYR with N-glycan composition of H3N5F1.

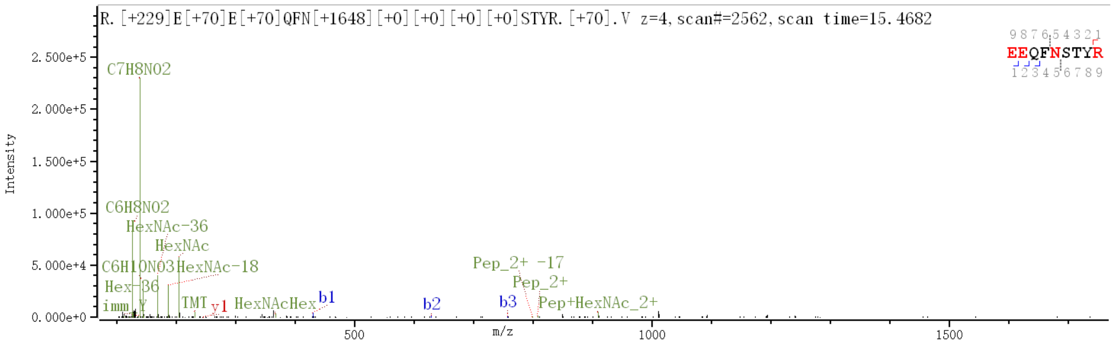

PID=2649: EEQFNSTYR

#	a calc.	b calc.	b-18 calc.	b++ calc.	b++ -HexNAc calc.	b++ -HexN calc.	b-HexN calc.	b_3+ calc.	Seq.	y calc.	y++ calc.	y_3+ calc.	#
1	401.3132	**429.3081**	411.2976	215.1577				143.7742	E				9
2	600.4448	**628.4397**	610.4291	314.7235				210.1514	E	2832.2657	1416.6365	944.7601	8
3	728.5034	**756.4983**	738.4877	378.7528				252.8376	Q	2633.1342	1317.0707	878.3829	7
4	875.5718	903.5667	885.5561	452.2870				301.8604	F	2505.0756	1253.0414	835.6967	6
5	2637.2280	2665.2229	2647.2123	1333.1151				889.0791	N	2358.0072	1179.5072	786.6739	5
6	2724.2600	2752.2549	2734.2443	1376.6311				918.0898	S	596.3510	298.6791	199.4552	4
7	2825.3077	2853.3026	2835.2920	1427.1549				951.7724	T	509.3190	255.1631	170.4445	3
8	2988.3710	3016.3659	2998.3553	1508.6866				1006.1268	Y	408.2713	204.6393	136.7619	2
9									R	**245.2080**	123.1076	82.4075	1

HCD MS2 spectrum of precursor m/z = 815.8931, z = 4^{+} at 15.4682 min. This spectrum was assigned to the EEQFNSTYR with N-glycan composition of H3N5F1.

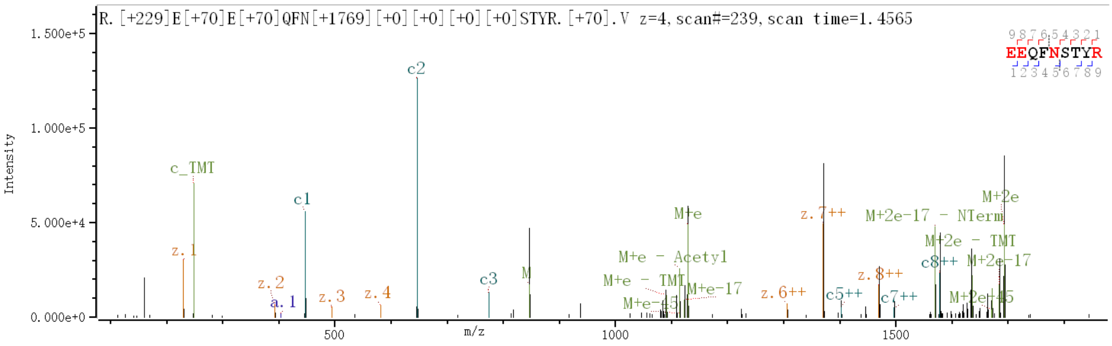

PID=597: EEQFNSTYR

#	a. calc.	c calc.	c++ calc.	c-HexN calc.	c-HexNAc calc.	Seq.	y calc.	z. calc.	z.++ calc.	#
1	**402.3210**	**446.3347**	223.6710			E				9
2	601.4526	**645.4663**	323.2368			E	2953.2920		**1469.1403**	8
3	729.5112	**773.5248**	387.2661			Q	2754.1604		**1369.5745**	7
4	876.5796	920.5932	460.8003			F	2626.1019		**1305.5452**	6
5	2759.2621	2803.2757	**1402.1415**			N	2479.0334	2463.0147	1232.0110	5
6	2846.2941	2890.3077	1445.6575			S	596.3510	**580.3323**	290.6698	4
7	2947.3418	2991.3554	**1496.1813**			T	509.3190	**493.3002**		3
8	3110.4051	3154.4187	**1577.7130**			Y	408.2713	**392.2526**		2
9						R	245.2080	**229.1892**		1

ETD MS2 spectrum of precursor m/z = 846.1512, z = 4^+ at 1.4565 min. This spectrum was assigned to the EEQFNSTYR with N-glycan composition of H5N4F1.

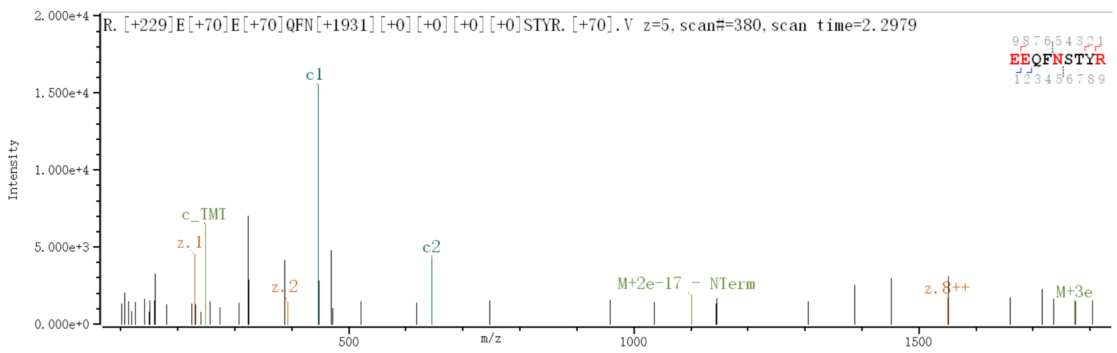

PID=625: EEQFNSTYR

#	a. calc.	c calc.	c++ calc.	c-HexN calc.	c-HexNAc calc.	Seq.	y calc.	z. calc.	z.++ calc.	#
1	402.3210	**446.3347**	223.6710			E				9
2	601.4526	**645.4663**	323.2368			E	3115.3448		**1550.1667**	8
3	729.5112	773.5248	387.2661			Q	2916.2133		1450.6009	7
4	876.5796	920.5932	460.8003			F	2788.1547		1386.5716	6
5	2921.3149	2965.3285	1483.1679			N	2641.0863	2625.0675	1313.0374	5
6	3008.3469	3052.3605	1526.6839			S	596.3510	580.3323	290.6698	4
7	3109.3946	3153.4082	1577.2078			T	509.3190	493.3002		3
8	3272.4579	3316.4716	1658.7394			Y	408.2713	**392.2526**		2
9						R	245.2080	**229.1892**		1

ETD MS2 spectrum of precursor m/z = 709.5337, z = 5^{+} at 2.2979 min. This spectrum was assigned to the EEQFNSTYR with N-glycan composition of H6N4F1.

Tables

Table S1 The N-glycan database of IgG.

No.	Glycan composition
1	HexNAc(10)Hex(11) @ NGlycan | common1
2	HexNAc(2)Hex(11) @ NGlycan | common1
3	HexNAc(2)Hex(12) @ NGlycan | common1
4	HexNAc(2)Hex(3) @ NGlycan | common1
5	HexNAc(2)Hex(3)Fuc(1) @ NGlycan | common1
6	HexNAc(2)Hex(4) @ NGlycan | common1
7	HexNAc(2)Hex(5) @ NGlycan | common1
8	HexNAc(2)Hex(6) @ NGlycan | common1
9	HexNAc(2)Hex(7) @ NGlycan | common1
10	HexNAc(2)Hex(8) @ NGlycan | common1
11	HexNAc(2)Hex(9) @ NGlycan | common1
12	HexNAc(2)Hex(10) @ NGlycan | common1
13	HexNAc(3)Hex(3)Fuc(1)NeuAc(1) (70.089) @ NGlycan | common1
14	HexNAc(3)Hex(3) @ NGlycan | common1
15	HexNAc(3)Hex(3)Fuc(1) @ NGlycan | common1
16	HexNAc(3)Hex(3)Fuc(1)NeuAc(2) (140.178) @ NGlycan | common1
17	HexNAc(3)Hex(3)NeuAc(1) @ NGlycan | common1
18	HexNAc(3)Hex(4) @ NGlycan | common1
19	HexNAc(3)Hex(4)Fuc(1) @ NGlycan | common1
20	HexNAc(3)Hex(4)Fuc(1)NeuAc(1) (70.089) @ NGlycan | common1
21	HexNAc(3)Hex(4)Fuc(2) @ NGlycan | common1
22	HexNAc(3)Hex(4)NeuAc(1) (70.089) @ NGlycan | common1
23	HexNAc(3)Hex(4)NeuAc(2) (140.178) @ NGlycan | common1
24	HexNAc(3)Hex(5) @ NGlycan | common1
25	HexNAc(3)Hex(5)Fuc(1) @ NGlycan | common1
26	HexNAc(3)Hex(5)Fuc(1)NeuAc(1) (70.089)@ NGlycan | common1
27	HexNAc(3)Hex(5)NeuAc(1) (70.089) @ NGlycan | common1
28	HexNAc(3)Hex(5)NeuAc(2) (140.178) @ NGlycan | common1
29	HexNAc(3)Hex(6) @ NGlycan | common1
30	HexNAc(3)Hex(6)Fuc(1) @ NGlycan | common1
31	HexNAc(3)Hex(6)Fuc(1)NeuAc(1) (70.089) @ NGlycan | common1
32	HexNAc(3)Hex(6)NeuAc(1) (70.089) @ NGlycan | common1
33	HexNAc(3)Hex(6)NeuAc(2) (140.178)@ NGlycan | common1
34	HexNAc(3)Hex(7)Fuc(1) @ NGlycan | common1
35	HexNAc(3)Hex(7)Fuc(1)NeuAc(1) (70.089) @ NGlycan | common1
36	HexNAc(3)Hex(7)NeuAc(1) (70.089) @ NGlycan | common1
37	HexNAc(3)Hex(7)NeuAc(2) (140.178) @ NGlycan | common1

38 HexNAc(3)Hex(8)NeuAc(1) (70.089) @ NGlycan | common1
39 HexNAc(3)Hex(8)NeuAc(2) (140.178)@ NGlycan | common1
40 HexNAc(3)Hex(9)Fuc(2) @ NGlycan | common1
41 HexNAc(4)Hex(11)Fuc(1)NeuAc(4) (280.356) @ NGlycan | common1
42 HexNAc(4)Hex(11)NeuAc(1) @ NGlycan | common1
43 HexNAc(4)Hex(14)Fuc(1)NeuAc(1) (70.089) @ NGlycan | common1
44 HexNAc(4)Hex(14)Fuc(3)NeuAc(1) (70.089) @ NGlycan | common1
45 HexNAc(4)Hex(14)NeuAc(1) (70.089) @ NGlycan | common1
46 HexNAc(4)Hex(3) @ NGlycan | common1
47 HexNAc(4)Hex(3)Fuc(1) @ NGlycan | common1
48 HexNAc(4)Hex(3)Fuc(2) @ NGlycan | common1
49 HexNAc(4)Hex(3)Fuc(1)NeuAc(1) (70.089) @ NGlycan | common1
50 HexNAc(4)Hex(3)Fuc(3) @ NGlycan | common1
51 HexNAc(4)Hex(3)NeuAc(1) (70.089) @ NGlycan | common1
52 HexNAc(4)Hex(4) @ NGlycan | common1
53 HexNAc(4)Hex(4)Fuc(1) @ NGlycan | common1
54 HexNAc(4)Hex(4)Fuc(1)NeuAc(1) (70.089) @ NGlycan | common1
55 HexNAc(4)Hex(4)Fuc(3) @ NGlycan | common1
56 HexNAc(4)Hex(4)NeuAc(1) (70.089) @ NGlycan | common1
57 HexNAc(4)Hex(4)NeuAc(2) (140.178)@ NGlycan | common1
58 HexNAc(4)Hex(5) @ NGlycan | common1
59 HexNAc(4)Hex(5)Fuc(1) @ NGlycan | common1
60 HexNAc(4)Hex(5)Fuc(2) @ NGlycan | common1
61 HexNAc(4)Hex(5)Fuc(1)NeuAc(1) (70.089) @ NGlycan | common1
62 HexNAc(4)Hex(5)Fuc(1)NeuAc(2) (140.178) @ NGlycan | common1
63 HexNAc(4)Hex(5)Fuc(2)NeuAc(2) (140.178) @ NGlycan | common1
64 HexNAc(4)Hex(5)Fuc(3) @ NGlycan | common1
65 HexNAc(4)Hex(5)NeuAc(1) (70.089) @ NGlycan | common1
66 HexNAc(4)Hex(5)NeuAc(2) (140.178) @ NGlycan | common1
67 HexNAc(4)Hex(6) @ NGlycan | common1
68 HexNAc(4)Hex(6)Fuc(1) @ NGlycan | common1
69 HexNAc(4)Hex(6)Fuc(1)NeuAc(1) (70.089) @ NGlycan | common1
70 HexNAc(4)Hex(6)Fuc(1)NeuAc(2) (140.178) @ NGlycan | common1
71 HexNAc(4)Hex(6)Fuc(3)NeuAc(1) (70.089) @ NGlycan | common1
72 HexNAc(4)Hex(6)NeuAc(1) (70.089) @ NGlycan | common1
73 HexNAc(4)Hex(6)NeuAc(2) (140.178) @ NGlycan | common1
74 HexNAc(4)Hex(7) @ NGlycan | common1
75 HexNAc(4)Hex(7)NeuAc(1) (70.089) @ NGlycan | common1
76 HexNAc(5)Hex(10)NeuAc(1) (70.089) @ NGlycan | common1
77 HexNAc(5)Hex(10)NeuAc(2) (140.178) @ NGlycan | common1
78 HexNAc(5)Hex(11)Fuc(1)NeuAc(4) (280.356) @ NGlycan | common1
79 HexNAc(5)Hex(13)Fuc(3)NeuAc(1) (70.089) @ NGlycan | common1
80 HexNAc(5)Hex(3) @ NGlycan | common1
81 HexNAc(5)Hex(3)Fuc(1) @ NGlycan | common1

82	HexNAc(5)Hex(4) @ NGlycan | common1
83	HexNAc(5)Hex(4)Fuc(1) @ NGlycan | common1
84	HexNAc(5)Hex(4)Fuc(1)NeuAc(1) (70.089) @ NGlycan | common1
85	HexNAc(5)Hex(4)Fuc(2) @ NGlycan | common1
86	HexNAc(5)Hex(4)Fuc(2)NeuAc(1) (70.089) @ NGlycan | common1
87	HexNAc(5)Hex(4)Fuc(2)NeuAc(2) (140.178) @ NGlycan | common1
88	HexNAc(5)Hex(4)NeuAc(1) (70.089) @ NGlycan | common1
89	HexNAc(5)Hex(4)NeuAc(2) (140.178) @ NGlycan | common1
90	HexNAc(5)Hex(5) @ NGlycan | common1
91	HexNAc(5)Hex(5)Fuc(1) @ NGlycan | common1
92	HexNAc(5)Hex(5)Fuc(1)NeuAc(1) (70.089) @ NGlycan | common1
93	HexNAc(5)Hex(5)Fuc(1)NeuAc(2) (140.178) @ NGlycan | common1
94	HexNAc(5)Hex(5)Fuc(2) @ NGlycan | common1
95	HexNAc(5)Hex(5)Fuc(2)NeuAc(1) (70.089) @ NGlycan | common1
96	HexNAc(5)Hex(5)NeuAc(1) (70.089) @ NGlycan | common1
97	HexNAc(5)Hex(5)NeuAc(2) (140.178) @ NGlycan | common1
98	HexNAc(5)Hex(6) @ NGlycan | common1
99	HexNAc(5)Hex(6)Fuc(1) @ NGlycan | common1
100	HexNAc(5)Hex(6)Fuc(1)NeuAc(1) (70.089) @ NGlycan | common1
101	HexNAc(5)Hex(6)Fuc(1)NeuAc(2) (140.178) @ NGlycan | common1
102	HexNAc(5)Hex(6)Fuc(1)NeuAc(3) (210.267) @ NGlycan | common1
103	HexNAc(5)Hex(6)Fuc(2)NeuAc(3) (210.267) @ NGlycan | common1
104	HexNAc(5)Hex(6)Fuc(2)NeuAc(5) (350.445) @ NGlycan | common1
105	HexNAc(5)Hex(6)NeuAc(1) (70.089) @ NGlycan | common1
106	HexNAc(5)Hex(6)NeuAc(2) (140.178)@ NGlycan | common1
107	HexNAc(5)Hex(6)NeuAc(3) @ NGlycan | common1
108	HexNAc(5)Hex(6)NeuAc(4) @ NGlycan | common1
109	HexNAc(5)Hex(7)Fuc(1) @ NGlycan | common1
110	HexNAc(5)Hex(7)Fuc(1)NeuAc(1) (70.089)@ NGlycan | common1
111	HexNAc(5)Hex(7)Fuc(1)NeuAc(3) (210.267) @ NGlycan | common1
112	HexNAc(5)Hex(7)Fuc(3)NeuAc(2) (140.178) @ NGlycan | common1
113	HexNAc(5)Hex(7)NeuAc(1) (13.031) @ NGlycan | common1
114	HexNAc(5)Hex(8)Fuc(1)NeuAc(1) (70.089) @ NGlycan | common1
115	HexNAc(5)Hex(8)Fuc(2)NeuAc(1) (70.089) @ NGlycan | common1
116	HexNAc(5)Hex(8)Fuc(4)NeuAc(1) (70.089) @ NGlycan | common1
117	HexNAc(5)Hex(8)NeuAc(1) (13.031) @ NGlycan | common1
118	HexNAc(6)Hex(10)Fuc(5)NeuAc(3) (210.267) @ NGlycan | common1
119	HexNAc(6)Hex(4)Fuc(1) @ NGlycan | common1
120	HexNAc(6)Hex(6)Fuc(1) @ NGlycan | common1
121	HexNAc(6)Hex(6)Fuc(1)NeuAc(1) (70.089) @ NGlycan | common1
122	HexNAc(6)Hex(6)Fuc(1)NeuAc(2) (140.178) @ NGlycan | common1
123	HexNAc(6)Hex(6)Fuc(1)NeuAc(3) (210.267) @ NGlycan | common1
124	HexNAc(6)Hex(6)Fuc(3) @ NGlycan | common1
125	HexNAc(6)Hex(6)Fuc(3)NeuAc(1) (70.089) @ NGlycan | common1

126	HexNAc(6)Hex(7) @ NGlycan | common1
127	HexNAc(6)Hex(7)Fuc(1)NeuAc(1) (70.089) @ NGlycan | common1
128	HexNAc(6)Hex(7)Fuc(1)NeuAc(2) (140.178) @ NGlycan | common1
129	HexNAc(6)Hex(7)Fuc(1)NeuAc(3) (210.267) @ NGlycan | common1
130	HexNAc(6)Hex(7)Fuc(1)NeuAc(4) (280.356) @ NGlycan | common1
131	HexNAc(6)Hex(7)Fuc(2)NeuAc(1) (70.089) @ NGlycan | common1
132	HexNAc(6)Hex(7)Fuc(2)NeuAc(2) (140.178) @ NGlycan | common1
133	HexNAc(6)Hex(7)Fuc(2)NeuAc(3) (210.267) @ NGlycan | common1
134	HexNAc(6)Hex(7)Fuc(2)NeuAc(4) (280.356) @ NGlycan | common1
135	HexNAc(6)Hex(7)Fuc(3)NeuAc(1) (70.089) @ NGlycan | common1
136	HexNAc(6)Hex(7)NeuAc(1) (70.089) @ NGlycan | common1
137	HexNAc(6)Hex(7)NeuAc(2) (140.178) @ NGlycan | common1
138	HexNAc(6)Hex(7)NeuAc(3) (210.267) @ NGlycan | common1
139	HexNAc(6)Hex(7)NeuAc(4) (280.356) @ NGlycan | common1
140	HexNAc(6)Hex(8)Fuc(1)NeuAc(1) (70.089) @ NGlycan | common1
141	HexNAc(6)Hex(8)Fuc(1)NeuAc(2) (140.178) @ NGlycan | common1
142	HexNAc(6)Hex(8)Fuc(1)NeuAc(3) (210.267) @ NGlycan | common1
143	HexNAc(6)Hex(8)Fuc(2)NeuAc(2) (140.178) @ NGlycan | common1
144	HexNAc(6)Hex(8)Fuc(3)NeuAc(3) (210.267) @ NGlycan | common1
145	HexNAc(7)Hex(10)Fuc(4)NeuAc(1) (70.089) @ NGlycan | common1
146	HexNAc(7)Hex(11)Fuc(4)NeuAc(1) (70.089) @ NGlycan | common1
147	HexNAc(7)Hex(12)Fuc(1)NeuAc(4) (280.356) @ NGlycan | common1
148	HexNAc(7)Hex(12)Fuc(4)NeuAc(1) (70.089) @ NGlycan | common1
149	HexNAc(7)Hex(13)Fuc(2)NeuAc(1) (70.089) @ NGlycan | common1
150	HexNAc(7)Hex(5) @ NGlycan common1
151	HexNAc(7)Hex(5)Fuc(1)NeuAc(1) (70.089) @ NGlycan | common1
152	HexNAc(7)Hex(6)Fuc(2)NeuAc(6) (420.534) @ NGlycan | common1
153	HexNAc(7)Hex(7)NeuAc(3) (210.267) @ NGlycan | common1
154	HexNAc(7)Hex(8) @ NGlycan | common1
155	HexNAc(7)Hex(8)Fuc(1)NeuAc(2) (140.178) @ NGlycan | common1
156	HexNAc(7)Hex(8)Fuc(2)NeuAc(3) (210.267) @ NGlycan | common1
157	HexNAc(7)Hex(8)Fuc(3)NeuAc(3) (210.267) @ NGlycan | common1
158	HexNAc(7)Hex(9)Fuc(1)NeuAc(2) (140.178) @ NGlycan | common1
159	HexNAc(7)Hex(9)Fuc(4)NeuAc(2) (140.178) @ NGlycan | common1
160	HexNAc(7)Hex(9)NeuAc(1) (70.089) @ NGlycan | common1
161	HexNAc(8)Hex(8)Fuc(2) @ NGlycan | common1
162	HexNAc(8)Hex(9)Fuc(1) @ NGlycan | common1
163	HexNAc(9)Hex(9)Fuc(1) @ NGlycan | common1

Table S2 MS2 setting

	HCD settings (NCE in MS2)	ETD settings
1	Single energy 40%	Trigger
2	Single energy 50%	Trigger
3	40% ±10%	Directly
4	50% ±10%	Directly
5	40% ±10%	Trigger
6	50% ±10%	Trigger

Note: Table S3- S13 are provided as separate files.

References

1. Yang L, Sun ZY and Zhang L *et al.* Chemical labeling for fine mapping of IgG N-glycosylation by ETD-MS. *Chem. Sci.* 2019; **10**: 9302-7.

2. Riley NM, Malaker SA and Driessen MD *et al.* Optimal Dissociation Methods Differ for N- and O-Glycopeptides. *J. Proteome Res.* 2020; **19**: 3286-301.

3. Wang JR, Gao WN and Grimm R *et al.* A method to identify trace sulfated IgG N-glycans as biomarkers for rheumatoid arthritis. *Nat. Commun.* 2017; **8**: 631.

4. Yuan W, Sanda M and Wu J *et al.* Quantitative analysis of immunoglobulin subclasses and subclass specific glycosylation by LC-MS-MRM in liver disease. *J. Proteomics* 2015; **116**: 24-33.

5. Di Y, Zhang L and Zhang Y *et al.* MdCDPM: A Mass Defect-Based Chemical-Directed Proteomics Method for Targeted Analysis of Intact Sialylglycopeptides. *Anal. Chem.* 2019; **91**: 9986-92.

6. Bern M, Kil YJ and Becker C. Byonic: advanced peptide and protein identification software. *Curr. Protoc. Bioinformatics* New Jersey: Wiley, 2012; **Chapter 13**: Unit13 20.

7. Yu GC, Wang LG and Han YY *et al*. clusterProfiler: an R Package for Comparing Biological Themes Among Gene Clusters. *OMICS* 2012; **16**: 284-7.

8. Futschik ME, Carlisle B. Noise-robust soft clustering of gene expression time-course data. *J. Bioinform. Comput. Biol.* 2005; **3**: 965-88.

9. Culhane AC, Thioulouse J and Perriere G *et al*. MADE4: an R package for multivariate analysis of gene expression data. *Bioinformatics* 2005; **21**: 2789-90.
